# Optimizing lung cancer diagnosis using improved fungal growth optimizer-based medical image segmentation

**DOI:** 10.1038/s41598-026-58817-z

**Published:** 2026-07-02

**Authors:** Asmaa M. Khalid, Shimaa M. Abdel-Moniem, Nabil A. Lashin

**Affiliations:** Department of Information Technology, Faculty of Computers and Informatics, Zagazig University, Zagazig, 44519 Egypt

**Keywords:** Multi-level Threshold segmentation, Optimization, Lung cancer, Convergence, Orthogonal Learning, Cancer, Computational biology and bioinformatics, Engineering, Mathematics and computing

## Abstract

Medical image segmentation is one of the most important processes in computer-aided diagnosis. It plays a critical role in detecting and analyzing diseases since it isolates the region of interest in medical scans. Out of many techniques, multilevel thresholding is capable of segmenting complex images. It isolates important structures with multiple intensity thresholds. The effectiveness of multilevel thresholding depends on the optimization algorithm’s capacity to identify the appropriate threshold levels. This paper proposes an effective Fungal Growth Optimizer (FGO) with Orthogonal Learning Strategy (OLS). We emphasize enhancing solution diversity and speeding up convergence in multilevel thresholding-based medical image segmentation. The proposed algorithm, referred to as OLFGO, is tested on a set of 20 chest CT images from an openly available lung cancer database. We use various threshold values (2, 4, 6, 8, 10, and 12) to test its performance at different levels of segmentation. To assess the segmentation performance, we use several evaluation criteria such as Peak Signal-to-Noise Ratio (PSNR), Structural Similarity Index Measure (SSIM), Feature Similarity Index Measure (FSIM), Universal Quality Index (UQI) and Dice coefficient (DICE). Moreover, the results are compared with deep learning algorithm’s outcomes. We have demonstrated the reliability and efficacy of the OLFGO algorithm after implementing OLS using the Congress on Evolutionary Computation (CEC 2022) benchmarks. The improvement significantly enhanced the performance of the algorithm. The experiments confirm the efficiency of orthogonal learning in enforcing FGO’s ability towards effective and reliable medical image segmentation. We also employed the Friedman rank sum test to rank the performance of OLFGO against existing techniques. OLFGO was ranked first, which reconfirms its superior ability in image segmentation. The MATLAB implementation of the proposed OLFGO is available at : https://github.com/shimaa21magdy-sketch/Orthogonal-Learning-Fungal-Growth-Optimizer-for-Lung-CT-Segmentation.

## Introduction

Lung cancer remains among the primary and most severe forms of cancer worldwide, causing approximately 1.8 million annual deaths^1^. Diagnosis and treatment become more challenging in advanced stages; therefore, early detection helps speed up treatment and cure larger number of patients. Chest CT imaging is among the most frequently applied diagnostic modalities owing to the fact that it is accessible, low-cost, and non-invasive. But manual reading of chest CT images is time-consuming and prone to human error, especially in early lung cancer where the abnormalities may be obscured and easily overlooked^2,3^. To fill this gap, increasing interest has been demonstrated in computer-aided diagnosis (CAD) systems that assist radiologists in recognizing and analyzing with efficiency pulmonary abnormalities. A crucial component in CAD is medical image segmentation that involves the division of an image into relevant regions such as segmenting tumors or abnormal tissue from normal tissue^4^. In medical imaging, image segmentation is a major step in improving cancer detection and localization because of the following reasons:

- It splits the image into meaningful areas thus isolating the infected region from healthy lung tissue.
- It helps detect early-stage cancer that might not be observed by looking at scans.
- It detects exact tumor boundaries.
- It reduces radiologists’ errors by avoiding visual judgments.
- It can be used to track changes in tumor volume over time.

Among various methods of segmentation, multilevel thresholding has gained widespread popularity owing to its capacity to handle complex image structures and several intensity levels^5^. This technique functions by selecting an optimal range of thresholds dividing the image into distinct classes, thereby facilitating easier visualization and analysis. With more thresholds, the search space becomes increasingly complex, thereby making the determination of optimum thresholds computationally expensive. To mitigate this, researchers have used metaheuristic optimization techniques such as Genetic Algorithms (GA), Particle Swarm Optimization (PSO), and Gray Wolf Optimizer (GWO), which bypass threshold choice and provide robust solutions^6–8^. Another new and very promising algorithm is the Fungal Growth Optimizer (FGO), inspired by the adaptive and efficient growth of fungal hyphae^9^. Although FGO has been successful in solving global optimization issues, its use in medical image segmentation has revealed several disadvantages like gradual convergence rates, poor exploration capacities, and the tendency to get trapped in local optima^10^. To eliminate these issues, this paper presents a new development: the Orthogonal Learning-based Fungal Growth Optimizer (OLFGO). This extension incorporates Orthogonal Learning Strategies (OLS) into the general FGO framework to promote population diversity and convergence behavior. Orthogonal learning has been effective in other metaheuristic algorithms by aggressively searching more informative regions of the search space and hence avoiding premature convergence and improving global search ability^11,12^. The proposed OLFGO algorithm is designed for efficient and accurate multilevel thresholding of medical image segmentation for lung cancer identification based on chest CT images. The addition of OLS enhances FGO’s exploration and exploitation balance, accelerates convergence and improves segmentation accuracy without compromising its bio-inspired essence. To evaluate the performance of OLFGO, experiments were presented on 20 chest CT images from an open lung cancer data set, with threshold values of 2, 4, 6, 8, 10, and 12 to test segmentation performance at various complexities. We evaluated how effective the algorithm is by looking at five standard image quality metrics: Peak Signal-to-Noise Ratio (PSNR), Structural Similarity Index Measure (SSIM), Feature Similarity Index Measure (FSIM), Universal Quality Index (UQI)^13,14^ in addition to calculating the value of Dice coefficient (DICE)^15^ in order to measure the degree of similarity between the obtained segmentations and the truth masks, which are then compared against the results of advanced segmentation models using deep learning techniques. Furthermore, to compare its overall optimization capacity, OLFGO was also tested on the CEC2022 test functions, which are commonly used to measure how robust optimization algorithms are across different types of problems^16^. Comparative research employing cutting edge optimization algorithms proved that OLFGO consistently surpassed others both in terms of quality of segmentation and convergence speed. Statistical confirmation through Friedman rank sum test also strongly confirmed OLFGO’s superiority by assigning it the highest average rank. These findings confirm that OLFGO is a reliable, efficient, and robust optimization framework, which can further enhance automated medical image processing and significantly enhance early lung cancer diagnosis. We can conclude the main contributions in the following points:

- A novel OLFGO is proposed that combines Orthogonal Learning Strategy (OLS) and Fungal Growth Optimizer (FGO) to enhance solution convergence and diversity.
- OLFGO algorithm is applied to detect lung cancer in chest CT images using multilevel thresholding.
- In order to obtain the most unbiased and comprehensive assessment of the results five well-known metrics are deployed including PSNR, SSIM, FSIM, UQI and DICE.
- The overall OLFGO capability is validated using the CEC2022 benchmark functions.
- Comparative assessment with eight new metaheuristic algorithms: FGO, FATA, SOA, ZOA, COVID, AOA, RSA, and GTO.
- Comparative assessment with deep learning algorithms: Attention U-Net model, CNN model.
- Friedman statistical testing confirms that OLFGO performs the best among all algorithms compared.

### Related work

The development of effective medical image segmentation methods has been a point of much interest in current times owing to their paramount significance in computer-aided diagnosis, particularly for diseases such as lung cancer. Most prior work has focused on multilevel thresholding, evolutionary algorithms, and hybrid schemes to enhance segmentation performance. Classic segmentation methods such as Otsu’s technique and Kapur’s entropy approach have also been employed heavily for multilevel and bi-level thresholding applications^17,18^. Although convenient for simple scenarios, these methods are computationally heavy exponentially with more thresholds and impractical for use with high resolution medical images. To overcome these limitations, scientists have witnessed a growing trend towards the application of metaheuristic optimization methods. For example, Particle Swarm Optimization (PSO) and Genetic Algorithms (GA) have found broad utilization in the improvement of threshold selection and segmentation precision^7,8^. Also, Artificial Bee Colony (ABC)^19^, Differential Evolution (DE)^20^, and Ant Colony Optimization (ACO)^21^ algorithms have been investigated for their robust search ability in high-dimensional solution spaces. Nature-inspired algorithms such as the Grey Wolf Optimizer (GWO)^9^, Whale Optimization Algorithm (WOA)^22^, and Aquila Optimizer (AO)^23^ have recently appeared with enhanced performance in exploration-exploitation balance as well as convergence speed^24–27^. “Quantum-inspired” methods that were first designed showed that integrating quantum behaviors into population search can stabilize exploration as well as improve threshold selection. Surprisingly, certain versions of quantum-behaved particle swarm optimization (QPSO) have been used to optimize Otsu/Kapur thresholds for multilevel thresholding in medical images with better robustness than conventional PSO with noise or low-contrast images^28^. More recent reviews also position quantum and other nature-inspired methods as viable candidates for medical segmentation in general^29^. Of more recent metaheuristics proposed, the Reptile Search Algorithm (RSA) and its variants have performed extremely well on grayscale, multi-level thresholding. Hybrid RSA e.g. RSA with Salp Swarm (RSA-SSA) showed regular PSNR/SSIM gains and highest Friedman rank on test images, including COVID-19 radiography^30^. The newer “IRSA” architectures employ elitist/Gbest learning to accelerate convergence and segmentation quality further^31^. Clustering pipelines are generally supported by global search. A representative example is Optimized K-Means with an Improved Hybrid Grey Wolf Optimization (IGWO-SOA) initialization for managing exploration and end-phase optimization; this IGK-means stack has been shown to improve PSNR/FSIM/IoU compared to vanilla K-means and other hybrids^32^. Such ideas are part of a broader movement combining K-means and swarm intelligence in the hopes of decreasing sensitivity to initial centers^33^. For lung cancer, a number of optimizers have been utilized to the untypical intensity profiles and inhomogeneous boundaries on CT and pathology images. Multilevel thresholding on lung cancer pathological images has been regulated by the improved RIME optimization algorithm with improved segmentation accuracy and robustness than control swarm algorithms^34^. In parallel, Improved lung cancer segmentation based on nature-inspired Optimization integrates K-means with ABC/PSO/FFA/CSA, whose CSA-supported variant performed best in precision, sensitivity, and accuracy for CT scans^35^. Region-based pipelines remain valid as well: Enhanced/Improved Statistical Region Growing (E-SRG) accelerates seed selection and reduces growth-stage computations and improves practical robustness for lung CT processing^36,37^. Finally, new PSO variants continue targeting multilevel thresholding using Kapur entropy, validating the applicability of metaheuristic search to medical segmentation^38^. But despite these breakthroughs, most of these algorithms are still susceptible to premature convergence, particularly with extremely complicated or noisy clinical images, such as chest CT images for lung cancer diagnosis. To counteract this, scientists have proposed Orthogonal Learning Strategies (OLS), which were effectively integrated with conventional algorithms such as PSO^11^ and DE^12^ to maximize population diversity and reduce redundant exploration. However, in the past there have been no studies combining OLS and the Fungal Growth Optimizer (FGO), a more recent algorithm based on the exploratory growth and adaptability of fungal networks^9^. FGO has been found to be competitive for many optimization challenges since it provides an effective local exploitation vs. global exploration trade-off. Nonetheless, it is not yet adequately explored in medical image segmentation, and existing implementations usually lack richness, get stuck early on, and fail to be robust on diverse medical datasets. To address this, while several segmentation algorithms have been evaluated on medical datasets, most are not adequately evaluated with standard global optimization benchmarks, such as the CEC2022 benchmark suite, a well-known test functions used for testing general purpose optimization algorithm performance^16^. The requirement for a successful hybrid strategy that makes use of the adaptability of FGO and the diversity-promoting qualities of OLS further highlights this gap. For this reason, we propose the Orthogonal Learning based Fungal Growth Optimizer (OLFGO), in which OLS is embedded within the FGO framework to optimize convergence behavior and segmentation outcome. The above mentioned OLFGO is not just evaluated on real chest CT images for the diagnosis of lung cancer, but also on CEC2022 benchmark functions to ensure its overall optimization robustness and scalability across various problem domains.

### Motivation and contribution

Lung cancer remains among the most prevalent and fatal cancers worldwide, causing millions of deaths every year. Proper and early diagnosis is required to increase treatment success. Chest CT images serve as one of the major preliminary screening tools due to its cost savings, availability, and brief acquisition time^39^. Interpretation of CT by hand, is subjective, susceptible to inter observer variation, and involves considerable radiological experience, particularly in identifying small or subtle abnormalities^40^. This has introduced a growing focus on the need for strong and automated medical image segmentation techniques, especially within computer-aided diagnosis (CAD) systems. Multilevel thresholding is one of the widely used segmentation methods in medical imaging because it can distinguish multiple regions of interest based on pixel intensity values^41^. The segmentation quality is very sensitive to optimal threshold values which are difficult to determine in any problem and computationally expensive if there are many thresholds. Classical methods such as Otsu’s and Kapur’s methods are efficient for bi-level thresholding it is not in high-dimensional threshold selection problems^17,18^.With a view to overcoming these limitations, several metaheuristic optimization algorithms have been proposed, including Particle Swarm Optimization (PSO)^7^, Genetic Algorithms (GA)^8^, Whale Optimization Algorithm (WOA)^22^, and Aquila Optimizer (AO)^23^, whose aim is to automate the threshold selection process and improve its performance. While these algorithms yield better exploration of the search space, they are also plagued by premature convergence, local optimum stagnation, and lack of solution diversity, all of which can adversely affect segmentation outcomes, especially in noisy or complex medical images.

One new and promising algorithm from such a perspective is the FGO, a bio-inspired metaheuristic algorithm that is motivated by the adaptive growth of fungi hyphae^9^. Although FGO has shown strong performance on a variety of global optimization tasks, straight application of FGO to medical image segmentation is not yet developed. FGO also does not have the capacity to preserve population diversity and conduct the search process well, which plays a vital role in high-quality segmentation. In order to address the above limitations, we propose a new algorithm OLFGO which integrates an Orthogonal Learning Strategy (OLS) into FGO. OLS improves the candidate solution diversity and speeds up convergence by producing more informed individuals with the help of orthogonal arrays^11,42^. The main objective is to leverage the complementary knowledge of FGO and OLS to find a more complete, precise, and generalizable algorithm for various medical image segmentation problems and broader optimization tasks. To evaluate the effectiveness of the new OLFGO, the algorithm is applied to multilevel thresholding-based image segmentation of 20 chest CT images from a publicly available lung cancer database. Threshold levels 2, 4, 6, 8, 10, and 12 are set to perform experiments for measuring performance at various levels of segmentation complexity. Five widely used image quality metrics Peak Signal-to-Noise Ratio (PSNR), Structural Similarity Index Measure (SSIM), Feature Similarity Index Measure (FSIM), Universal Quality Index (UQI) and Dice coefficient (DICE) are utilized to measure the effectiveness of the proposed approach^13–15^. While the PSNR, SSIM, FSIM, and UQI metrics serve different purposes in assessing image quality, the Dice coefficient serves as the main metric for measuring the degree of similarity between the segmented images and their corresponding ground truth masks. Moreover, for a complete assessment of the proposed OLFGO, its performance is benchmarked against some other popular state-of-the-art deep-learning based segmentation approaches. Furthermore, to judge the overall optimization capability of OLFGO, it is run on the CEC2022 benchmark functions, a standard suit for testing the effectiveness of optimization algorithms on various types of problems^16^. An exhaustive comparative study is performed employing eight novel and competitive optimization algorithms: Fungal Growth Optimizer (FGO) essentially motivated by the growth of fungi in nature. Fungal growth includes hyphal growth, branching, and spore germination. Hyphal growth emulates hyphal extension and chemotropism to effectively explore the search space and identify regions with nutrients^9^, Fata Morgana Algorithm (FATA) is an effective swarm intelligence algorithm employed for solving continuous multi-type optimization problems. It mimics the process of mirage to create the mirage light filtering idea (MLF) and the strategy of light propagation (LPS)^43^, Seagull Optimization Algorithm (SOA) is used for overcoming costly computational problems. The algorithm is motivated mainly by seagulls’ migratory as well as hunting behavior. These tasks are modeled mathematically and employed to focus on searching and exploiting a provided search space^44^, Zebra Optimization Algorithm (ZOA) draws its main inspiration from the natural world and the behavior of zebras. ZOA mimics zebras’ foraging behaviors and the anti-predator strategies^45^, Coronavirus Optimization Algorithm (COVID) is an evolutionary optimization algorithm that simulates the process of how coronaviruses infect human cells^46^, Arithmetic Optimization Algorithm (AOA) employs the distributional behavior of the principal arithmetic operators in mathematics, e.g., Multiplication, Division, Subtraction, and Addition. AOA is mathematically modelled and employed to perform the optimization actions in a vast range of search spaces^47^, Reptile Search Algorithm (RSA) is inspired by the stalking techniques of crocodiles. The behavior of a crocodile is carried out in two broad stages: encircling, which is done by high walking or belly walking, and hunting, which is done by hunting coordination or cooperation^48,49^, and Giant Trevally Optimizer (GTO) Seabirds (sooty terns), fish, and cephalopods are some of the many animals giant trevally prey upon in the wild.

Statistical confirmation based on the Friedman rank sum test confirms that OLFGO has the best overall performance with improved segmentation capability and general-purpose optimization stability. In addition to optimization-based segmentation approaches deep learning models are widely used for medical image segmentation. This is because they can learn features directly from data. In this study we use two known deep learning models: a Convolutional Neural Network (CNN)-based segmentation model^50^ and the Attention U-Net^51^. We compare these models with our proposed OLFGO approach. The CNN-based model uses a convolutional architecture. It has convolutional and pooling layers to extract features. Then it has up sampling layers to create -wise segmentation maps. This model serves as a deep learning approach. It provides a framework for learning spatial features from chest CT images. However traditional CNN architectures may not be very accurate. This is because they lose information during down sampling. To improve this, we also use the Attention U- model. It is based on the UNet architecture, which was proposed by Olaf Ranneberger^52^. The U-Net is an encoder-decoder network for biomedical image segmentation. The Attention U-Net adds attention mechanisms to the connections. This helps the model focus on feature maps. It ignores background information. This mechanism allows the model to focus on regions of interest such as tumor areas in chest CT images. This improves segmentation accuracy in cases with low contrast or small target regions. By using both CNN and Attention U-Net models we can compare optimization-based segmentation with modern deep learning-based approaches. This helps us evaluate our proposed OLFGO method. We can see how effective it is in handling datasets and complex segmentation tasks compared to data-driven models. The OLFGO approach is useful for image segmentation tasks. It can handle datasets and complex tasks. The CNN and Attention U-Net models are also useful for these tasks. They provide a comparison with the OLFGO approach. However deep learning models like CNN and Attention U-Net have their limitations. They require a lot of data to train. They can be computationally expensive. The OLFGO approach can be more efficient. It can handle datasets. It can provide results. Overall, the OLFGO approach is a tool for medical image segmentation. It can handle tasks. It can provide results. The CNN and Attention U-Net models are also useful. They provide a comparison, with the OLFGO approach.

## Preliminaries

This section provides the theoretical background and major elements upon which the new OLFGO is proposed. They are the basic principles of the FGO, the OLS, and the mathematical formulation of multilevel thresholding based on Otsu’s variance-based criterion.

### Multilevel thresholding

Thresholding is one of the simplest and most common algorithms for image segmentation. It operates directly on an image’s grayscale histogram with the objective to segment regions based on different pixel intensity values. The simplicity and ease of computation involved in thresholding have rendered it attractive to a wide range of applications, particularly biomedical image processing^5^. Thresholding algorithms can be generally divided into two main classes: parametric and non-parametric methods.

- Parametric thresholding assumes that intensity values in each class (region) follow a specified probability distribution most often a Gaussian distribution. The issue is to estimate the parameters of the distribution (e.g., mean and variance) so that the aggregate histogram of the image closely agrees with the modeled data^53^.
- Non-parametric thresholding techniques consider pixel intensity distributions without making any assumptions. They use statistical quantification of thresholds based upon entropy, inter-class variance, or similarity indices. The focus of these techniques is to decompose the image into sharp, homogeneous, and distinct classes while avoiding any ambiguity or overlap between the classes^18^.

Non-parametric techniques are most suitable for complex or natural images where pixel intensity distributions may be multimodal or unknown, which is generally the case for images in medical imaging. Among these, Otsu’s technique is an often-used technique which selects threshold values maximizing inter-class variance and thereby maximally improves class separability^17^.

Techniques can also be classified based on the number of thresholds as follows:

- Bi-level thresholding separates the image into two regions (background and fore-ground) using a single threshold.
- Multilevel thresholding uses multiple thresholds to classify an image into multiple classes. This method is especially useful for the segmentation of intricate medical images that feature multiple lesion stages or different types of tissues^53^.

For many thresholds, computational cost of verification of all permutations becomes exponential. Combinatorial explosion renders exhaustive search methods impractical for high-resolution images so optimization-based techniques, especially metaheuristic algorithms, have to be employed in order to efficiently detect near-optimal threshold values^5,6,41^.

The goal in multilevel thresholding is to find the optimal threshold set $\:\{{h}_{1},{h}_{2},\dots\:,{h}_{d}\}$ that maximizes the total between-class variance, defined as:

1 $$\:f\left({h}_{0},{h}_{1},\dots\:,{h}_{d+1}\right)={\sigma\:}_{0}^{2}+{\sigma\:}_{1}^{2}+\cdots\:+{\sigma\:}_{d}^{2}$$

where:$\:{h}_{0}=0$ and $\:{h}_{d+1}=255$ represent the minimum and maximum intensity levels in the image, and$\:{\sigma\:}_{j}^{2}$ denotes the between-class variance of class $\:j$ for $\:j=0,1,\dots\:,d$.

Each class is defined over the intensity interval $\:[{h}_{j},{h}_{j+1}]$, and the between-class variance for each class is computed as:

2 $$\:{\sigma\:}_{j}^{2}={\omega\:}_{j}({\mu\:}_{j}-{\mu\:}_{T}{)}^{2}$$

where:$\:{\omega\:}_{j}$ is the probability (weight) of class $\:j$,$\:{\mu\:}_{j}$ is the mean gray level of class $\:j$,$\:{\mu\:}_{T}$ is the global mean intensity of the entire image.

These components are calculated using the following equations:

3 $$\:{\omega\:}_{j}=\sum\:_{i={h}_{j}}^{{h}_{j+1}-1}{p}_{i}$$

4 $$\:{\mu\:}_{j}=\frac{1}{{\omega\:}_{j}}\sum\:_{i={h}_{j}}^{{h}_{j+1}-1}i\cdot\:{p}_{i}$$

5 $$\:{\mu\:}_{T}=\sum\:_{i=0}^{255}i\cdot\:{p}_{i}$$

where:$\:{p}_{i}=\frac{{P}_{i}}{W}$ is the normalized histogram probability for intensity level $\:i$,$\:{P}_{i}$ is the number of pixels with gray level $\:i$,$\:W$ is the total number of pixels in the image.

The threshold set $\:\{{h}_{1},{h}_{2},\dots\:,{h}_{d}\}$ divides the image into $\:d+1\:\:$non**-**overlapping classes, each representing a homogeneous region. The optimization objective is to maximize the function $\:f(\cdot\:)$ from Eq. (2), thereby achieving the best possible separation between different regions of the image. The OLFGO framework does take on the particular case of multilevel optimization, especially on the threshold values $\:\{{h}_{1},\dots\:,{h}_{d}\}$. For the optimization process, the Otsu-based objective function explained before is used on every candidate solution to the problem of segmentation to try to obtain the best possible segmentation.

### Optimization algorithm: fungal growth optimizer (FGO)

Fungal Growth Optimizer (FGO) is a nature-inspired metaheuristic algorithm inspired by the adaptive foraging and spreading behavior of fungi particularly mycelium growth dynamics of the fungus. It is designed to address complex, stochastic, and high-dimensional optimization problems through the equilibrium between exploration (searching around unvisited areas) and exploitation (focusing search in the vicinity of known good solutions)^9^. Fungi grow by means of specialized organs called hyphae long, branching filaments which elongate towards sources of nutrients in the environment. FGO reflects this natural process with three main strategies:

1. Hyphal Tip Growth: Mimics directional searching, simulating growth of hyphae to-ward rising nutrient levels.
2. Lateral Branching: For local search, generates new candidate solutions in the Lateral Branching promotes local search by hyphal regions, akin to the lateral growth of fungal hyphae.
3. Spore Germination: Introduces random candidate solutions to improve global diversity, aiding the algorithm in escaping local optima, thus augmenting overall robustness.

FGO also employs an adaptive nutrient allocation mechanism, where the quantity of nutrient absorbed by each solution is determined from its fitness value. The more fit the solution is, the more nutrients are allocated to it, and this guides the growth process and reinforces the search in prospective regions^9^. This biologically inspired framework allows FGO to have a proper balance between intensification and diversification and thus can solve a lot of diverse global optimization problems. That said, its application in medical image segmentation is still limited and not fully explored.

#### FGO algorithm framework

Let $\:{\overrightarrow{S}}_{i}^{t}\in\:{\mathbb{R}}^{d}$ denote the position of the $\:i$-th solution (or hyphal agent) in the solution space at iteration $\:t$, and let $\:f\left({\overrightarrow{S}}_{i}^{t}\right)$ denote its corresponding fitness. The FGO approach mimics the development, adjustment, and the reproductive methods of fungal tubers to rapidly penetrate the available space for a solution and converge to the global solution. This is achieved through a series of organized steps:

- Step 1: Initialization
  The first step of the algorithm involves the random selection of *N* candidate solutions (i.e. hyphal solutions) and the placement of the hyphal structures within the boundaries of the problem:
  6 $$\:{\overrightarrow{S}}_{i}={\overrightarrow{S}}_{L}+\overrightarrow{r}\odot\:\left({\overrightarrow{S}}_{U}-{\overrightarrow{S}}_{L}\right),\:i=1,2,\dots\:,N$$
  where: $\:{\overrightarrow{S}}_{L},{\overrightarrow{S}}_{U}\in\:{\mathbb{R}}^{d}$: Lower and upper bounds of the search space.$\:\overrightarrow{r}\in\:[0,1{]}^{d}$: Uniformly distributed random vector.$\:\odot\:$: Element-wise multiplication.
  This helps to ensure the proper seeding of the initial search agents, all of which are spatially evenly distributed in relation to one another.
- Step 2: Hyphal Tip Growth (Exploration).
  Every agent makes a determination to either go in search of new areas to exploit or stay within confines of already well-defined territorial structures. This is based on two different parameters:
  - Fitness Normalization.
    A normalized fitness probability $\:{p}_{i}$ is calculated to represent the relative quality of each solution:
    7 $$\:{p}_{i}=\frac{{f}_{i}-\mathrm{m}\mathrm{i}\mathrm{n}\left(f\right)}{\mathrm{m}\mathrm{a}\mathrm{x}\left(f\right)-\mathrm{m}\mathrm{i}\mathrm{n}\left(f\right)+\epsilon\:}$$
    where:$\:{f}_{i}=f\left({\overrightarrow{S}}_{i}^{t}\right)$, the fitness of the current solution.$\:\epsilon\:$: Small positive constant to avoid division by zero.
  - Dynamic exploration rate.
    An adaptive threshold $\:{E}_{r}$ is computed to control the transition from exploration to exploitation over time:
    8 $$\:{E}_{r}=M+\left(1-M\right)\left(1-\frac{t}{{t}_{\mathrm{m}\mathrm{a}\mathrm{x}}}\right)$$
    where:$\:M\in\:[0,1]$: Minimum exploration threshold.$\:{t}_{\mathrm{m}\mathrm{a}\mathrm{x}}$: Maximum number of iterations.
    As $\:t$ increases, $\:{E}_{r}$ decreases, favoring exploitation in later stages.
    If $\:{p}_{i}<{E}_{r}$, the solution undergoes nutrient-driven directional growth.
  - Growth Energy Factor.
    There is a growth energy score, *E*, that is computed which is based on the spatial architecture of the nutrients and adaptive decay over time:
    9 $$\:F=\frac{{f}_{i}}{\sum\:_{k=1}^{N}{f}_{k}}\cdot\:{r}_{1}\cdot\:{\left(1-\frac{t}{{t}_{\mathrm{m}\mathrm{a}\mathrm{x}}}\right)}^{1-\frac{t}{{t}_{\mathrm{m}\mathrm{a}\mathrm{x}}}}$$
    10 $$\:E=\mathrm{e}\mathrm{x}\mathrm{p}\left(F\right)$$
    where:$\:{r}_{1}\in\:[0,1]$: Random growth modulation parameter. This mechanism mimics biological hyphae accelerating toward richer nutrient zones.
  - Growth Direction Update.
    Using the computed energy, the agent grows directionally:
    11 $$\:{\overrightarrow{S}}_{i}^{t+1}={\overrightarrow{S}}_{i}^{t}+E\cdot\:\left({\overrightarrow{S}}_{a}-{\overrightarrow{S}}_{b}\right)$$
    where:$\:{\overrightarrow{S}}_{a},{\overrightarrow{S}}_{b}$: Two randomly selected distinct solutions.$\:({\overrightarrow{S}}_{a}-{\overrightarrow{S}}_{b})$: Directional vector encouraging diverse movement.
- Step 3: Chemotropism and Exploitation.
  If $\:{p}_{i}\ge\:{E}_{r}$, the solution performs local refinement using chemotropic attraction:
  12 $$\:{\overrightarrow{D}}_{e}={r}_{4}\left({\overrightarrow{S}}_{a}-{\overrightarrow{S}}_{i}\right)+{r}_{5}\cdot\:\beta\:\left({\overrightarrow{S}}^{\mathrm{*}}-{\overrightarrow{S}}_{i}\right)\cdot\:\left[{r}_{6}>R\right]$$
  13 $$\:{\overrightarrow{S}}_{i}^{t+1}={\overrightarrow{S}}_{i}+{\eta\:}_{i}\cdot\:{\overrightarrow{D}}_{e}+{\overrightarrow{E}}_{c}\cdot\:\left[{r}_{12}>{E}_{p}\right]$$
  where:$\:{\overrightarrow{S}}^{\mathrm{*}}$: Global best solution.$\:\beta\:$: Scaling factor.$\:{\eta\:}_{i}$: Local nutrient influence.$\:{\overrightarrow{E}}_{c}$: Environmental fluctuation vector.$\:[r>x]$: Iverson bracket (1 if true, 0 otherwise), used for stochastic switching.
  This simulates chemotropic sensitivity i.e., the fungal tip turning toward nutrient-rich areas.
- Step 4: Lateral Branching.
  Branching introduces local diversity around the current solution.
  - Difference Vectors
    14 $$\:{\overrightarrow{D}}_{\mathrm{ep1}}={\overrightarrow{S}}_{b}-{\overrightarrow{S}}_{c},\:{\overrightarrow{D}}_{\mathrm{ep2}}={\overrightarrow{S}}_{a}-{\overrightarrow{S}}^{\mathrm{*}}$$
  - Branching Update
    15 $$\:{\overrightarrow{S}}_{i}^{t+1}={\overrightarrow{S}}_{i}+{r}_{5}\cdot\:{\overrightarrow{D}}_{\mathrm{e}\mathrm{p}1}+\left(1-{r}_{5}\right)\cdot\:{\overrightarrow{D}}_{\mathrm{e}\mathrm{p}2}$$
  This allows the solution to explore its local neighborhood in multiple directions.
- Step 5: Spore Germination (Global Search).
  To preserve exploration and prevent stagnation, spores are produced through stochastic recombination of existing solutions:
  16 $$\:{S}_{i,j}^{t+1}=\frac{{S}_{a}\left(j\right)+{S}_{b}\left(j\right)+{S}_{c}\left(j\right)}{3}+\mu\:\cdot\:\left|\frac{{S}^{\mathrm{*}}\left(j\right)+{S}_{b}\left(j\right)}{2}-{S}_{i}\left(j\right)\right|$$
  Or alternatively:
  17 $$\:{S}_{i,j}^{t+1}=\frac{3{S}_{a}\left(j\right)+{S}_{b}\left(j\right)+{S}_{c}\left(j\right)}{5}+\mu\:\cdot\:\left(2{S}^{\mathrm{*}}\left(j\right)+{S}_{b}\left(j\right)-{S}_{i}\left(j\right)\right)$$
  where:$\:\mu\:=\pm\:r\cdot\:E$ is a stochastic offset.
- Step 6: Selection and Replacement.
  After generating $\:{S}_{i}^{t+1}$, its fitness is evaluated. If it improves upon the previous solution, it is retained; otherwise, the old solution is restored. The global best solution $\:{S}^{\mathrm{*}}$ is updated accordingly.

**Algorithm 1:**
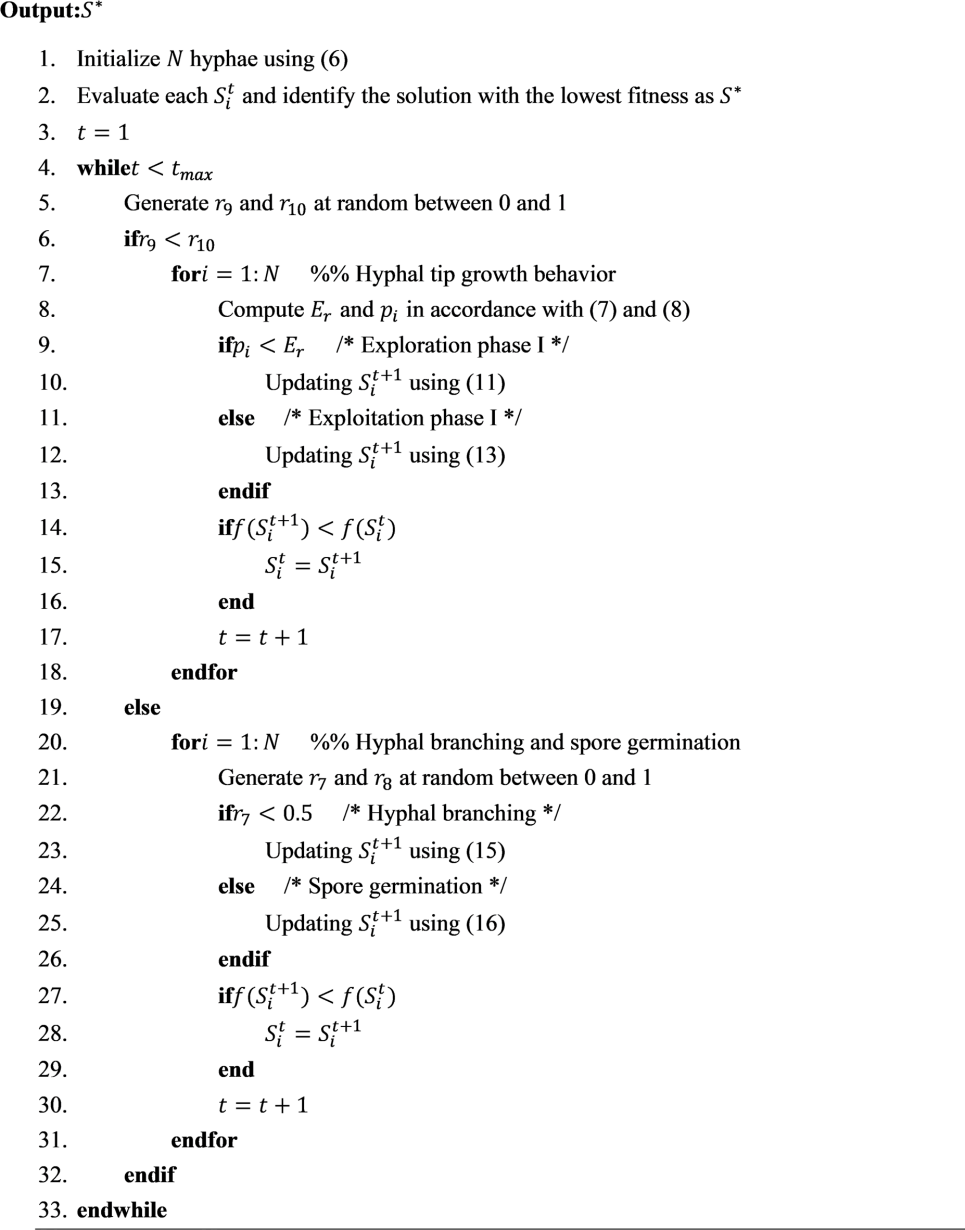
Pseudocode of FGO.

The FGO algorithm has a number of benefits, such as: Effective worldwide search: Made possible by branching processes that improve exploration and spore germination, Robust optimization is ensured by adaptive convergence control, which skillfully strikes a balance between extensive local search and wider exploration and Implementation simplicity: FGO is easy to tune and use, requiring fewer control parameters than many other swarm-inspired algorithms.

### Orthogonal-based learning strategy

In metaheuristic optimization, orthogonal-based learning (OL) is a potent enhancement technique that is frequently used to improve the balance between exploration and exploitation in complex search spaces^11,42^. The FGO algorithm and OL are combined in this study to create an efficient hybrid strategy known as OLFGO. By methodically investigating a large number of potential solution elements, OL aims to direct the optimization process toward more promising areas of the search space. Orthogonal Experimental Design (OED), a statistical framework that guarantees effective sampling and varied solution generation, is used to accomplish this methodical investigation. OED functions in two primary phases^9,53^:

1. The orthogonal array (OA) Construction: An orthogonal matrix is used to create a representative subset of solutions in this step. The objective is to efficiently and uniformly sample the multidimensional solution space.
2. Factor Analysis (FA): At this stage, the contribution of each variable (factor) towards the objective function is approximated in order to identify the best combinations in an effort to guarantee enhanced con-vergence and enhanced decision-making.

This two-phase process provides a structured, computationally efficient exploitation mechanism for better convergence rate and accuracy without increasing algorithmic complexity or the number of control parameters. Orthogonal learning has been effectively implemented in various applications, including medical diagnosis and image processing^11,53^, and has been incredibly successful when combined with metaheuristics such as PSO, DE, and more recently, RIME^54^. Its integration with FGO in the presented OLFGO algorithm provides higher search flexibility, resistance to local optimum, and enhanced segmentation efficiency in sophisticated medical imaging applications.

#### Orthogonal Array (OA)

The first phase of OLS involves constructing an orthogonal array, denoted as $\:{L}_{M}\left({Q}^{d}\right)$, where:$\:M$: Number of experimental combinations (rows in the array),$\:Q$: Number of levels per factor,$\:d$: Number of factors (i.e., problem dimensions).

The number of combinations $\:M$ is determined by:

18 $$\:M=2\cdot\:{\mathrm{l}\mathrm{o}\mathrm{g}}_{2}\left(d+1\right)$$

Each row of the orthogonal array defines a new candidate solution, created by selecting component values from:

- a base vector$$\:{x}_{1}$$,
- a companion vector$$\:{x}_{2}$$,
- or an interpolated vector
  19 $$\:v={x}_{1}+\mathrm{rand}\cdot\:({x}_{2}-{x}_{1})$$

Because all level combinations are equally represented, this construction ensures uniform and balanced sampling of the solution space.

##### *Example*

For a 3-dimensional Sphere function$\:{\mathrm{O}\mathrm{A}(2}^{3})$, an OA might look like:

$$\:\left(\begin{array}{ccccc}\mathrm{Combination}&\:{y}_{1}\left(\mathrm{stage}\right)&\:{y}_{2}\left(\mathrm{stage}\right)&\:{y}_{3}\left(\mathrm{stage}\right)&\:\mathrm{Fitness}\\\:{S}_{1}&\:1\hspace{0.17em}\left(1\right)&\:2\hspace{0.17em}\left(1\right)&\:3\hspace{0.17em}\left(1\right)&\:f\left({S}_{1}\right)=14\\\:{S}_{2}&\:1\hspace{0.17em}\left(1\right)&\:1\hspace{0.17em}\left(2\right)&\:4\hspace{0.17em}\left(2\right)&\:f\left({S}_{2}\right)=18\\\:{S}_{3}&\:2\hspace{0.17em}\left(2\right)&\:2\hspace{0.17em}\left(1\right)&\:4\hspace{0.17em}\left(2\right)&\:f\left({S}_{3}\right)=24\\\:{S}_{4}&\:2\hspace{0.17em}\left(2\right)&\:1\hspace{0.17em}\left(2\right)&\:3\hspace{0.17em}\left(1\right)&\:f\left({S}_{4}\right)=14\\\:&\:&\:&\:&\:\end{array}\right)$$

In addition to producing a varied set of evaluation solutions, this structured sampling removes the need for thorough enumeration.

#### Factor Analysis (FA)

The impact of each level on the different factors is investigated in the second phase, called Factor Analysis (FA), in order to identify the best possible component combination. It is calculated that each level l’s contribution to factor q is equal to:

20 $$\:{W}_{q,l}=\sum\:_{m=1}^{M}f\left({C}_{m}\right)\cdot\:{E}_{m,q,l}$$

where:$\:{W}_{q,l}$: Effect of level $\:l$ on factor $\:q$,$\:f\left({C}_{m}\right)$: Fitness of the $\:m$-th combination,$\:{E}_{m,q,l}$: Indicator variable:

21 $$\:{E}_{m,q,l}=\left\{\begin{array}{ll}1,&\:\mathrm{if\:level\:}l\mathrm{\:is\:used\:for\:factor\:}q\mathrm{\:in\:combination\:}m\\\:0,&\:\mathrm{otherwise}\end{array}\right.$$

The optimal level for each factor is the one that maximizes $\:{W}_{q,l}$. For instance (Table 1):

**Table 1:** Orthogonal based learning applied to the three-dimensional sphere function.

Level	Factor analysis			
L1	f(S1) + f(S2) = 32	f(S1) + f(S3) = 38	f(S1) + f(S4) = 28	
L2	f(S3) + f(S4) = 38	f(S2) + f(S4) = 32	f(S2) + f(S3) = 42	
Best Level	**y1(1)**	**y2(2)**	**y3(1)**	
OL result	**1**	**1**	**3**	$\:f$ₘ_i_ₙ = 11

Significant values are in bold.

#### OLS-based update operator

In the last step, an orthogonal learning operator is applied to update the solution by integrating the results of OA and FA:

22 $$\:{X}_{n}^{m}={X}_{n,\mathrm{best}}^{m}\oplus\:{X}_{n}^{m}$$

where:$\:{X}_{n}^{m}$: Updated solution for the $\:n$-th agent at iteration $\:m$,$\:{X}_{n,\mathrm{best}}^{m}$: Best-performing agent in the current generation,$\:\oplus\:$: Denotes the orthogonal combination of components guided by OA and FA.

By cleverly fusing the best and existing solutions within an orthogonally learned structure, the orthogonal learning operator improves convergence both statistically and directionally, producing new solutions. The following are some significant benefits of combining the FGO algorithm with the Orthogonal Learning Strategy (OLS): Balanced search: Generates a variety of well-organized candidate solutions, Less computation: Uses fewer evaluations to explore the search space effectively, Better convergence: Handles high-performing areas of the solution space more skillfully and Parameter-free integration: Easily incorporates into metaheuristic frameworks and doesn’t require extra hyperparameter tuning. The suggested OLFGO algorithm greatly improves both local and global exploitation capabilities by integrating OLS. Particularly when addressing intricate and multimodal problems like multilevel thresholding in medical image processing, this dual improvement produces more accurate segmentation results and robust optimization performance^5,11,53,54^. The search process is arranged by OLS’s structured learning mechanism to avoid premature convergence to local optima and to hasten the development of high-quality solutions.

## The proposed OLFGO algorithm

To overcome the drawback of the early Fungal Growth Optimizer (FGO) models like premature convergence, limited exploration, and local optima trapping, a better version, the OLFGO, is suggested. The most significant innovation of OLFGO is the inclusion of an OLS, which significantly speeds up local exploitation, solution diversity, and convergence precision. OLFGO is especially well-suited to challenging optimization problems, such as multilevel image thresholding, with highly nonlinear, multimodal, and precision-demanding search space.

### Initialization phase

OLFGO begins by generating an initial population of $\:N$ agents (also called hyphae), where each agent represents a candidate solutiontypically a vector of threshold values. Each agent is initialized within the predefined lower and upper bounds $\:[lb,ub]$ using a uniform distribution:

23 $$\:{S}_{i}^{0}=lb+\mathrm{rand}\left(1,d\right)\cdot\:\left(ub-lb\right)$$

where:$\:d$ is the problem dimensionality (i.e., number of thresholds), $\:\mathrm{rand}(1,d)$ is a vector of random values in $\:[0,1]$.

#### Fitness evaluation phase

We assess each solution with Otsu’s method, which focuses on maximizing the variance between different classes in the segmented areas of the image. The solution yielding the highest fitness value is retained as the global best solution$\:{\:S}^{\mathrm{*}}$for subsequent iterations^17^.

### Fungal growth update phase

Three primary operators make up OLFGO’s core update mechanism, which draws inspiration from the hyphal behaviors of fungi:

1. Tip Growth Behavior: Uses an exponential, energy-driven model to update agents in order to simulate directional hyphal elongation:
  24 $$\:{\overrightarrow{S}}_{i}^{t+1}={\overrightarrow{S}}_{i}^{t}+E\cdot\:\left({\overrightarrow{S}}_{a}^{t}-{\overrightarrow{S}}_{b}^{t}\right)$$
  where
  25 $$\:E=\mathrm{e}\mathrm{x}\mathrm{p}\left(F\right)\:,\:F=\frac{{f}_{i}}{\sum\:_{k=1}^{N}{f}_{k}}$$
  $\:{S}_{a}$ and $\:{S}_{b}$ are randomly selected agents, and $\:F$ is a fitness-derived scaling factor.
2. Lateral Branching: Enhances local exploration near high-quality solutions by introducing directionally biased movements of the best agents.
3. Spore Germination: Produces progeny that aid the population in escaping local optima while preserving diversity, simulating long-distance dispersal.

Boundary checking is done after each step to make sure that solutions within the specified search space are feasible. Lastly, a greedy selection mechanism is used, and if the updated solution improves the previous fitness value, it is kept^53^.

### Orthogonal learning phase

OLFGO uses OLS to improve convergence and solution quality even more. This module creates structured recombination of two parent solutions, $\:{x}_{1}$ and $\:{x}_{2}$, as well as an interpolated vector, using OED:

26 $$\:v={x}_{1}+\mathrm{rand}\cdot\:\left({x}_{2}-{x}_{1}\right)$$

Each row defines an offspring by choosing components from $\:{x}_{1}$, $\:{x}_{2}$, or $\:v$ in an orthogonal array $\:{L}_{M}\left({Q}^{d}\right)$. If the best offspring outperforms the current best solution, it is kept, as measured by its fitness value.

The notation for the recombination is:

27 $$\:{X}_{\mathrm{new}}={x}_{1}\oplus\:{x}_{2}\oplus\:v\:\mathrm{(via\:OA-guided\:selection)}$$

By reducing redundancy and encouraging balanced solution space exploration, this method eventually improves the optimizer’s robustness and efficiency^11,53,55^.

### Termination phase

The OLFGO algorithm iterates until it reaches either the maximum fitness evaluation or the maximum number of iterations $\:{\:T}_{\mathrm{m}\mathrm{a}\mathrm{x}}$. The final output, which represents the ideal threshold set for the specified image segmentation task, is the best solution $\:{S}^{\mathrm{*}}\:$found thus far.

**Algorithm 2:**
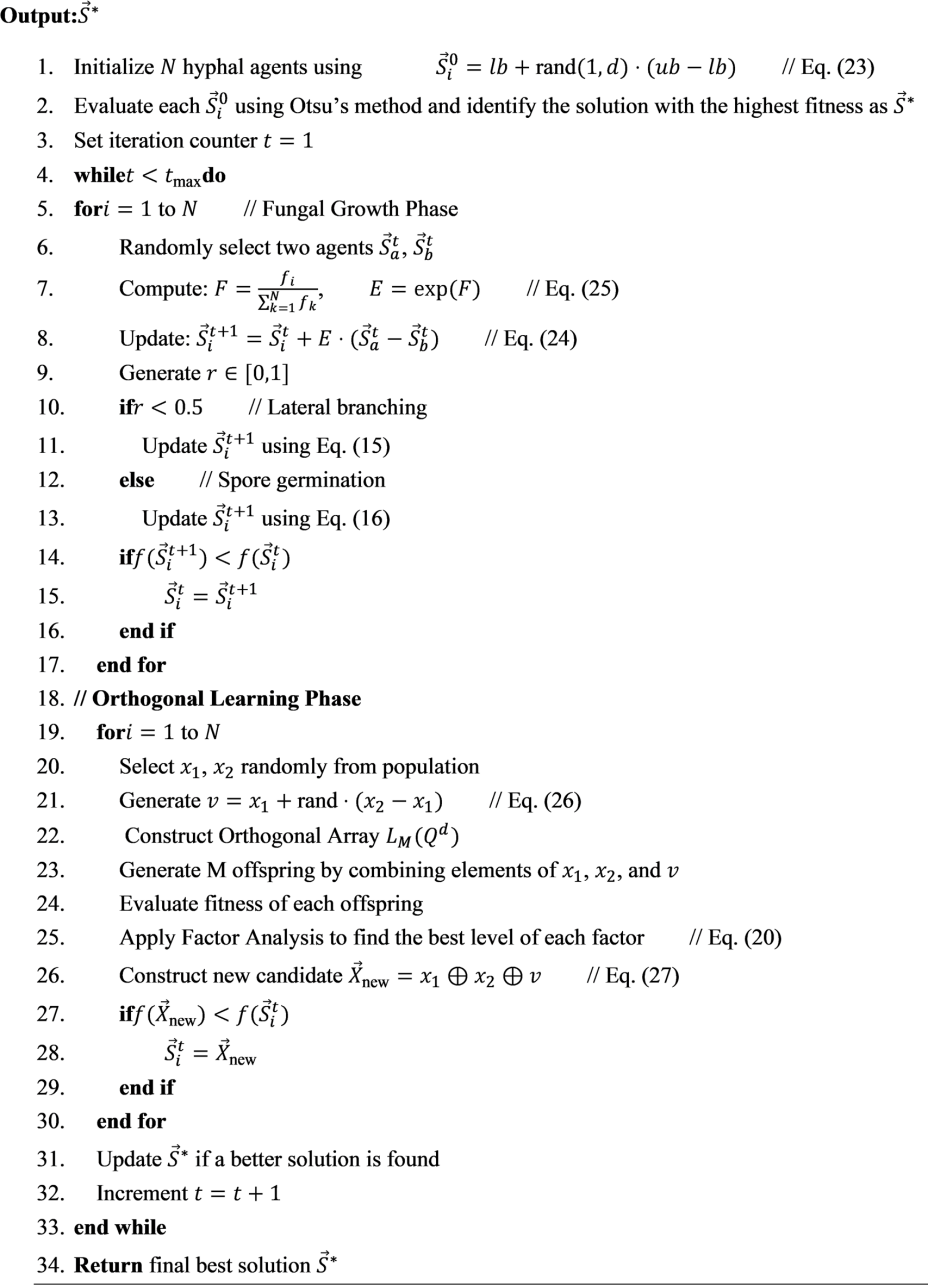
Pseudocode of OLFGO.

### Computational complexity

The computational complexity of OLFGO per iteration includes the following components:

- Fungal Update Operations (tip growth, branching, germination): $\:\mathcal{O}(N\cdot\:d)$
- Orthogonal Learning Evaluations (OA construction and analysis): $\:\mathcal{O}(M\cdot\:d)$, where $\:M=2\cdot\:{\mathrm{l}\mathrm{o}\mathrm{g}}_{2}(d+1)$.
- Fitness Evaluation (Otsu criterion): $\:\mathcal{O}(N\cdot\:\mathrm{l}\mathrm{o}\mathrm{g}d)$
- Total per iteration:

28 $$\:\mathcal{O}\left(\mathrm{OLFGO}\right)=\mathcal{O}\left(\left(N+M\right)\cdot\:d\right)$$

Through the orthogonal array’s compact design and selective local exploitation strategy, OLFGO maintains computational efficiency even after integrating the OLS module, guaranteeing scalability for segmentation and optimization tasks in the real world.

### Differentiation with existing work

The orthogonal learning process allows the algorithm to effectively search for potential areas while ensuring diversity, thus avoiding premature convergence. It is evident that the orthogonal learning strategy (OLS) has been incorporated into various metaheuristic algorithms, such as PSO, DE, and RIME. Nonetheless, the uniqueness of the OLFGO model is not only limited to incorporating the OLS algorithm but also in coupling the OLS algorithm with the special growth behavior of fungi that is not found in any other OLS algorithm. The following distinctions are illustrated in Table 2 as follows:

**Table 2:** Comparison with existing work.

Aspect	OLS-PSO^11^/OLS-DE^12^/OLS-RIME^54^	OLFGO (Proposed)
OLS integration point	Applied globally or during stagnation phases	Embedded within the hyphal growth cycle after natural branching/spore germination
Interaction type	Linear combination of positional vectors	Bioluminescence-inspired guidance: $\:v={x}_{1}+\mathrm{rand}\cdot\:({x}_{2}-{x}_{1})$mimicking nutrient-sensing filament extension
Factor analysis alignment	Generic factor-level optimization	Factor levels correspond to hyphal tip elongation, septation, and anastomosis parameters
Offspring generation basis	Random or fitness proportional selection	Offspring derived from three distinct fungal states: parent hypha (*x*1), neighboring hypha (*x*2), and exploratory germ tube (*v*)
Update acceptance rule	Standard greedy selection	Conditionally integrated with cytoplasmic streaming dynamics update only if $\:f\left({\overrightarrow{X}}_{\mathrm{new}}\right)<f\left({\overrightarrow{S}}_{i}^{t}\right)$ and spatial compatibility holds

## Experimental results and discussion

### Dataset

The proposed OLFGO experimented on two primary datasets:

#### Medical image dataset

**Fig. 1:**
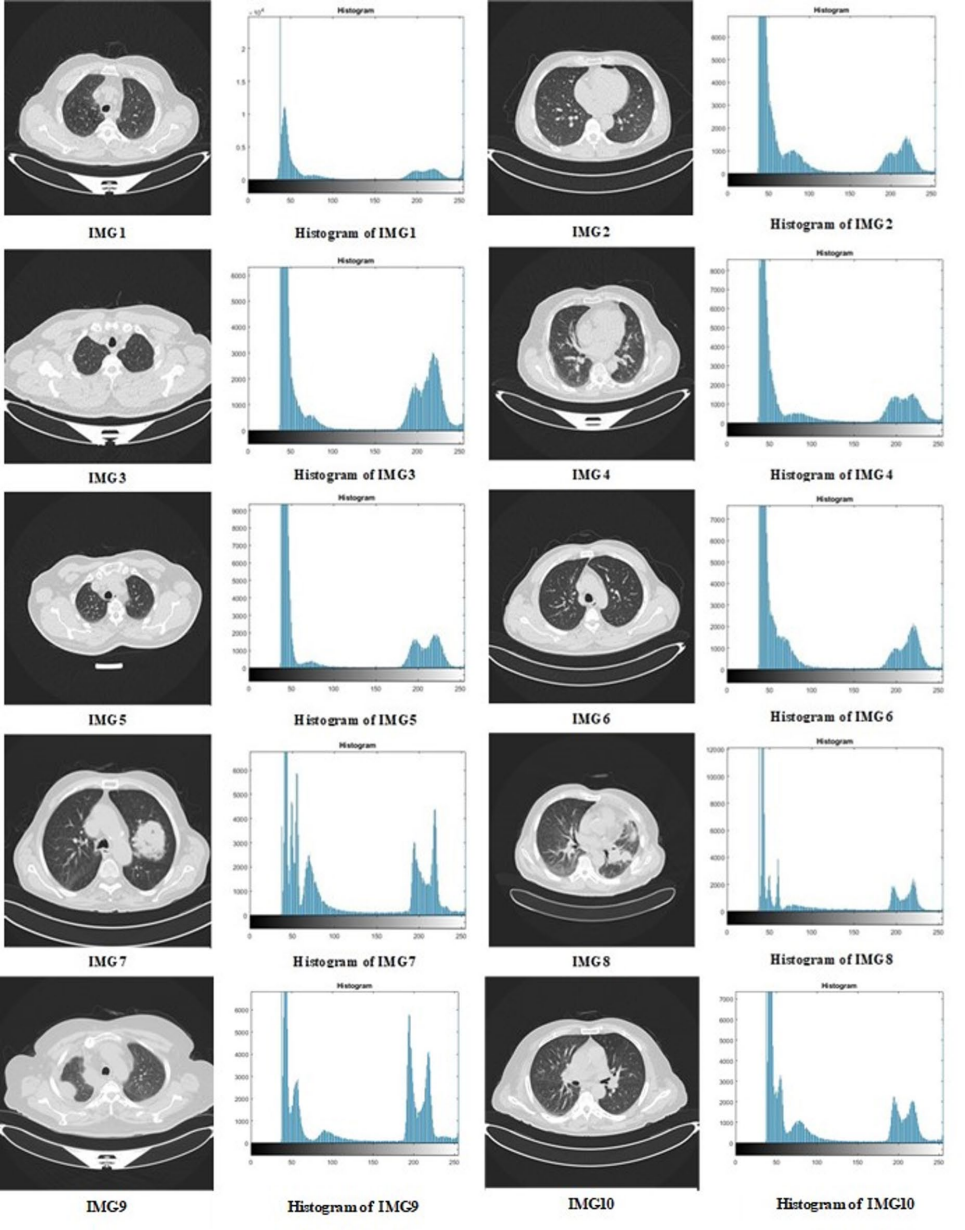
Some of the lung cancer images with their histogram.

The data employed in this study consist of a series of lung cancer images assigned to three types of cases: Benign, Malignant, and Normal^56^. It is made up of CT scans gathered from two medical centers in Iraq: the Iraq-Oncology Teaching Hospital and the National Center for Cancer Diseases. The images not only vary in their label but also in size, making automated analysis more challenging. The most common image size across the dataset is 512 × 512 pixels, with major exceptions of dimensions 512 × 623, 512 × 801, and a few others distant from the mean such as 404 × 511. In the dataset, each patient has a set of CT images, making it appropriate for both image-level and patient-level analysis, although careful patient-wise splitting is necessary to avoid data leaking. No external dataset or cross-dataset validation was used, as the study relies on a single publicly available dataset. To increase generalizability, validation on separate datasets will be a part of future study. In our study we take the 20 chest CT images from a publicly available lung cancer dataset which is depicted in Fig. 1 with their histogram. Balanced sampling strategy is used to ensure equal representation of classes. In order to ensure a thorough assessment of segmentation performance, the images were selected to capture a range of complexity and intensity.

For evaluating how well our method segments lungs we got the masks from a lung segmentation dataset that is available to everyone. We used these masks as the standard to compare with to calculate scores, like the Dice Similarity Coefficient and other metrics that tell us how good our segmentation is. The lung segmentation dataset has masks that are already known to be correct. These masks go with CT images of lungs. This helps us to accurately check how well our OLFGO-based method works for segmenting lungs.

OLFGO is tested based on the use of lung CT images extracted from LungSegDB dataset, which is a publicly available lung segmentation dataset accessible from the following link: https://github.com/sadjadrz/Lung-segmentation-dataset. LungSegDB database was created following the methodology comprising dataset collection, manual lung mask creation, expert verification, and annotation validation process. In the dataset, users can find images of CT scans together with segmented masks of lungs presented in the form of raw and preprocessed data.

#### Benchmark functions for CEC 2022

To evaluate the algorithm’s resilience to various problem topologies, including unimodal, multimodal, and hybrid components, a set of 12 benchmark functions (F1–F12) was used^16^.

Additionally, to guarantee consistency and dependability in segmentation evaluation, chest X-ray images were preprocessed by removing artifacts and scaling intensity values.

### Evaluation criteria

For a comparison of the performance of the suggested OLFGO for medical image segmentation, several standard quantitative measures were adopted. The measures are compared with segmented image quality and original images visually and structurally. The following parameters were previously used to evaluate the OLFGO algorithm for medical image segmentation:

#### Peak Signal-to-Noise Ratio (PSNR)

By examining individual pixels, PSNR calculates the degree of similarity between the original and processed images. This is the definition of the Mean Squared Error (MSE), which serves as its foundation:

29 $$\:\mathrm{MSE}=\frac{1}{mn}\sum\:_{i=0}^{m-1}\sum\:_{j=0}^{n-1}{\left[I(i,j)-K(i,j)\right]}^{2}$$

30 $$\:\mathrm{PSNR}=10\cdot\:{\mathrm{l}\mathrm{o}\mathrm{g}}_{10}\left(\frac{MA{X}_{I}^{2}}{\mathrm{MSE}}\right)$$

where:$\:I(i,j)$ and $\:K(i,j)$ denote the pixel intensities of the original and segmented images at position $\:(i,j)$, respectively.$\:m$ and $\:n$ are the image dimensions.$\:MA{X}_{I}$ is the maximum possible pixel value (e.g., 255 for 8-bit images).

A higher PSNR value indicates closer similarity and better segmentation quality.

#### Structural Similarity Index (SSIM)

By examining elements like brightness, contrast, and overall structure, SSIM determines how similar two images are. This is the breakdown of the calculations:

31 $$\:\mathrm{SSIM}\left(x,y\right)=\frac{\left(2{\mu\:}_{x}{\mu\:}_{y}+{C}_{1}\right)\left(2{\sigma\:}_{xy}+{C}_{2}\right)}{\left({\mu\:}_{x}^{2}+{\mu\:}_{y}^{2}+{C}_{1}\right)\left({\sigma\:}_{x}^{2}+{\sigma\:}_{y}^{2}+{C}_{2}\right)}$$

where:$\:{\mu\:}_{x}$, $\:{\mu\:}_{y}$ are the mean intensities,$\:{\sigma\:}_{x}^{2}$, $\:{\sigma\:}_{y}^{2}$ are the variances, and$\:{\sigma\:}_{xy}$ is the covariance between corresponding local regions of the original and segmented images.$\:{C}_{1}$, $\:{C}_{2}$ are small constants to stabilize the division.

SSIM values range from 0 to 1, where a value closer to 1 indicates high structural similarity.

#### Feature Similarity Index (FSIM)

FSIM uses low-level feature components, specifically phase congruency (PC) and gradient magnitude (GM), to assess the perceptual similarity between images. Its definition is as follows:

32 $$\:\mathrm{FSIM}\left({I}_{r},{I}_{d}\right)=\frac{\sum\:_{x\in\:{\Omega\:}}{\mathrm{PC}}_{m}\left(x\right)\cdot\:{S}_{L}\left(x\right)}{\sum\:_{x\in\:{\Omega\:}}{\mathrm{PC}}_{m}\left(x\right)}$$

where:$\:{\mathrm{PC}}_{m}\left(x\right)=\mathrm{m}\mathrm{a}\mathrm{x}\left({\mathrm{PC}}_{r}\left(x\right),{\mathrm{PC}}_{d}\left(x\right)\right),\:{S}_{L}\left(x\right)={S}_{PC}\left(x\right)\cdot\:{S}_{GM}\left(x\right)$ denotes local similarity between reference and distorted images.FSIM ranges from 0 to 1, with higher values indicating better feature preservation^14^.

#### Universal Quality Index (UQI)

By simultaneously examining brightness, contrast, and structural similarities between two images, UQI quantifies the distortion of images. This is the formula for it:

33 $$\:\mathrm{UQI}\left(x,y\right)=\frac{4{\mu\:}_{x}{\mu\:}_{y}{\sigma\:}_{xy}}{\left({\mu\:}_{x}^{2}+{\mu\:}_{y}^{2}\right)\left({\sigma\:}_{x}^{2}+{\sigma\:}_{y}^{2}\right)}$$

where all terms carry the same definitions as in SSIM. A UQI value close to 1 implies a high degree of similarity.

#### Dice Coefficient (DC)

Dice’s Coefficient is one of the most commonly used techniques in the domain of medical image segmentation. The value of the Dice coefficient lies between 0 and 1, and the higher the value of the Dice coefficient, the more similar the two regions are^15^.

The mathematical expression for the Dice coefficient is given as:

34 $$\:\mathrm{D}\mathrm{i}\mathrm{c}\mathrm{e}=\:\frac{2TP}{2TP+FP+FN}$$

where TP is the number of pixels that have been rightly labeled as being part of the target region, FP is the number of pixels that have wrongly been labeled as being part of the target region, and FN refers to the number of pixels belonging to the target region but have been wrongly labeled as being background pixels.

#### Friedman Rank Test

One statistical method that doesn’t require a particular data distribution is the Friedman test. It’s useful for comparing algorithms on different datasets. Without requiring a normal distribution, it essentially examines whether the variations in how these algorithms rank do matter statistically. Thus, it’s a good choice for experimental research algorithm comparisons.

#### According to CEC 2022 Benchmark Functions

Mean Fitness and Standard Deviation (Std), two commonly used statistical measures, were used to assess the optimization efficiency of the suggested OLFGO algorithm on the CEC 2022 benchmark test suite. These metrics shed light on the algorithm’s overall performance as well as its dependability over several separate runs.

##### Mean Fitness (Mean)

Average fitness value is the average solution quality achieved after some independent runs (typically 30). It indicates the algorithm’s ability to converge to the global optimum. It is calculated as:

35 $$\:\mathrm{Mean}=\frac{1}{N}\sum\:_{i=1}^{N}f\left({x}_{i}\right)$$

where:$\:f\left({x}_{i}\right)$: fitness value obtained in the $\:{i}^{th}$ run,$\:N$: total number of independent runs (e.g., $\:N=30$).

In general, better optimization performance on minimization problems is reflected in a lower mean fitness value^16^.

##### Standard Deviation (Std)

The standard deviation gives us insight into the algorithm’s stability and dependability over time. Larger values indicate algorithm performance variability, whereas smaller values indicate more consistent results. The formula for calculating the standard deviation is:

36 $$\:\mathrm{Std}=\sqrt{\frac{1}{N}\sum\:_{i=1}^{N}{\left(f\left({x}_{i}\right)-\mathrm{Mean}\right)}^{2}}$$

where the definitions of the terms are the same as those given above.

Because they provide useful information about the exploration and exploitation capabilities of optimization algorithms, these two indicators are frequently used in comparative studies on CEC benchmark problems^16,57^.

### Results and discussion

#### Medical image segmentation

**Fig. 2:**
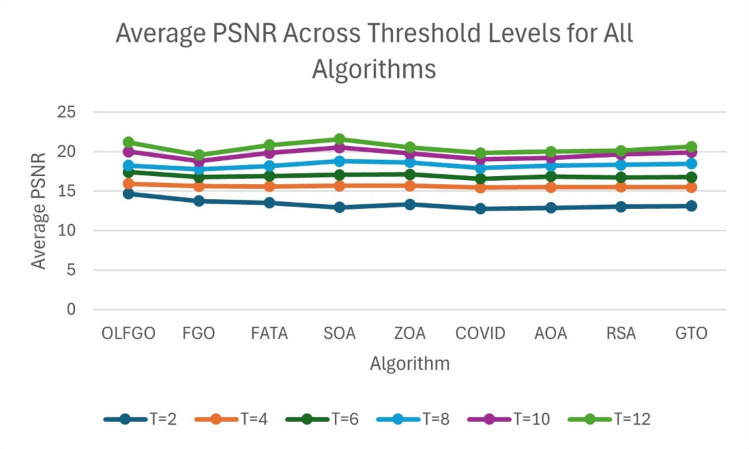
Comparison interms of average PSNR.

**Fig. 3:**
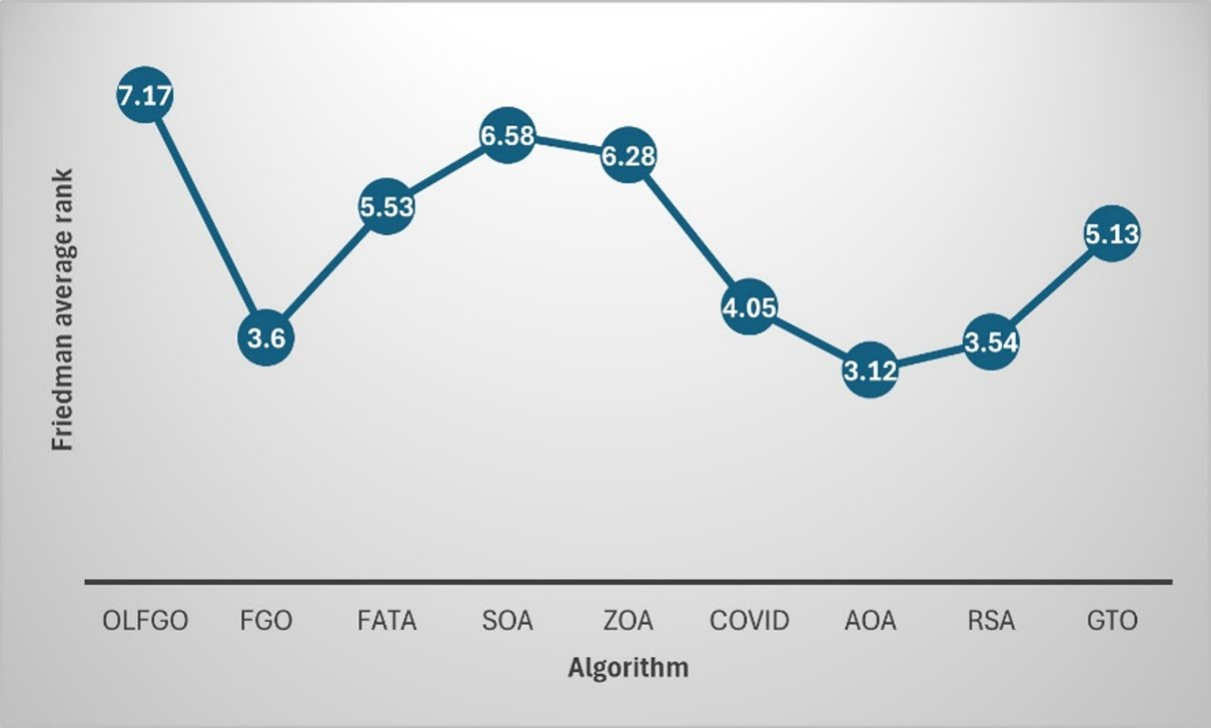
Comparison in term of Friedman average rank.

A set of 20 chest CT images of lung cancer were segmented with the help of multilevel thresholding on six levels: 2, 4, 6, 8, 10, and 12 and performed independently 30 times. Eight state-of-the-art algorithms, including FGO, FATA, SOA, ZOA, COVID, AOA, RSA, and GTO, were compared with the proposed algorithm. The parameter settings for all algorithms are described in Table 3. Four established image quality measures were used: PSNR, SSIM, FSIM, UQI and DICE. You can find the summarized results in Tables 4, 5, 6, 7 and 8 and see the visuals in the corresponding figures (Figs. 2 and 3).

**Table 3:** Parameter settings.

Algorithm	Parameter	Value
Common parameters	Population size	50
	Maximum number of iterations	100
	Dimension	Number of thresholds
	Number of trials	30
FGO	Growth rate (G)	[0.5–1.0]
	Branching probability	0.3
	Exploration & exploitation factor	0.5
	step size	0.1
FATA	Exploration & exploitation factor	0.5
	Randomization probability (Pr)	[0.2–0.3]
	Adaptation rate	[0.1–0.3]
	step size	0.1
SOA	Collision factor	Random number in [0, 1]
	Spiral attack constants (u, v)	1
ZOA	Foraging coefficient (r₁) and Defense coefficient (r₂)	Random number in [0, 1]
	Attack factor (β)	[0.5–1]
	Awareness probability (P)	0.5
COVID	Shifting number	1
	Number of sub proteins	2
	MR	0.5
AOA	Transition from exploration to exploitation	[0.2-1]
	α (alpha)	5
RSA	α (alpha)	5
	r_1_, r_2_, and r_3_	Random values in [0, 1]
GTO	Exploration probability (p)	0.5
Proposed OLFGO	Number of levels per factor (Q)	3
	Number of combinations (M)	(Q^d − 1)/(Q − 1)
	Number of factors (d)	ceil(log(dim)/log(Q))
	Growth rate (G)	[0.5–1.0]
	Branching probability	0.3
	Exploration & exploitation factor	0.5
	step size	0.1

**Table 4:** Comparison in terms of PSNR metric and its Friedman average rank.

Image	Threshold	Algorithm								
		OLFGO	FGO	FATA	SOA	ZOA	COVID	AOA	RSA	GTO
IMG1	2	**14.5325**	12.5909	13.7731	12.5551	13.4584	12.5437	12.7527	13.7561	13.7701
	4	**15.6847**	15.5394	15.5512	15.5095	15.5275	15.5259	15.5074	15.4758	15.4418
	6	**17.2473**	17.1043	16.7653	16.8968	17.0661	16.574	16.9118	16.8146	16.7627
	8	17.8414	17.5913	18.5597	18.9389	18.7847	**18.9785**	18.4012	18.7806	18.3968
	10	**20.981**	18.9889	20.5927	20.5775	19.8023	19.6943	18.5887	20.1348	20.3012
	12	21.2275	19.4056	**21.7814**	21.6949	20.9732	19.4342	19.2885	19.9063	21.1491
IMG2	2	**14.0049**	13.0628	13.1315	12.3935	13.144	12.4644	12.6125	13.3151	13.7136
	4	**15.8297**	15.6278	15.6688	15.7287	15.7492	15.6602	15.6922	15.5872	15.748
	6	16.4494	16.3153	**17.4196**	17.3091	17.3734	17.0846	16.9623	17.0384	17.3688
	8	**19.1678**	17.6943	18.0703	18.5071	18.1885	17.8483	18.0307	17.934	18.0488
	10	**21.6603**	18.5463	19.1555	20.9419	19.4237	20.8753	18.1805	18.7686	19.5604
	12	20.9558	18.1105	20.6462	**22.1574**	20.672	18.8059	18.8667	20.8948	20.6147
IMG3	2	**14.9228**	14.457	12.6295	12.2693	13.4408	13.0425	12.4713	11.5474	11.5474
	4	**15.7379**	15.4822	15.5851	15.505	15.5453	15.3972	15.5226	15.5673	15.5372
	6	**17.3192**	17.1574	17.282	17.2925	17.2658	17.2414	17.2949	17.1894	17.2751
	8	18.0884	17.9046	18.378	18.9476	18.5878	**18.9544**	17.8068	18.5035	18.346
	10	18.1269	20.4536	19.558	20.8665	19.5883	**20.9456**	18.7136	18.0296	19.3285
	12	**23.8574**	19.227	19.8907	22.1812	20.6208	18.5977	18.6143	19.0764	21.131
IMG4	2	**14.0268**	13.4074	13.5256	12.4753	13.2456	12.306	12.6389	12.1022	13.165
	4	**15.7024**	15.2487	15.6767	15.6548	15.6445	15.6126	15.6251	15.5974	15.6792
	6	**17.0057**	16.8443	16.6433	16.9522	16.7856	16.8258	16.6014	16.9365	16.6246
	8	**19.6584**	18.0238	18.5721	18.9329	18.6865	17.8144	18.3973	17.2602	18.6015
	10	**20.6838**	19.8158	20.0833	20.5631	19.8994	19.674	18.8677	19.0014	20.0212
	12	20.5205	19.9674	20.0507	**21.518**	21.032	19.3284	19.4532	20.5991	20.8223
IMG5	2	**15.453**	14.7476	12.8625	12.9474	14.0451	13.9965	13.0413	15.4464	12.6337
	4	**16.2664**	15.6143	15.8172	15.7973	15.8072	15.8691	15.7853	15.7639	15.8148
	6	**16.9562**	16.591	16.6215	16.6171	16.6389	16.4938	16.5398	16.3201	16.6373
	8	19.191	19.1079	**19.6808**	19.2438	18.7932	17.7185	17.2912	16.9059	18.9528
	10	17.383	18.5069	19.5919	**20.7485**	19.4589	19.6646	17.6219	19.0055	20.0672
	12	19.9359	19.3575	20.9854	**21.4962**	20.6708	19.7918	18.0808	19.9762	20.9501
IMG6	2	**14.0335**	12.5683	13.0269	12.7397	13.4403	13.2215	12.7266	12.4306	12.4306
	4	**16.2651**	16.1906	16.0515	16.037	16.1231	16.192	16.0373	16.1892	16.0388
	6	**17.8668**	17.0486	17.0074	17.2074	17.3551	17.416	17.0771	17.044	16.9724
	8	17.6943	17.7427	18.5442	**18.8285**	18.3838	17.9135	17.8638	17.5138	18.3073
	10	19.6567	18.8377	20.0365	**20.8853**	19.882	18.8251	18.4311	18.0974	20.1311
	12	19.8848	18.7738	21.129	**22.2306**	21.0348	21.3064	19.0213	18.9025	21.0413
IMG7	2	**13.3293**	11.9429	11.737	12.4592	12.9178	11.9823	12.1692	13.0678	12.8835
	4	**16.7514**	15.5072	15.8506	15.8368	16.3466	15.8497	15.8712	16.107	15.8472
	6	**17.6298**	17.1841	17.0527	17.1694	17.277	16.8245	17.1209	17.5359	17.0394
	8	17.9808	17.4339	19.4142	**19.4275**	19.3689	18.436	18.2516	17.2461	19.3727
	10	**20.705**	19.0433	20.017	20.4886	19.9878	19.4998	18.5422	19.2443	19.9901
	12	20.4129	19.9534	20.3111	**22.6073**	20.2328	20.1654	19.7808	19.4702	20.1455
IMG8	2	**14.5804**	13.8335	13.5347	13.1533	13.8134	13.0074	12.9787	12.5465	12.5465
	4	16.3568	16.2854	15.9885	16.2649	**16.3796**	16.1871	15.9646	16.0945	16.2784
	6	**17.2437**	17.0542	17.2055	17.2318	17.2242	17.0837	16.8693	16.9368	17.1978
	8	17.5212	17.474	17.6791	18.1773	17.7139	17.4183	17.6446	**18.3074**	17.665
	10	17.4883	17.9403	17.9562	20.8644	18.2096	17.7433	18.1837	19.1924	**21.5953**
	12	21.0889	20.8565	19.8451	21.52	19.0586	21.7801	18.219	17.7916	**22.2891**
IMG9	2	**13.6659**	12.3099	11.6022	12.0683	13.4651	12.3622	12.0702	11.6022	11.6022
	4	**15.9841**	15.6907	15.7494	15.8507	15.8532	15.8511	15.7998	15.4964	15.8767
	6	17.1316	17.1183	17.0055	**17.1886**	17.1193	16.675	16.9182	16.8605	16.9909
	8	**19.2044**	18.4256	19.0593	18.9953	18.8948	18.5855	18.1582	18.437	18.9713
	10	19.3188	18.8608	19.4279	**20.9206**	19.6853	20.2664	18.844	19.3611	19.2886
	12	**24.9282**	19.1935	19.3343	22.1696	19.7839	19.226	19.4718	19.2589	21.9842
IMG10	2	**14.1925**	13.4901	12.2889	12.5909	13.096	12.6385	12.3839	13.7355	12.5069
	4	**15.7684**	15.5144	15.5298	15.6338	15.6225	15.4526	15.5283	15.492	15.6193
	6	**17.2201**	16.6669	16.6656	16.8161	16.831	16.52	16.6276	16.5391	16.6538
	8	17.4471	17.6733	18.2679	**18.3527**	18.2557	17.6271	17.8199	17.7499	18.2504
	10	19.4103	18.3093	18.565	**20.5311**	18.8895	18.4673	18.4577	18.24	18.4999
	12	18.9777	19.7079	20.2608	**22.2601**	20.3733	18.5649	18.893	18.0889	22.1608
Friedman average rank		7.17	3.6	5.53	6.58	6.28	4.05	3.12	3.54	5.13
Rank		1	7	4	2	3	6	9	8	5
IMG11	2	13.5132	13.328	13.2737	**13.9889**	13.3297	13.3983	13.8713	13.5959	13.3739
	4	**19.2017**	16.7765	14.6687	17.8043	14.52	14.8397	15.9366	16.9057	14.6445
	6	**19.3847**	17.4732	18.2952	18.8077	16.645	16.815	15.9658	17.0215	16.3323
	8	**19.4562**	18.6072	18.8362	19.4059	17.7694	17.8735	15.7936	17.801	16.9834
	10	19.5204	18.5275	19.6999	**22.8169**	17.8966	18.0443	16.5759	17.8896	18.4677
	12	19.5102	18.7441	19.5831	**24.8574**	18.3143	18.8471	16.5859	18.4126	18.6922
IMG12	2	13.4628	13.2423	13.2528	13.8998	13.2789	13.3255	**13.9201**	13.5242	13.4664
	4	**18.6501**	16.5153	14.7919	17.6237	14.5308	14.9718	15.8156	16.6378	14.6411
	6	**18.7631**	17.8004	18.4089	18.6042	16.8305	15.9553	16.3378	16.9511	16.2879
	8	**18.7859**	18.2027	17.865	18.3605	17.1527	16.2646	16.5629	17.3344	16.9579
	10	18.8453	18.4136	19.7953	**20.9538**	17.775	17.9027	16.3552	17.8502	20.7324
	12	18.2237	18.5326	19.2236	**24.353**	18.4123	17.3484	23.1411	18.0338	18.0714
IMG13	2	13.7016	13.5276	13.6401	**14.0669**	13.6052	13.6685	13.9675	13.7945	13.7032
	4	**17.9864**	17.7063	14.8861	17.5997	14.9541	14.519	15.7703	16.8788	14.9361
	6	19.7838	18.6268	18.5693	**19.8447**	17.5368	18.9248	16.1551	18.0466	16.9269
	8	**19.8906**	18.6738	18.4839	19.5528	18.1867	17.5523	16.7936	18.2504	18.7166
	10	19.9741	19.5774	20.1718	**20.4724**	18.5522	17.3531	16.9981	18.7693	19.1341
	12	20.0162	19.6936	19.6679	**21.9987**	18.6965	18.6324	17.9877	19.2173	19.1321
IMG14	2	13.1532	12.9875	12.9274	13.8198	12.9908	13.0223	**13.9**	13.3269	13.1834
	4	**18.0627**	16.7142	14.194	17.6309	14.1356	14.1811	16.0408	15.5475	14.3829
	6	18.1066	17.2151	18.335	**21.7444**	16.5134	16.6561	15.5789	16.0572	15.9823
	8	18.1756	17.8154	17.3379	**20.0742**	16.9188	16.847	16.8574	16.8189	17.5022
	10	18.1874	17.7008	18.9548	**20.0816**	17.3062	17.6203	17.7616	17.2452	17.3946
	12	18.2523	17.7373	21.2429	**21.3248**	17.2097	16.9966	17.421	16.7148	17.6413
IMG15	2	**13.6523**	13.3636	13.3081	13.2207	13.3664	13.419	13.1574	13.6445	13.5601
	4	**18.2856**	16.8811	14.7541	18.1039	14.4087	14.2389	16.1121	16.2933	14.7216
	6	**18.395**	17.341	17.509	17.5804	16.5537	17.0675	15.7809	17.017	15.9832
	8	**18.4695**	17.7995	18.0267	17.4779	16.9026	15.8715	15.6194	17.1707	17.3032
	10	18.6109	17.8395	18.6685	**19.2467**	17.612	17.2186	16.5726	17.5323	18.6317
	12	18.583	18.0527	18.2054	**20.8757**	17.6492	18.5669	17.6419	17.5749	18.2787
IMG16	2	**13.7823**	13.5612	13.5227	13.4577	13.5935	13.4686	13.4406	13.724	13.6259
	4	**18.4395**	18.0716	14.9328	17.1659	14.8718	14.7102	15.8785	17.4525	14.8245
	6	**20.6912**	18.7666	18.1816	18.9381	17.3644	16.8057	18.9634	17.4146	17.9603
	8	**20.653**	19.7615	19.3983	19.9102	18.8561	18.9086	19.6	19.1201	17.7406
	10	20.7051	19.3931	20.4457	**20.9185**	18.8166	19.8039	19.4585	19.2268	20.741
	12	20.7302	19.9417	20.8415	**22.7578**	19.4126	20.0277	20.0791	19.0431	20.2552
IMG17	2	**14.5836**	14.3359	14.3015	13.9709	14.3533	14.5113	14.0558	14.4863	14.4483
	4	**20.9621**	18.033	19.0734	17.3374	17.0008	16.92	20.642	18.9743	17.5992
	6	**21.4492**	19.7377	20.0065	19.2282	18.7251	18.2983	21.0997	19.0384	18.2796
	8	21.3002	20.166	21.5751	**22.9439**	19.7085	20.2378	22.577	19.6853	19.5612
	10	21.4988	20.7757	22.5724	22.1446	19.5715	20.2254	**22.958**	19.5264	20.591
	12	**21.4669**	20.8092	21.2972	20.9897	19.8269	19.0697	20.908	19.8264	20.238
IMG18	2	**13.952**	13.6096	13.5485	13.4835	13.6095	13.6107	13.808	13.7703	13.712
	4	**20.4802**	17.5416	15.1122	18.9761	14.929	15.3573	16.2921	17.244	15.095
	6	19.415	19.3781	18.8692	**19.451**	17.5145	17.3519	16.1513	18.1761	17.3944
	8	**20.7118**	19.4388	18.9681	20.6799	18.7215	17.5892	17.137	18.5403	18.3334
	10	**20.8583**	20.0205	20.2647	20.447	18.8764	19.1965	20.3065	18.5405	20.0085
	12	**20.8592**	19.8949	19.7835	20.1546	19.4264	19.1449	20.5756	19.2788	19.669
IMG19	2	**15.096**	14.635	14.5714	15.0356	14.7469	14.8347	14.9136	14.7461	14.7273
	4	**21.2736**	17.8312	16.7929	20.7988	16.4682	15.8397	17.5239	18.9993	16.3558
	6	**21.7023**	19.9312	20.0696	19.9885	18.8309	18.069	18.2034	19.3751	18.5179
	8	**22.003**	20.8595	21.0155	20.668	20.5244	21.3953	20.7919	20.3784	20.3341
	10	**22.2604**	21.3565	21.3917	21.5687	20.8826	21.7014	21.2684	20.0325	20.8949
	12	22.4491	21.5712	**22.8844**	22.7609	20.9577	21.6224	22.3964	21.1018	21.51
IMG20	2	12.5186	12.3824	12.4666	12.2827	12.4178	12.2646	12.2465	12.9624	**12.9734**
	4	**15.5128**	14.4983	13.8556	14.99	13.2344	13.2993	13.2665	14.5495	13.2626
	6	15.6496	15.0167	15.2523	**16.4172**	14.53	13.6391	16.4112	14.993	15.0051
	8	**15.7044**	15.2261	15.2208	15.4598	14.9645	15.1647	15.3644	14.8533	14.3828
	10	15.7668	15.4674	15.668	**15.9024**	15.0269	14.6259	15.5632	14.9104	15.5252
	12	15.7854	15.3561	16.6914	**16.7054**	15.2001	15.1576	16.4005	15.2537	15.9721
Friedman average rank		**7.93**	5.28	5.58	7.47	2.92	3.32	4.38	4.15	3.97
Rank		**1**	4	3	2	9	8	5	6	7

Bold value represents the best finding.

PSNR values always suggest the superiority of OLFGO over all other algorithms for most thresholds and images. For instance, in IMG1, PSNR has improved from 14.53 (threshold 2) to 21.23 dB (threshold 12) for OLFGO, which is far ahead of FGO and others. IMG9 and IMG10 reflect OLFGO’s high segmentation power at higher thresholds with peaks of 24.93 dB and 22.98 dB, respectively. The Friedman average rank confirms OLFGO’s superiority with the first rank followed by SOA and ZOA.

Better reconstruction and segmentation quality are typically indicated by higher PSNR values. OLFGO exhibits superior accuracy, particularly at higher thresholds, suggesting strong edge preservation and noise immunity. Better and more consistent performance is indicated by a higher average rank. OLFGO has the highest average rank, meaning that it consistently performed the best across all images.

**Table 5:** Comparison in terms of SSIM metric.

Image	Threshold	Algorithm								
		OLFGO	FGO	FATA	SOA	ZOA	COVID	AOA	RSA	GTO
IMG1	2	**0.1924**	0.1736	0.1794	0.16771	0.1779	0.16706	0.1705	0.1792	0.1799
	4	0.2566	**0.2583**	0.251	0.25068	0.2514	0.2524	0.2496	0.2564	0.2471
	6	**0.3535**	0.3469	0.3259	0.33498	0.3463	0.31477	0.3316	0.3274	0.3257
	8	0.3907	0.3819	0.4461	0.46544	0.4591	**0.46705**	0.421	0.4504	0.4369
	10	**0.5436**	0.465	0.5398	0.53836	0.5136	0.5	0.4391	0.5238	0.5348
	12	0.5584	0.5	0.5853	**0.59206**	0.5672	0.49288	0.4796	0.5188	0.5703
IMG2	2	**0.1603**	0.1479	0.1415	0.13993	0.1487	0.14017	0.1421	0.1508	0.1556
	4	**0.2461**	0.2328	0.2294	0.23412	0.2357	0.2359	0.2323	0.228	0.2367
	6	0.293	0.2807	**0.3463**	0.34229	0.3456	0.33342	0.3229	0.3273	0.3444
	8	**0.4584**	0.3765	0.406	0.43055	0.4135	0.38509	0.3901	0.3841	0.4043
	10	**0.597**	0.4232	0.481	0.54285	0.4842	0.5305	0.4039	0.4461	0.4993
	12	0.5288	0.4085	0.5376	**0.59203**	0.5361	0.4467	0.443	0.5169	0.5415
IMG3	2	**0.2554**	0.2511	0.2349	0.22824	0.2379	0.23886	0.2279	0.2223	0.2223
	4	**0.2849**	0.2833	0.2829	0.27704	0.2792	0.27679	0.2775	0.2826	0.2779
	6	0.3685	0.373	**0.3756**	0.37536	0.3744	0.37017	0.3752	0.3722	0.3752
	8	0.4365	0.4162	0.464	0.47887	0.468	**0.47913**	0.4148	0.4563	0.4612
	10	0.4419	0.5415	0.5264	**0.56759**	0.5285	0.5602	0.4685	0.4341	0.5241
	12	**0.7235**	0.5152	0.547	0.61066	0.5688	0.48133	0.462	0.4819	0.5921
IMG4	2	**0.1885**	0.171	0.1719	0.16071	0.1713	0.15788	0.1641	0.1554	0.1754
	4	**0.2521**	0.2388	0.247	0.24815	0.2473	0.2458	0.2462	0.2504	0.2505
	6	**0.3301**	0.3135	0.309	0.32941	0.3188	0.32282	0.302	0.324	0.3074
	8	**0.4705**	0.4035	0.436	0.45429	0.443	0.39108	0.4095	0.3544	0.4385
	10	**0.5298**	0.4867	0.5075	0.52591	0.5018	0.48106	0.4437	0.4477	0.5091
	12	0.556	0.4945	0.5201	**0.57559**	0.5516	0.48256	0.4728	0.5226	0.5437
IMG5	2	0.1558	0.1631	0.1482	0.14787	0.1548	0.1574	0.1482	**0.164**	0.146
	4	**0.2203**	0.1948	0.1999	0.19884	0.1999	0.20456	0.1952	0.2029	0.1988
	6	**0.2729**	0.2379	0.2457	0.2457	0.2485	0.24121	0.2399	0.2295	0.2466
	8	0.3741	0.3715	**0.4113**	0.39371	0.3705	0.31848	0.2943	0.2629	0.3899
	10	0.3251	0.3658	0.4198	**0.47544**	0.4059	0.41782	0.3147	0.3767	0.4325
	12	0.4415	0.4178	0.4823	**0.517**	0.4622	0.4201	0.3407	0.4273	0.4676
IMG6	2	**0.1672**	0.1564	0.1622	0.15851	0.1647	0.14408	0.1583	0.155	0.155
	4	**0.277**	0.27	0.2596	0.25596	0.262	0.26439	0.2559	0.2575	0.2536
	6	**0.3821**	0.3234	0.3209	0.33319	0.341	0.34493	0.3238	0.344	0.3195
	8	0.3662	0.3729	0.4328	**0.4437**	0.4211	0.38716	0.3764	0.3602	0.4174
	10	0.4676	0.4529	0.5103	**0.53341**	0.5022	0.44816	0.4131	0.3998	0.516
	12	0.5032	0.4502	0.5507	**0.60206**	0.5464	0.54533	0.4474	0.4492	0.5517
IMG7	2	0.2429	0.2513	0.248	0.25517	0.2603	0.25726	0.2535	**0.2693**	0.2664
	4	**0.4265**	0.3531	0.3758	0.36975	0.3978	0.37755	0.3796	0.3738	0.3754
	6	**0.4603**	0.4396	0.4367	0.44235	0.4466	0.4118	0.4352	0.4502	0.4352
	8	0.4816	0.4415	0.5742	**0.58281**	0.5765	0.52642	0.5048	0.4452	0.5657
	10	0.6197	0.556	0.6141	**0.62841**	0.6126	0.58597	0.519	0.5844	0.5997
	12	0.6255	0.6021	0.6364	**0.70109**	0.6266	0.6074	0.5793	0.5693	0.6132
IMG8	2	**0.1939**	0.1894	0.185	0.17638	0.1778	0.17668	0.1728	0.1703	0.1703
	4	**0.2645**	0.2482	0.2523	0.25768	0.2596	0.26312	0.2443	0.2608	0.2538
	6	**0.3122**	0.3031	0.3091	0.31145	0.3097	0.30165	0.2886	0.3099	0.3052
	8	0.3374	0.3294	0.3465	0.39605	0.3511	0.3289	0.3441	**0.4117**	0.343
	10	0.3373	0.3675	0.3716	0.60088	0.3919	0.35462	0.3818	0.4769	**0.6495**
	12	0.5364	0.6122	0.5226	0.63965	0.4591	0.66417	0.3803	0.3442	**0.6906**
IMG9	2	0.2958	0.3183	0.3146	0.31888	**0.3287**	0.31924	0.3156	0.3146	0.3146
	4	**0.3831**	0.3669	0.3619	0.36769	0.3693	0.36922	0.3641	0.3723	0.3735
	6	0.4322	0.4305	0.4232	**0.44026**	0.4336	0.40747	0.4205	0.4372	0.4232
	8	**0.5403**	0.4756	0.5323	0.53036	0.5219	0.50355	0.474	0.4786	0.523
	10	0.5447	0.5294	0.5447	**0.61154**	0.5652	0.58621	0.5101	0.5218	0.5372
	12	**0.7865**	0.5147	0.5363	0.66145	0.5644	0.53158	0.5447	0.5326	0.6353
IMG10	2	**0.2242**	0.1847	0.2002	0.20119	0.2071	0.21297	0.1986	0.1956	0.2048
	4	**0.2755**	0.2652	0.2648	0.27177	0.2705	0.26439	0.269	0.264	0.2719
	6	**0.3689**	0.3324	0.3327	0.35058	0.356	0.32192	0.3287	0.328	0.3331
	8	0.3728	0.3932	0.4401	**0.44291**	0.4387	0.38256	0.3967	0.4136	0.4366
	10	0.5106	0.4457	0.4556	**0.55423**	0.4717	0.45517	0.4325	0.4319	0.4519
	12	0.4953	0.4973	0.5229	**0.62245**	0.5312	0.45102	0.4634	0.434	0.5848
IMG11	2	**0.127**	0.0972	0.0938	0.1177	0.0982	0.12642	0.1127	0.1238	0.1224
	4	**0.6039**	0.5141	0.1251	0.4588	0.1196	0.13378	0.157	0.1857	0.1515
	6	**0.6156**	0.5061	0.318	0.5053	0.1505	0.1675	0.2672	0.1929	0.1696
	8	**0.6233**	0.6213	0.3206	0.4867	0.1707	0.18515	0.2974	0.199	0.2947
	10	0.6156	**0.6397**	0.4198	0.4094	0.1962	0.20444	0.2762	0.2406	0.3091
	12	**0.6381**	0.5706	0.3833	0.3386	0.1935	0.23182	0.3256	0.3119	0.4314
IMG12	2	**0.123**	0.093	0.0894	0.1075	0.0923	0.12177	0.1082	0.1206	0.1183
	4	**0.6107**	0.4856	0.1251	0.4292	0.1135	0.12941	0.1568	0.2046	0.1346
	6	0.6188	**0.6446**	0.2753	0.4641	0.1481	0.16044	0.3807	0.2383	0.1866
	8	0.6209	**0.6595**	0.2624	0.395	0.1621	0.16563	0.3144	0.2601	0.2813
	10	**0.6334**	0.5977	0.3889	0.3555	0.1866	0.18784	0.3143	0.2677	0.336
	12	0.628	**0.6731**	0.3708	0.3632	0.181	0.18357	0.301	0.3026	0.367
IMG13	2	0.1308	0.1019	0.0999	0.1174	0.1015	**0.1309**	0.1104	0.1266	0.1258
	4	0.6161	**0.6251**	0.1285	0.3491	0.1264	0.14005	0.1599	0.1996	0.143
	6	0.6391	**0.6634**	0.2136	0.4744	0.1597	0.2103	0.3196	0.1938	0.2192
	8	**0.6596**	0.6235	0.2835	0.4486	0.1716	0.17985	0.2602	0.2405	0.2617
	10	0.6672	**0.6735**	0.4509	0.3958	0.1918	0.18981	0.4427	0.2331	0.308
	12	0.6569	**0.7039**	0.3816	0.3768	0.1935	0.20815	0.3845	0.2932	0.3506
IMG14	2	**0.1222**	0.0871	0.0817	0.11	0.0858	0.11747	0.1127	0.1193	0.1173
	4	0.5504	**0.5942**	0.1076	0.4879	0.1056	0.12454	0.1657	0.153	0.1303
	6	0.6047	**0.6654**	0.284	0.3719	0.14	0.16086	0.278	0.1767	0.1786
	8	0.6086	**0.6612**	0.3644	0.4347	0.1567	0.16576	0.3029	0.2067	0.2883
	10	**0.7042**	0.6054	0.3122	0.4226	0.1823	0.21357	0.3727	0.2595	0.2648
	12	0.6563	**0.6751**	0.3607	0.4683	0.1789	0.18332	0.4117	0.2069	0.3503
IMG15	2	**0.1369**	0.0947	0.0847	0.1095	0.0903	0.12883	0.1022	0.1259	0.1234
	4	**0.6307**	0.5719	0.1538	0.3939	0.1231	0.14474	0.1786	0.2821	0.1503
	6	**0.6477**	0.6342	0.3031	0.4167	0.1675	0.20663	0.2708	0.2932	0.2269
	8	0.6409	**0.6596**	0.3132	0.4057	0.1807	0.2031	0.3459	0.2377	0.2993
	10	**0.7309**	0.5408	0.3949	0.4582	0.2125	0.2049	0.3391	0.27	0.336
	12	**0.7274**	0.699	0.286	0.4213	0.2181	0.41515	0.4072	0.2444	0.3385
IMG16	2	**0.1283**	0.1015	0.0971	0.1173	0.1004	0.1209	0.1168	0.1258	0.1239
	4	0.5954	**0.6127**	0.1277	0.3375	0.1234	0.13401	0.1535	0.2107	0.1373
	6	0.62	**0.6936**	0.2521	0.4774	0.1555	0.16542	0.2008	0.2094	0.2319
	8	0.5966	**0.6261**	0.2387	0.3864	0.1915	0.19226	0.3075	0.2921	0.2624
	10	**0.6548**	0.5668	0.3301	0.3588	0.1848	0.22063	0.3506	0.3518	0.3779
	12	**0.7228**	0.6883	0.4568	0.3797	0.1995	0.22678	0.3945	0.2968	0.345
IMG17	2	0.1814	0.1573	0.1523	0.1661	0.1583	**0.1828**	0.1642	0.1711	0.1715
	4	0.5712	**0.5906**	0.3691	0.536	0.2176	0.22673	0.2923	0.301	0.258
	6	**0.6723**	0.6072	0.424	0.4758	0.2628	0.26024	0.4348	0.2928	0.3466
	8	**0.6581**	0.6316	0.5472	0.5281	0.2973	0.3044	0.4516	0.3542	0.3528
	10	**0.6891**	0.6617	0.47	0.4793	0.2881	0.30543	0.4284	0.3136	0.4522
	12	**0.666**	0.665	0.502	0.4958	0.2957	0.29317	0.481	0.338	0.4162
IMG18	2	**0.129**	0.1031	0.0982	0.1158	0.1019	0.11722	0.1086	0.1256	0.1253
	4	**0.6499**	0.584	0.1327	0.4327	0.129	0.14041	0.1647	0.21	0.1655
	6	**0.6687**	0.6623	0.2554	0.4476	0.1613	0.17113	0.2924	0.2092	0.2265
	8	0.6478	**0.6883**	0.2099	0.3992	0.1801	0.18057	0.3251	0.2812	0.2645
	10	0.5912	**0.6604**	0.3628	0.3749	0.2104	0.19951	0.3519	0.2564	0.3496
	12	**0.6975**	0.5765	0.3485	0.4097	0.2143	0.22413	0.3694	0.2871	0.398
IMG19	2	**0.2071**	0.1669	0.1553	0.1623	0.1654	0.19171	0.1657	0.1763	0.1767
	4	**0.5837**	0.5408	0.2936	0.5594	0.2777	0.27983	0.2742	0.3294	0.2832
	6	**0.6339**	0.5777	0.3706	0.5737	0.3189	0.33789	0.4255	0.3541	0.379
	8	0.6209	**0.6474**	0.4369	0.5365	0.3504	0.36902	0.4299	0.3758	0.4177
	10	**0.6838**	0.674	0.4988	0.488	0.3686	0.43124	0.5208	0.3879	0.4593
	12	**0.8509**	0.7159	0.6449	0.5405	0.3674	0.42728	0.5757	0.3914	0.5652
IMG20	2	**0.1436**	0.0935	0.0833	0.0994	0.0869	0.12444	0.1055	0.1243	0.1238
	4	**0.5924**	0.5035	0.1633	0.4163	0.1344	0.1514	0.198	0.2294	0.1569
	6	**0.5622**	0.5564	0.253	0.5326	0.1899	0.20619	0.351	0.274	0.2541
	8	**0.6685**	0.6411	0.3871	0.4096	0.2122	0.22868	0.3612	0.2626	0.2823
	10	0.591	**0.6488**	0.4087	0.4303	0.2272	0.23169	0.4645	0.2453	0.3206
	12	**0.6038**	0.5925	0.4752	0.4069	0.2295	0.24772	0.3211	0.3331	0.3925

Bold value represents the best finding.

**Fig. 4:**
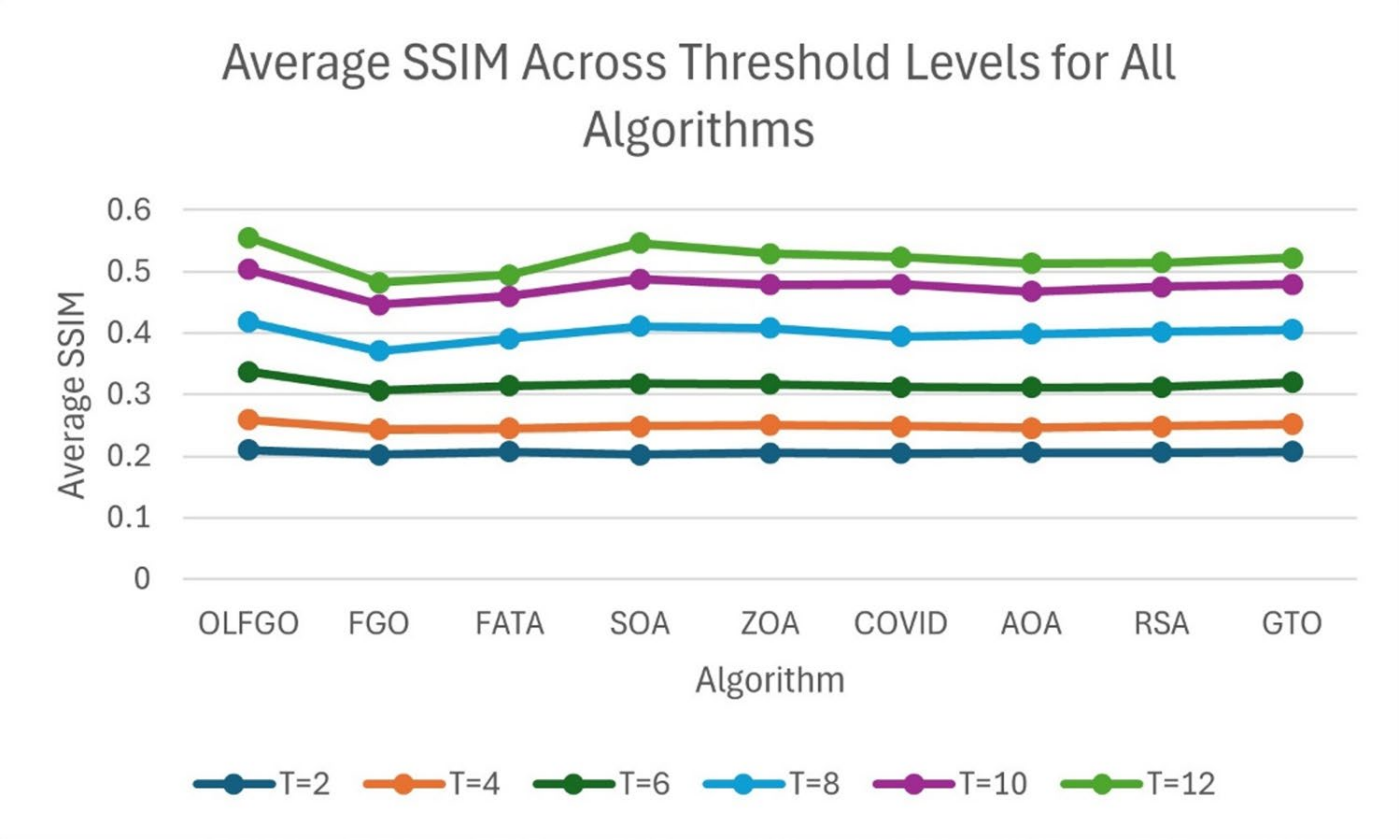
Comparison in terms of average SSIM.

The segmented and reference images’ structural similarity is measured by SSIM, and OLFGO consistently obtains the highest or nearly highest values. For example, OLFGO demonstrates strong structural fidelity in IMG3 by achieving SSIM of 0.7235 at threshold 12. The algorithm’s strong performance, even in more difficult cases like IMG6 and IMG7, highlights how well it preserves texture and edge information (Fig. 4).

Superior structural preservation is indicated by SSIM values close to 1. OLFGO continuously preserves better visual integrity in the segmented areas when compared to rival techniques.

**Table 6:** Comparison in terms of FSIM metric.

Image	Threshold	Algorithm								
		OLFGO	FGO	FATA	SOA	ZOA	COVID	AOA	RSA	GTO
IMG1	2	**0.7782**	0.7678	0.7399	0.74329	0.7538	0.73713	0.7546	0.7399	0.7748
	4	0.8333	0.8465	0.8403	0.83703	0.8373	0.8372	0.8355	**0.8486**	0.8342
	6	0.8679	0.8631	0.8931	0.88154	0.874	0.88812	0.8796	0.8756	**0.8936**
	8	0.8838	0.8766	0.8872	0.87663	0.8811	0.88083	0.8839	**0.9016**	0.8872
	10	0.8815	**0.9195**	0.8849	0.88601	0.8873	0.8874	0.879	0.8829	0.8843
	12	0.8973	0.8843	0.8947	0.90078	0.8967	**0.90235**	0.8824	0.8825	0.8934
IMG2	2	**0.8008**	0.7623	0.7834	0.75875	0.7724	0.75495	0.7612	0.7596	0.7625
	4	0.8465	**0.855**	0.8416	0.84664	0.8433	0.8507	0.8434	0.834	0.852
	6	**0.9018**	0.901	0.8665	0.8681	0.866	0.8792	0.8822	0.8938	0.8635
	8	0.8705	0.8694	0.8984	0.88905	0.8951	0.88132	0.8899	0.8817	**0.8998**
	10	**0.9017**	0.8932	0.8969	0.89811	0.9014	0.89875	0.8939	0.8861	0.8981
	12	**0.9161**	**0.9161**	0.9069	0.90977	0.9082	0.90646	0.8956	0.9021	0.9066
IMG3	2	**0.8048**	0.7084	0.7018	0.7222	0.7198	0.70252	0.7258	0.6993	0.6993
	4	**0.8389**	0.8374	0.8381	0.82605	0.8267	0.81531	0.8283	0.83	0.821
	6	0.8725	0.8868	0.8911	0.88887	0.8881	**0.89148**	0.8823	0.8828	0.8912
	8	0.8913	0.8893	0.899	0.87927	0.8928	0.88941	0.8918	0.8662	**0.9043**
	10	**0.91**	0.8695	0.8902	0.88302	0.8894	0.88175	0.8931	0.9061	0.8923
	12	**0.9383**	0.8854	0.8809	0.88481	0.8898	0.91424	0.8956	0.881	0.874
IMG4	2	**0.8133**	0.7576	0.7566	0.75845	0.7686	0.75174	0.7671	0.7511	0.8086
	4	0.8441	0.8383	0.8526	0.85347	0.8508	0.85162	0.8524	0.8473	**0.8559**
	6	0.8945	0.8952	0.9157	0.8955	0.9052	**0.9197**	0.8989	0.8745	0.9159
	8	0.8863	0.8801	0.8841	0.88021	0.8859	0.88796	0.8894	**0.9192**	0.8883
	10	**0.8893**	0.8801	0.8863	0.88764	0.889	0.8792	0.886	0.8698	0.886
	12	**0.9028**	0.8735	0.8952	0.90094	0.8987	0.88984	0.8924	0.8773	0.8937
IMG5	2	**0.7492**	0.7288	0.7225	0.72987	0.7398	0.72419	0.7278	0.7421	0.7221
	4	**0.7963**	0.7844	0.7874	0.7846	0.7829	0.78768	0.7831	0.7884	0.7868
	6	**0.8633**	0.8108	0.8149	0.82053	0.8267	0.83309	0.8367	0.7896	0.8152
	8	0.7625	0.805	0.7647	0.76762	0.79	0.79912	0.8363	**0.8619**	0.7589
	10	**0.9295**	0.7906	0.7727	0.80346	0.8076	0.791	0.8597	0.7879	0.7751
	12	**0.8659**	0.803	0.8047	0.82293	0.793	0.77026	0.8176	0.7916	0.7876
IMG6	2	0.7795	0.7807	**0.7912**	0.78179	0.7865	0.77343	0.7831	0.7786	0.7786
	4	0.8451	0.8581	0.8565	0.84862	0.84	0.8565	0.8368	**0.861**	0.8508
	6	0.8823	0.8823	0.8886	0.88126	0.8731	0.88054	0.8792	0.8646	**0.8907**
	8	**0.8989**	0.8876	0.8922	0.88717	0.895	0.89633	0.8921	0.8948	0.8959
	10	**0.9028**	0.8991	0.8902	0.89172	0.8973	0.88903	0.8931	0.8978	0.8937
	12	**0.9134**	0.8903	0.9054	0.90843	0.9025	0.9132	0.9002	0.9076	0.9023
IMG7	2	**0.7919**	0.7703	0.7697	0.77649	0.7798	0.79037	0.7788	0.777	0.7754
	4	**0.857**	0.8155	0.8408	0.83428	0.8439	0.8357	0.823	0.8239	0.8401
	6	0.8544	0.8689	0.8981	0.89676	**0.9**	0.88554	0.884	0.8937	0.8971
	8	**0.9168**	0.908	0.9021	0.90622	0.9123	0.88615	0.8943	0.9018	0.8924
	10	0.9202	0.8905	**0.9338**	0.92595	0.9323	0.91024	0.9047	0.9205	0.9269
	12	0.9136	0.9279	**0.9461**	0.93979	0.9408	0.92369	0.9111	0.8875	0.9382
IMG8	2	**0.805**	0.7911	0.7865	0.79087	0.7997	0.78294	0.7883	0.7819	0.7819
	4	**0.8489**	0.8223	0.8127	0.84076	0.8457	0.84205	0.8302	0.827	0.8131
	6	**0.8937**	0.8704	0.8927	0.8918	0.8916	0.8915	0.8887	0.8914	0.8842
	8	**0.9127**	0.897	0.9081	0.91055	0.9095	0.90269	0.8924	0.8972	0.9054
	10	0.9107	0.9049	**0.9249**	0.89917	0.9208	0.91444	0.889	0.9056	0.8966
	12	0.86	0.9059	**0.9273**	0.89123	0.925	0.90394	0.8974	0.905	0.9015
IMG9	2	0.7977	0.8227	0.7893	0.79648	0.8012	**0.83158**	0.8	0.7893	0.7893
	4	**0.8748**	0.8553	0.8364	0.85099	0.8521	0.852	0.8435	0.859	0.8605
	6	0.9097	0.9057	**0.9196**	0.9027	0.9073	0.90339	0.8963	0.8342	0.9172
	8	0.8664	0.8868	**0.922**	0.90938	0.9164	0.89225	0.8946	0.8824	0.9132
	10	0.9005	0.9132	0.928	0.9085	**0.93**	0.88818	0.8894	0.9165	0.9262
	12	0.9285	0.9005	0.9188	0.91538	**0.9355**	0.90606	0.9022	0.9062	0.9184
IMG10	2	0.7973	0.7957	0.7888	0.79328	0.7965	**0.81092**	0.7896	0.8009	0.8005
	4	**0.8448**	0.836	0.828	0.84045	0.8348	0.83078	0.8322	0.8338	0.8428
	6	0.878	0.9034	0.9071	0.8911	0.8876	0.90275	0.8905	0.8794	**0.908**
	8	**0.9132**	0.8731	0.9051	0.89998	0.9024	0.89368	0.8888	0.8749	0.9005
	10	0.902	0.9047	0.9138	0.90589	**0.9187**	0.90689	0.8923	0.9074	0.912
	12	**0.9324**	0.9018	0.8984	0.9112	0.9185	0.91209	0.8897	0.9132	0.8995
IMG11	2	**0.9053**	0.8905	0.8878	0.8809	0.8932	0.8904	0.8831	0.8876	0.8897
	4	**0.931**	0.9135	0.9121	0.921	0.9083	0.90781	0.93	0.9191	0.9103
	6	**0.9582**	0.9286	0.9513	0.9388	0.9421	0.94141	0.9299	0.9392	0.9456
	8	**0.9597**	0.9366	0.9569	0.9388	0.9494	0.94904	0.9393	0.9467	0.9506
	10	**0.9666**	0.9405	0.9613	0.9434	0.9523	0.95363	0.9498	0.9494	0.9571
	12	0.9647	0.9439	0.9619	0.9454	0.955	0.95679	**0.9656**	0.9549	0.9598
IMG12	2	**0.9055**	0.89	0.886	0.8823	0.889	0.88553	0.8808	0.884	0.8849
	4	**0.9376**	0.9103	0.9167	0.9268	0.9089	0.91007	0.9364	0.9176	0.9147
	6	**0.9482**	0.9232	0.9474	0.9307	0.9401	0.93923	0.9348	0.9356	0.9434
	8	**0.9531**	0.9329	0.9503	0.9394	0.9448	0.94042	0.9423	0.9425	0.9479
	10	**0.9595**	0.9403	0.9578	0.9424	0.9495	0.94882	0.9423	0.9479	0.9588
	12	0.952	0.938	**0.9589**	0.9465	0.9524	0.9474	0.9573	0.9508	0.9544
IMG13	2	**0.9103**	0.9034	0.9001	0.8933	0.9044	0.90859	0.8948	0.9006	0.9005
	4	**0.9443**	0.928	0.9236	0.934	0.9222	0.92374	0.9422	0.9295	0.9244
	6	**0.9563**	0.9305	0.9538	0.944	0.9488	0.9544	0.949	0.9457	0.9519
	8	0.9599	0.9405	0.9563	0.943	0.9529	0.95042	**0.963**	0.951	0.9557
	10	**0.9674**	0.948	0.9661	0.9604	0.9572	0.95269	0.9587	0.9553	0.9617
	12	**0.9666**	0.9514	0.9657	0.9643	0.9588	0.95778	0.9528	0.9603	0.963
IMG14	2	**0.8943**	0.8817	0.8771	0.8719	0.8811	0.89038	0.8717	0.8768	0.8791
	4	**0.93**	0.9071	0.9012	0.9181	0.8996	0.9005	0.8934	0.904	0.9052
	6	0.9364	0.9157	**0.9388**	0.9264	0.93	0.92878	0.9246	0.9236	0.933
	8	**0.9467**	0.922	0.9414	0.9276	0.9355	0.93398	0.9262	0.9321	0.9422
	10	0.9419	0.9243	**0.9491**	0.9308	0.9397	0.94191	0.928	0.9383	0.9417
	12	0.9524	0.9253	**0.9566**	0.9347	0.9402	0.93852	0.9376	0.9363	0.9449
IMG15	2	**0.9156**	0.903	0.8988	0.8904	0.9004	0.89951	0.8936	0.9004	0.898
	4	**0.9427**	0.9266	0.9213	0.9289	0.9162	0.92133	0.9095	0.9267	0.9223
	6	**0.9571**	0.9334	0.9526	0.9381	0.9459	0.94516	0.9412	0.9452	0.9463
	8	0.9577	0.9363	**0.9587**	0.946	0.9507	0.94693	0.9478	0.9482	0.9581
	10	**0.9672**	0.9476	0.9616	0.958	0.9558	0.95224	0.9492	0.9541	0.9596
	12	0.9634	0.9467	0.9612	**0.9786**	0.9567	0.96676	0.9757	0.9544	0.9611
IMG16	2	**0.9096**	0.8973	0.8939	0.8855	0.8965	0.89892	0.8865	0.8927	0.8941
	4	**0.9278**	0.9235	0.9148	0.92	0.9116	0.90903	0.9058	0.9215	0.9135
	6	**0.9617**	0.9278	0.9531	0.9421	0.9473	0.94405	0.9415	0.9419	0.952
	8	**0.9661**	0.9395	0.9576	0.9489	0.9548	0.95477	0.9475	0.9535	0.9528
	10	**0.9691**	0.9445	0.9642	0.9517	0.9563	0.9598	0.9451	0.9572	0.9641
	12	0.9709	0.9467	0.9663	**0.9726**	0.9594	0.96206	0.9481	0.9574	0.9638
IMG17	2	0.8994	0.8858	0.8807	0.8824	0.8871	**0.90971**	0.8798	0.8839	0.8854
	4	**0.9457**	0.9162	0.9369	0.9392	0.9315	0.93361	0.9379	0.935	0.9311
	6	**0.9667**	0.9316	0.9587	0.9656	0.9522	0.95022	0.9422	0.949	0.9536
	8	0.9685	0.9405	**0.9732**	0.9731	0.9626	0.96366	0.9566	0.9594	0.9629
	10	0.9714	0.9506	0.9749	**0.9797**	0.964	0.9661	0.9578	0.96	0.9707
	12	0.9718	0.9559	**0.9737**	0.9705	0.966	0.96262	0.9593	0.9643	0.9699
IMG18	2	**0.9079**	0.8947	0.8892	0.8828	0.8925	0.88804	0.8865	0.8909	0.8908
	4	**0.9392**	0.9182	0.9178	0.9292	0.9156	0.91573	0.9347	0.9271	0.9186
	6	0.9527	0.9278	**0.9558**	0.9419	0.9487	0.94579	0.9451	0.9442	0.9502
	8	**0.9579**	0.9316	0.957	0.9469	0.9544	0.95058	0.9434	0.951	0.9545
	10	**0.9691**	0.9443	0.9634	0.9518	0.9572	0.957	0.95	0.9532	0.9611
	12	**0.9679**	0.9485	0.9648	0.9528	0.9603	0.95993	0.9527	0.958	0.9633
IMG19	2	**0.9118**	0.8873	0.8794	0.8728	0.8875	0.91101	0.8728	0.8821	0.8846
	4	**0.946**	0.9153	0.9321	0.9409	0.9318	0.92716	0.9327	0.9329	0.9303
	6	**0.9606**	0.9238	0.9598	0.9508	0.9551	0.95268	0.9511	0.9518	0.9562
	8	0.9613	0.9358	0.9679	0.9559	0.9647	**0.96816**	0.9594	0.9606	0.9657
	10	**0.9737**	0.9501	0.9728	0.961	0.9684	0.97257	0.9611	0.9632	0.9703
	12	**0.9801**	0.9533	0.9791	0.9629	0.9698	0.97319	0.9616	0.9677	0.9753
IMG20	2	**0.9053**	0.8922	0.8862	0.8858	0.8898	0.89831	0.8888	0.8889	0.8884
	4	**0.9301**	0.9123	0.9158	0.927	0.9104	0.9091	0.9013	0.92	0.9123
	6	0.9396	0.9263	0.9416	0.9329	0.9362	0.93392	**0.9699**	0.9366	0.9429
	8	0.9467	0.9265	0.9478	0.9368	0.9426	0.94235	**0.9552**	0.9394	0.9405
	10	**0.9545**	0.9358	0.9517	0.9487	0.9441	0.94201	0.9469	0.9422	0.9453
	12	**0.9574**	0.9389	0.9557	0.941	0.9462	0.94596	0.946	0.9469	0.9522

Significant values are in bold.

**Fig. 5:**
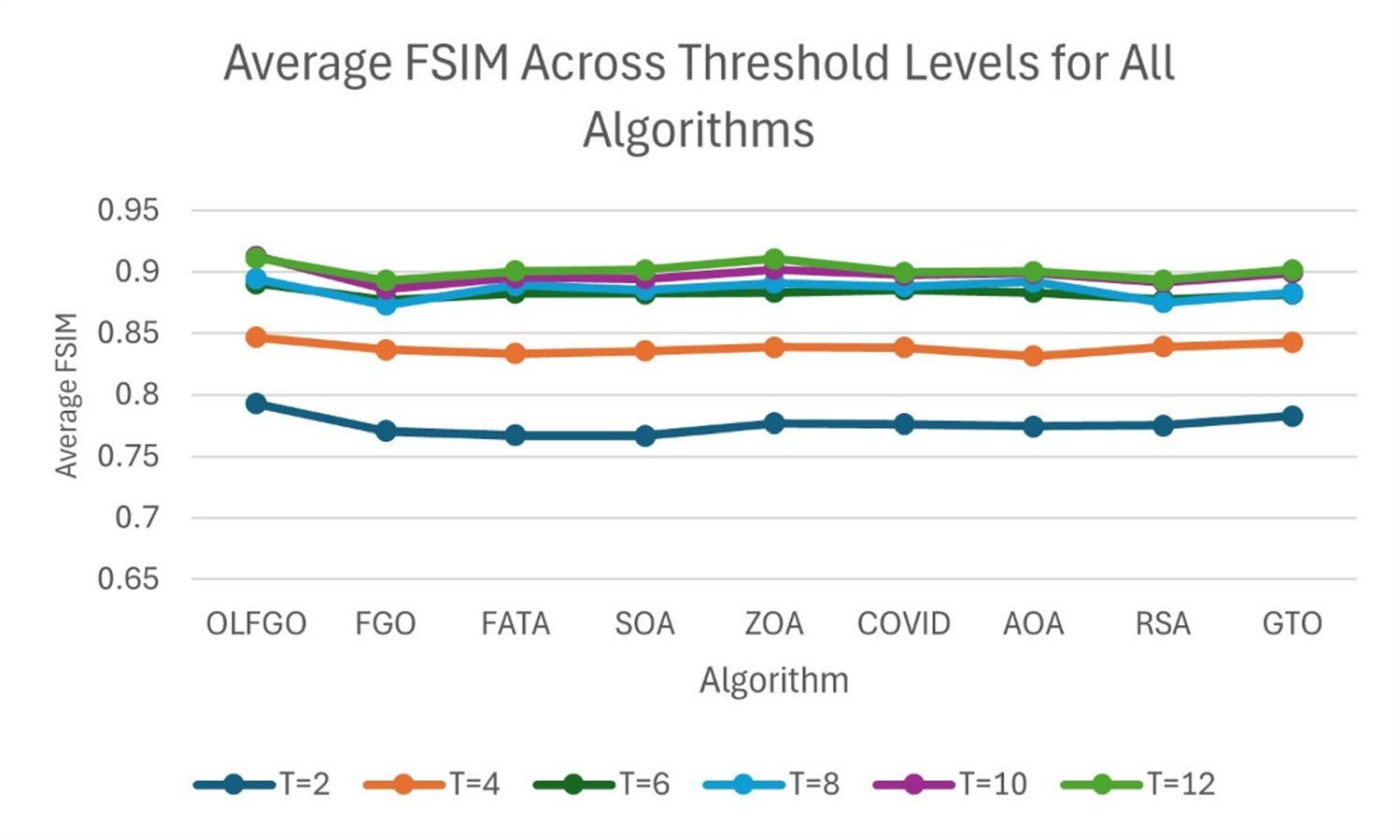
Comparison in terms of average FSIM.

The FSIM values for OLFGO are generally higher across all images, with values exceeding 0.9 in a number of cases (e.g., IMG3, IMG6, IMG7, and IMG10 at thresholds 10–12). In IMG10, for instance, OLFGO achieves 0.9324 at threshold 12, outcompeting FGO and most other algorithms (Fig. 5).

Perceptual similarity and low-level feature preservation are the main goals of FSIM. OLFGO’s ability to retain fine-grained image details following segmentation is demonstrated by its consistently high FSIM values.

**Table 7:** Comparison in terms of UQI metric.

Image	Threshold	Algorithm								
		OLFGO	FGO	FATA	SOA	ZOA	COVID	AOA	RSA	GTO
IMG1	2	**0.3302**	0.2837	0.315	0.27928	0.3058	0.28225	0.2861	0.3141	0.3167
	4	**0.4153**	0.4122	0.411	0.41079	0.4107	0.4117	0.4103	0.4088	0.4091
	6	**0.4924**	0.4859	0.4444	0.46544	0.4824	0.43432	0.4607	0.4504	0.4436
	8	0.5226	0.5065	0.5669	**0.63628**	0.606	0.62683	0.5711	0.5984	0.5514
	10	**0.7987**	0.5659	0.7683	0.76514	0.6855	0.67442	0.5951	0.7299	0.7463
	12	0.7936	0.6331	**0.8308**	0.81732	0.7665	0.61493	0.6408	0.6938	0.7959
IMG2	2	**0.253**	0.228	0.235	0.20624	0.2298	0.20901	0.2125	0.2357	0.2457
	4	**0.3711**	0.3531	0.3641	0.36529	0.3659	0.36296	0.3654	0.3625	0.3659
	6	0.3855	0.3734	**0.488**	0.48002	0.4848	0.45393	0.4439	0.4351	0.4843
	8	**0.6977**	0.5164	0.5219	0.57578	0.5362	0.51202	0.5265	0.5253	0.518
	10	0.7662	0.5715	0.6141	0.7892	0.6461	**0.80105**	0.5407	0.5878	0.6627
	12	0.7248	0.5174	0.7619	**0.85248**	0.7556	0.58206	0.5904	0.759	0.7602
IMG3	2	**0.4336**	0.4246	0.385	0.3699	0.4017	0.39611	0.376	0.3489	0.3489
	4	0.4719	0.4792	0.4858	0.48488	0.4854	**0.48904**	0.4843	0.488	0.4855
	6	**0.5401**	0.5167	0.5185	0.52296	0.5228	0.51156	0.5266	0.5107	0.5181
	8	0.5625	0.5481	0.5787	0.65336	0.5989	0.60218	0.5492	**0.6619**	0.571
	10	0.5471	0.7721	0.6541	0.78045	0.6614	**0.7859**	0.6052	0.5333	0.6327
	12	0.8399	0.628	0.6802	**0.85619**	0.7349	0.57041	0.5935	0.647	0.7933
IMG4	2	**0.3123**	0.2977	0.3007	0.26855	0.2908	0.26453	0.2725	0.257	0.2939
	4	**0.3935**	0.3848	0.3886	0.38799	0.3881	0.38795	0.3868	0.3908	0.3886
	6	0.4444	0.4144	0.4066	0.44587	0.4256	0.4113	0.4084	**0.4544**	0.4055
	8	**0.6769**	0.5179	0.5749	0.62943	0.5869	0.50264	0.57	0.4334	0.5693
	10	**0.7729**	0.7036	0.7166	0.7614	0.6943	0.68746	0.6014	0.6353	0.7141
	12	0.7076	0.7403	0.6776	**0.79997**	0.7582	0.6154	0.6428	0.7778	0.7665
IMG5	2	**0.2991**	0.2858	0.2465	0.24743	0.2723	0.27417	0.2487	0.2981	0.2397
	4	**0.3354**	0.3248	0.3316	0.33151	0.3317	0.33065	0.3309	0.3318	0.3316
	6	**0.3693**	0.342	0.3427	0.34284	0.3532	0.34217	0.3426	0.355	0.3428
	8	0.6499	0.6645	**0.7423**	0.68013	0.6191	0.44697	0.3959	0.3483	0.6448
	10	0.3648	0.5507	0.675	**0.78085**	0.6525	0.65896	0.4132	0.6431	0.728
	12	0.6307	0.6072	0.7666	**0.80761**	0.7615	0.69596	0.4807	0.7113	0.7927
IMG6	2	**0.2699**	0.2281	0.2436	0.23261	0.253	0.25453	0.2331	0.2242	0.2242
	4	**0.4224**	0.404	0.3994	0.39951	0.405	0.4032	0.4055	0.3976	0.3992
	6	**0.5114**	0.4543	0.4388	0.46209	0.4771	0.47666	0.4511	0.4807	0.435
	8	0.4903	0.498	0.5547	**0.59709**	0.5415	0.50201	0.5043	0.4766	0.5334
	10	0.6225	0.571	0.7109	**0.78274**	0.6792	0.57633	0.5479	0.5139	0.7159
	12	0.6199	0.5728	0.7746	**0.83947**	0.7737	0.75258	0.5772	0.5652	0.7853
IMG7	2	**0.3557**	0.3131	0.3046	0.32804	0.3431	0.31673	0.3198	0.3506	0.3454
	4	**0.5439**	0.5215	0.53	0.52919	0.5377	0.53047	0.5298	0.5247	0.5301
	6	**0.6252**	0.5812	0.5483	0.55423	0.5528	0.54169	0.5579	0.5742	0.5479
	8	0.5795	0.5614	0.7035	**0.70363**	0.6953	0.65466	0.6293	0.5537	0.6996
	10	**0.7593**	0.6727	0.7066	0.73941	0.7104	0.68468	0.6304	0.695	0.7094
	12	0.7427	0.7087	0.7154	**0.80627**	0.7155	0.71866	0.6903	0.6945	0.7107
IMG8	2	**0.2661**	0.2526	0.2464	0.23225	0.2483	0.23199	0.2275	0.2166	0.2166
	4	0.3382	0.3347	0.3333	0.33606	0.3373	0.33737	0.3309	**0.3421**	0.3367
	6	**0.3944**	0.3937	0.3908	0.39392	0.392	0.38555	0.3675	0.3846	0.3909
	8	0.3959	0.4003	0.4195	0.47051	0.4249	0.3977	0.4246	**0.494**	0.4164
	10	0.4002	0.4468	0.4307	0.69783	0.456	0.41711	0.4717	0.5504	**0.7579**
	12	0.71	0.6904	0.5826	0.74799	0.5206	0.74642	0.4683	0.4212	**0.7957**
IMG9	2	**0.4264**	0.3873	0.3664	0.37984	0.4206	0.39143	0.3811	0.3664	0.3664
	4	**0.5024**	0.4986	0.4997	0.50084	0.501	0.50082	0.5002	0.4957	0.5013
	6	0.5294	0.5161	0.5171	0.54699	0.5373	0.517	0.5297	**0.5981**	0.5167
	8	**0.7242**	0.6051	0.6301	0.64454	0.6262	0.62272	0.5866	0.6057	0.6274
	10	0.6802	0.6411	0.633	**0.75853**	0.663	0.71462	0.633	0.6381	0.6299
	12	**0.9055**	0.6258	0.6312	0.8149	0.6589	0.62923	0.66	0.6285	0.8028
IMG10	2	**0.3093**	0.2947	0.2572	0.26569	0.2812	0.26946	0.2586	0.2997	0.2653
	4	**0.415**	0.4103	0.4098	0.41147	0.4116	0.40927	0.4101	0.404	0.4115
	6	**0.5058**	0.4283	0.4301	0.45645	0.4622	0.42513	0.4378	0.4318	0.4299
	8	0.4697	0.511	0.5515	**0.56048**	0.5504	0.50759	0.5175	0.5478	0.5469
	10	0.6242	0.5546	0.5569	**0.7188**	0.5799	0.55391	0.5678	0.5417	0.5495
	12	0.5672	0.6505	0.7168	0.82106	0.6848	0.5481	0.6076	0.5244	**0.8582**
IMG11	2	0.2249	0.2228	0.2215	**0.226**	0.223	0.22414	0.2256	0.2238	0.2229
	4	**0.2537**	0.232	0.2281	0.2419	0.2275	0.22826	0.2323	0.2374	0.2282
	6	**0.256**	0.2386	0.2417	0.2507	0.2349	0.23572	0.2443	0.2384	0.2329
	8	**0.2522**	0.2455	0.2431	0.2506	0.2401	0.24157	0.2439	0.2415	0.2369
	10	**0.2518**	0.246	0.245	0.2497	0.2406	0.24275	0.2482	0.2415	0.2426
	12	**0.2512**	0.2461	0.2449	0.25	0.2429	0.24676	0.247	0.2445	0.2452
IMG12	2	0.2183	0.2165	0.2153	0.2197	0.2165	0.21662	**0.2199**	0.2171	0.2173
	4	**0.2429**	0.2271	0.2228	0.2368	0.2219	0.22299	0.2265	0.2309	0.2223
	6	**0.2462**	0.2348	0.2365	0.2433	0.2307	0.22637	0.2416	0.2312	0.2271
	8	**0.2438**	0.2375	0.2343	0.2403	0.2319	0.22769	0.2409	0.2348	0.2313
	10	**0.2454**	0.2399	0.2389	0.2413	0.2356	0.23804	0.2416	0.2366	0.2375
	12	**0.2437**	0.2408	0.2382	0.2428	0.2363	0.2324	0.2392	0.2373	0.2373
IMG13	2	0.2248	0.2225	0.2228	**0.2256**	0.2234	0.22445	0.2249	0.2239	0.2233
	4	**0.2384**	0.236	0.2286	**0.2384**	0.229	0.22771	0.2311	0.2375	0.2287
	6	**0.2549**	0.243	0.2421	0.2511	0.2384	0.24798	0.2457	0.242	0.2348
	8	**0.255**	0.2435	0.2434	0.25	0.242	0.23749	0.2433	0.2435	0.2405
	10	**0.2678**	0.2506	0.249	0.2512	0.2443	0.23734	0.2506	0.2458	0.2445
	12	**0.2557**	0.2506	0.2483	0.2515	0.2454	0.24452	0.2499	0.2482	0.2462
IMG14	2	0.2217	0.2196	0.2173	0.2238	0.2196	0.22155	**0.2242**	0.2207	0.2204
	4	**0.2534**	0.2323	0.2252	0.2409	0.2249	0.22514	0.2316	0.2317	0.2258
	6	**0.2509**	0.238	0.2398	0.2474	0.2353	0.23603	0.2457	0.2346	0.2313
	8	0.2423	0.2442	0.2409	0.247	0.2376	0.23611	**0.2478**	0.2382	0.2383
	10	0.2432	0.2428	0.244	**0.2512**	0.2403	0.24358	0.2473	0.2409	0.239
	12	0.2442	0.2436	0.2451	**0.2522**	0.24	0.23875	0.2504	0.2375	0.2408
IMG15	2	0.2521	0.25	0.2484	**0.2551**	0.2494	0.25157	0.253	0.2504	0.2504
	4	**0.2723**	0.2588	0.2552	0.2654	0.2539	0.25378	0.2609	0.2622	0.2552
	6	**0.2759**	0.2619	0.2659	0.2709	0.2616	0.26313	0.27	0.265	0.2591
	8	**0.272**	0.2668	0.2665	0.2705	0.2627	0.25905	0.2712	0.265	0.2633
	10	0.2732	0.2671	0.268	**0.2743**	0.2669	0.2642	0.2712	0.2671	0.2662
	12	0.2716	0.2675	0.2679	**0.2736**	0.2672	0.27123	0.2732	0.2671	0.2669
IMG16	2	**0.2234**	0.2218	0.2205	0.2232	0.2219	0.2199	0.2233	0.2219	0.2219
	4	**0.2371**	0.2352	0.2266	0.234	0.2264	0.22595	0.2292	0.2361	0.2263
	6	0.2431	0.2396	0.2365	**0.2477**	0.2345	0.2328	0.2353	0.237	0.2338
	8	0.2453	0.2459	0.2392	**0.2469**	0.2415	0.24372	0.2419	0.2439	0.2362
	10	**0.2474**	0.2438	0.2445	0.2467	0.2409	0.24706	0.2452	0.2437	0.2435
	12	0.2481	0.2462	0.2454	**0.2494**	0.2441	0.24725	0.2476	0.2426	0.2444
IMG17	2	0.3285	0.3243	0.3232	0.3269	0.3258	**0.32867**	0.3268	0.3255	0.3257
	4	**0.357**	0.3335	0.3426	0.3464	0.336	0.33588	0.3447	0.3446	0.3374
	6	**0.3595**	0.3472	0.3489	0.3516	0.3428	0.34161	0.3505	0.3444	0.3411
	8	**0.3599**	0.35	0.3513	0.353	0.3453	0.34979	0.351	0.3463	0.3445
	10	**0.365**	0.3526	0.3512	0.3533	0.3444	0.34761	0.3495	0.345	0.3486
	12	**0.3562**	0.3522	0.3503	0.3538	0.3454	0.34166	0.351	0.3461	0.3475
IMG18	2	**0.2272**	0.2253	0.2242	0.2263	0.2253	0.22677	0.2263	0.2259	0.2258
	4	**0.2558**	0.2374	0.2312	0.2443	0.2305	0.23181	0.2339	0.241	0.2312
	6	**0.2561**	0.2473	0.2435	0.2516	0.2399	0.23898	0.2443	0.2425	0.2376
	8	**0.2596**	0.2492	0.2446	0.2541	0.2448	0.23956	0.2478	0.2452	0.2414
	10	**0.2562**	0.2527	0.249	0.252	0.2454	0.24763	0.2492	0.2445	0.2476
	12	0.2527	0.2514	0.2499	**0.2538**	0.2483	0.2477	0.2507	0.2477	0.2484
IMG19	2	**0.3922**	0.3888	0.3874	0.3897	0.3903	0.39184	0.39	0.3892	0.389
	4	**0.4099**	0.3862	0.3964	0.4041	0.3955	0.3944	0.398	0.4011	0.3953
	6	**0.4104**	0.4015	0.4028	0.4088	0.3999	0.39888	0.4039	0.4016	0.3994
	8	0.406	0.4062	0.4053	**0.4078**	0.4037	0.40704	0.4066	0.4039	0.4025
	10	0.4073	0.4074	0.4061	0.4071	0.4045	0.40713	**0.4078**	0.4028	0.4044
	12	**0.4114**	0.4079	0.408	0.4093	0.4046	0.40638	0.4086	0.4052	0.4064
IMG20	2	0.2815	0.2784	0.2777	0.2864	0.2781	0.27412	**0.2868**	0.2832	0.2827
	4	0.2954	0.2812	0.2865	0.2971	0.2833	0.28341	**0.3029**	0.2893	0.2839
	6	0.2972	0.2906	0.2915	0.3043	0.2888	0.28536	**0.3093**	0.2921	0.2911
	8	0.2988	0.2923	0.293	0.3007	0.2908	0.29203	**0.3118**	0.2911	0.2881
	10	0.2997	0.2943	0.2949	0.3058	0.2909	0.28948	**0.3119**	0.2915	0.2905
	12	0.2983	0.294	0.2967	**0.3094**	0.2921	0.2923	0.3088	0.2934	0.2951

Bold value represents the best finding.

**Fig. 6:**
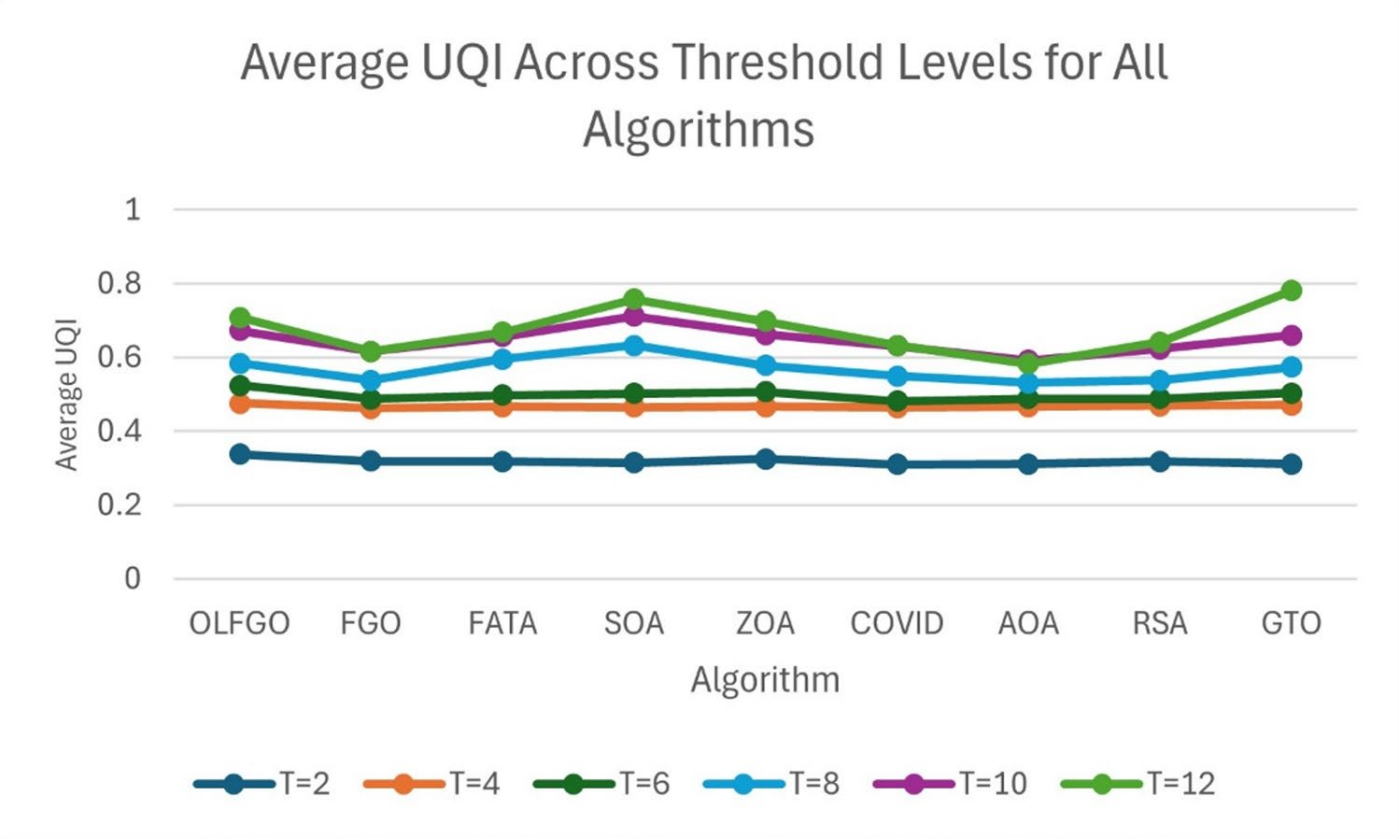
Comparison in terms of average UQI.

Significant gains in UQI are also shown by OLFGO, especially in IMG9 (0.9055) and IMG10 (0.9324) at threshold 12. OLFGO’s overall UQI performance continuously outperforms competing techniques across the entire range of thresholds, demonstrating its robustness and efficacy (Fig. 6).

When assessing global image quality, UQI considers factors like contrast, brightness, and structure. It truly stands out in terms of segmentation quality, as evidenced by the exceptionally high OLFGO scores.

**Fig. 7:**
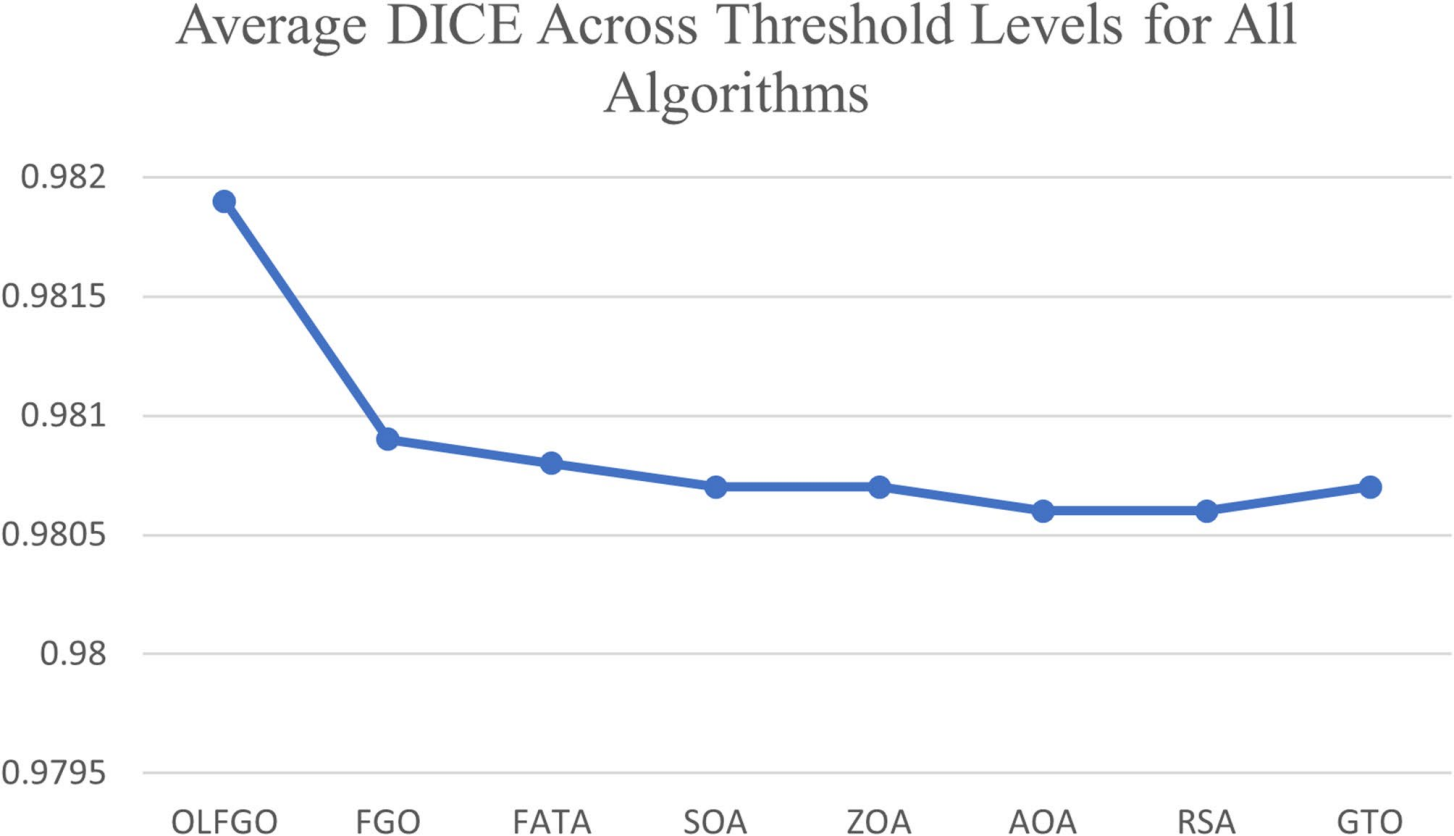
Comparison in terms of average DICE.

The proposed OLFGO method consistently yields higher Dice coefficient values for all test images, with the majority exceeding 0.9 at various threshold levels. This means that the segmentation results of OLFGO are very similar to the ground truth masks. The higher Dice scores also show that OLFGO is better than the other algorithms and gives more accurate and reliable segmentation results (Fig. 7).

**Table 8:** Comparison in terms of DICE metric.

Image	Threshold	Algorithm								
		OLFGO	FGO	FATA	SOA	ZOA	COVID	AOA	RSA	GTO
IMG11	2	**0.983**	0.9829	0.9829	0.9829	0.9829	0.9829	0.9829	0.9829	0.9829
	4	**0.9831**	0.9824	0.9827	0.9828	0.9827	0.9827	0.9827	0.9825	0.9827
	6	**0.9832**	0.9824	0.9824	0.9826	0.9826	0.9825	0.9825	0.9825	0.9826
	8	**0.9827**	0.9826	0.9824	**0.9827**	0.9825	0.9824	0.9825	0.9824	0.9825
	10	**0.9828**	0.9824	0.9825	0.9825	0.9825	0.9824	0.9824	0.9824	0.9824
	12	**0.9826**	0.9824	0.9825	0.9824	0.9824	0.9824	0.9824	0.9824	0.9824
IMG12	2	**0.9885**	0.9884	0.9884	0.9884	0.9884	0.9884	0.9884	0.9884	0.9884
	4	**0.9888**	0.988	0.9882	0.9883	0.9882	0.9881	0.9882	0.988	0.9882
	6	**0.9883**	0.9879	0.988	0.9881	0.988	0.988	0.988	0.988	0.988
	8	**0.9881**	0.988	0.9879	0.9879	0.988	0.988	0.9879	0.988	0.988
	10	**0.9881**	0.988	0.988	0.9879	0.9879	0.9879	0.9879	0.9879	0.9879
	12	**0.988**	0.9879	0.9879	0.9879	0.9879	0.9879	0.9879	0.9879	0.9879
IMG13	2	**0.9864**	0.9863	0.9863	0.9863	0.9863	0.9863	0.9863	0.9863	0.9863
	4	0.9861	0.9859	0.9861	0.986	0.9861	0.9861	**0.9862**	0.986	0.9861
	6	0.9859	0.9859	0.9859	0.9859	0.9859	0.9859	0.9859	0.9859	**0.986**
	8	**0.986**	0.9859	0.9859	0.9859	0.9859	0.9859	0.9859	0.9859	0.9859
	10	**0.986**	0.9859	0.9859	0.9859	0.9859	0.9859	0.9859	0.9859	0.9859
	12	**0.9859**	0.9858	**0.9859**	**0.9859**	**0.9859**	**0.9859**	**0.9859**	0.9858	0.9858
IMG14	2	**0.9859**	0.9858	0.9858	0.9858	0.9858	0.9858	0.9858	0.9851	0.9853
	4	**0.9864**	0.9856	0.9854	0.9855	0.9853	0.9853	0.9854	0.985	0.985
	6	**0.9858**	0.9854	0.985	0.9851	0.985	0.9849	0.9849	0.9849	0.9849
	8	**0.985**	0.9849	0.9848	0.9848	0.9849	0.9849	0.9849	0.9848	0.9849
	10	**0.9852**	0.985	0.985	0.985	0.9848	0.9848	0.9849	0.9849	0.9849
	12	**0.9851**	0.9849	0.985	0.9848	0.9848	0.9849	0.9848	0.9849	0.9848
IMG15	2	**0.9943**	**0.9943**	**0.9943**	**0.9943**	**0.9943**	**0.9943**	**0.9943**	**0.9943**	**0.9943**
	4	**0.9943**	0.994	0.9942	0.9941	0.9942	0.9942	0.9942	0.9941	0.9942
	6	**0.9941**	0.994	**0.9941**	0.994	**0.9941**	0.994	**0.9941**	**0.9941**	**0.9941**
	8	**0.9941**	**0.9941**	0.994	0.994	**0.9941**	**0.9941**	**0.9941**	0.994	**0.9941**
	10	0.994	0.994	0.994	0.994	0.994	0.994	**0.9941**	0.994	0.994
	12	**0.9942**	0.9941	0.994	0.994	0.994	0.994	0.994	0.994	0.994
IMG16	2	**0.9848**	0.9847	0.9847	0.9847	0.9847	0.9847	0.9847	0.9847	0.9847
	4	**0.9848**	0.9842	0.9845	0.9847	0.9845	0.9845	0.9845	0.9844	0.9845
	6	**0.9847**	0.9846	0.9844	0.9844	0.9843	0.9844	0.9844	0.9843	0.9844
	8	**0.9843**	0.9841	**0.9843**	0.9842	0.9842	0.9842	**0.9843**	0.9842	**0.9843**
	10	**0.9843**	0.9841	0.9842	0.9842	0.9842	0.9841	0.9842	0.9842	0.9842
	12	**0.9844**	**0.9844**	0.9842	0.9843	0.9842	0.9841	0.9842	0.9842	0.9842
IMG17	2	**0.9394**	0.9384	0.9384	0.9384	0.9386	0.9384	0.9387	0.9383	0.9384
	4	**0.9375**	0.9341	0.9342	0.9343	0.9342	0.9343	0.934	0.9342	0.9342
	6	0.9339	0.9339	0.9338	0.9338	0.9339	0.934	0.934	**0.9342**	0.934
	8	**0.9342**	0.9338	0.9339	0.9341	0.9338	0.9338	0.9338	0.934	0.9338
	10	0.9338	0.9338	0.9338	0.9338	0.9338	0.9338	0.9338	**0.9339**	0.9338
	12	**0.9342**	0.9339	0.9339	0.9338	0.9338	0.9338	0.9338	0.9338	0.9338
IMG18	2	**0.9836**	**0.9836**	**0.9836**	**0.9836**	**0.9836**	**0.9836**	**0.9836**	**0.9836**	**0.9836**
	4	**0.9834**	0.9831	**0.9834**	0.9832	**0.9834**	0.9833	**0.9834**	0.9832	**0.9834**
	6	**0.9833**	0.983	0.9831	0.983	0.9832	0.9832	0.9832	0.9832	0.9832
	8	**0.9832**	0.983	0.9831	0.983	0.9831	0.9831	0.9831	0.9831	0.9831
	10	**0.9833**	**0.9833**	0.9832	0.983	0.9831	0.9831	0.9831	0.9831	0.9831
	12	**0.9833**	0.9832	0.983	0.983	0.983	0.9831	0.9831	0.9831	0.9831
IMG19	2	**0.9461**	**0.9461**	0.9454	0.9454	0.9457	0.9454	0.9454	0.9451	0.9454
	4	**0.9427**	0.9416	0.94	0.9365	0.9399	0.942	0.9399	0.9364	0.94
	6	**0.9374**	0.935	0.9359	0.935	0.9363	0.9373	0.9367	0.9361	0.937
	8	**0.9373**	0.9348	0.9364	0.935	0.9351	0.9345	0.9353	0.9354	0.9355
	10	**0.9369**	0.9347	0.9348	0.9365	0.9349	0.9346	0.9352	0.9358	0.9349
	12	**0.9364**	0.935	0.9355	0.9346	0.9359	0.9346	0.9351	0.9348	0.9347
IMG20	2	**0.9957**	**0.9957**	**0.9957**	**0.9957**	**0.9957**	**0.9957**	**0.9957**	**0.9957**	**0.9957**
	4	**0.9955**	0.9952	**0.9955**	0.9953	**0.9955**	0.9954	**0.9955**	0.9953	**0.9955**
	6	**0.9955**	0.9952	0.9953	0.9954	0.9953	0.9954	0.9953	0.9953	0.9953
	8	**0.9954**	0.9952	0.9952	0.9952	0.9952	0.9952	0.9952	0.9952	0.9953
	10	**0.9954**	0.9952	0.9952	0.9953	0.9952	0.9953	0.9952	0.9952	0.9952
	12	**0.9954**	**0.9954**	0.9952	0.9953	0.9952	0.9952	**0.9954**	0.9952	0.9952

**Fig. 8:**
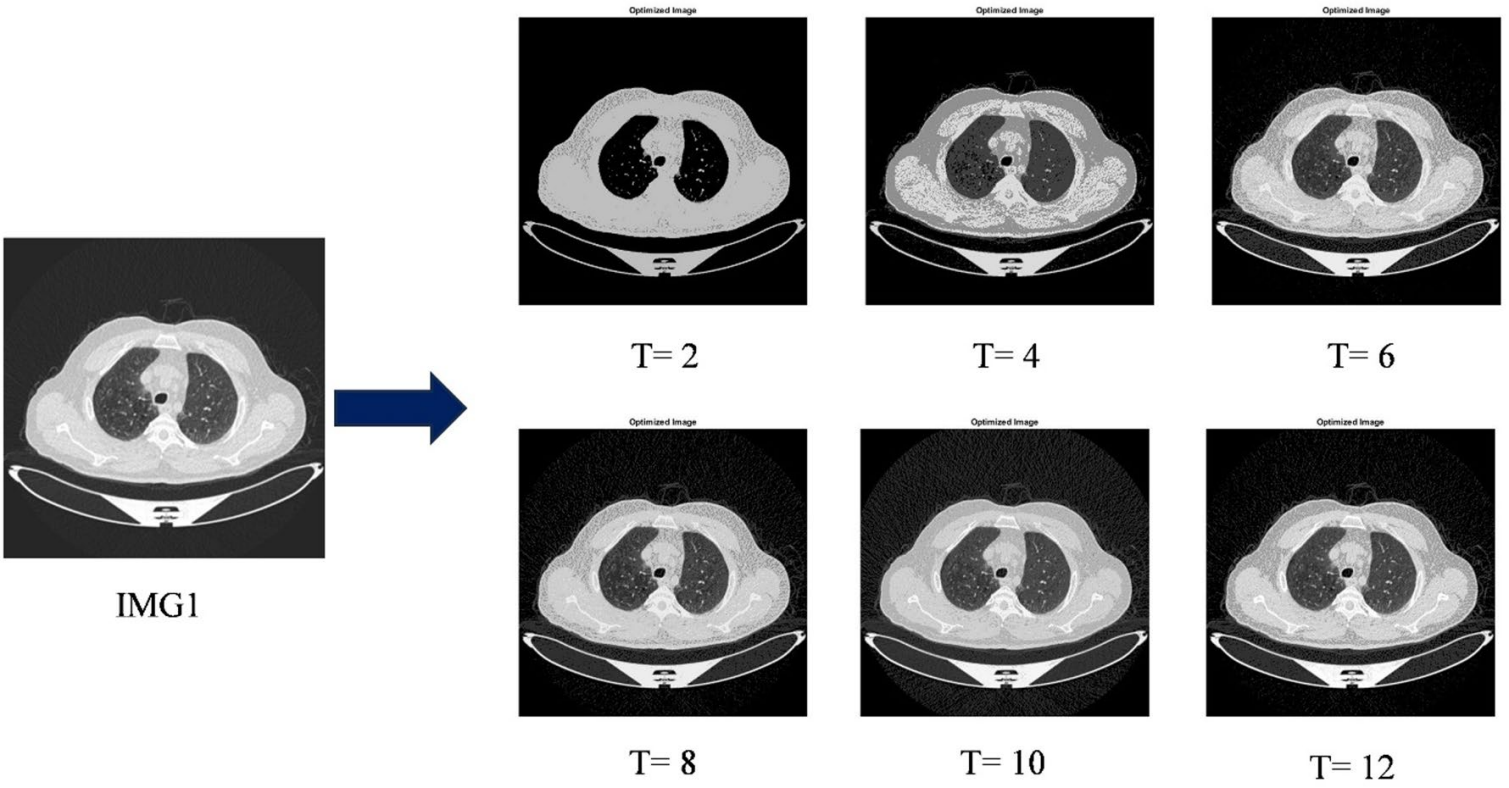
Segmentation results of CT image 1 using the proposed OLFGO algorithm at multiple threshold levels, illustrating the extraction of lung anatomical structures and pathological regions from the CT image.

**Fig. 9:**
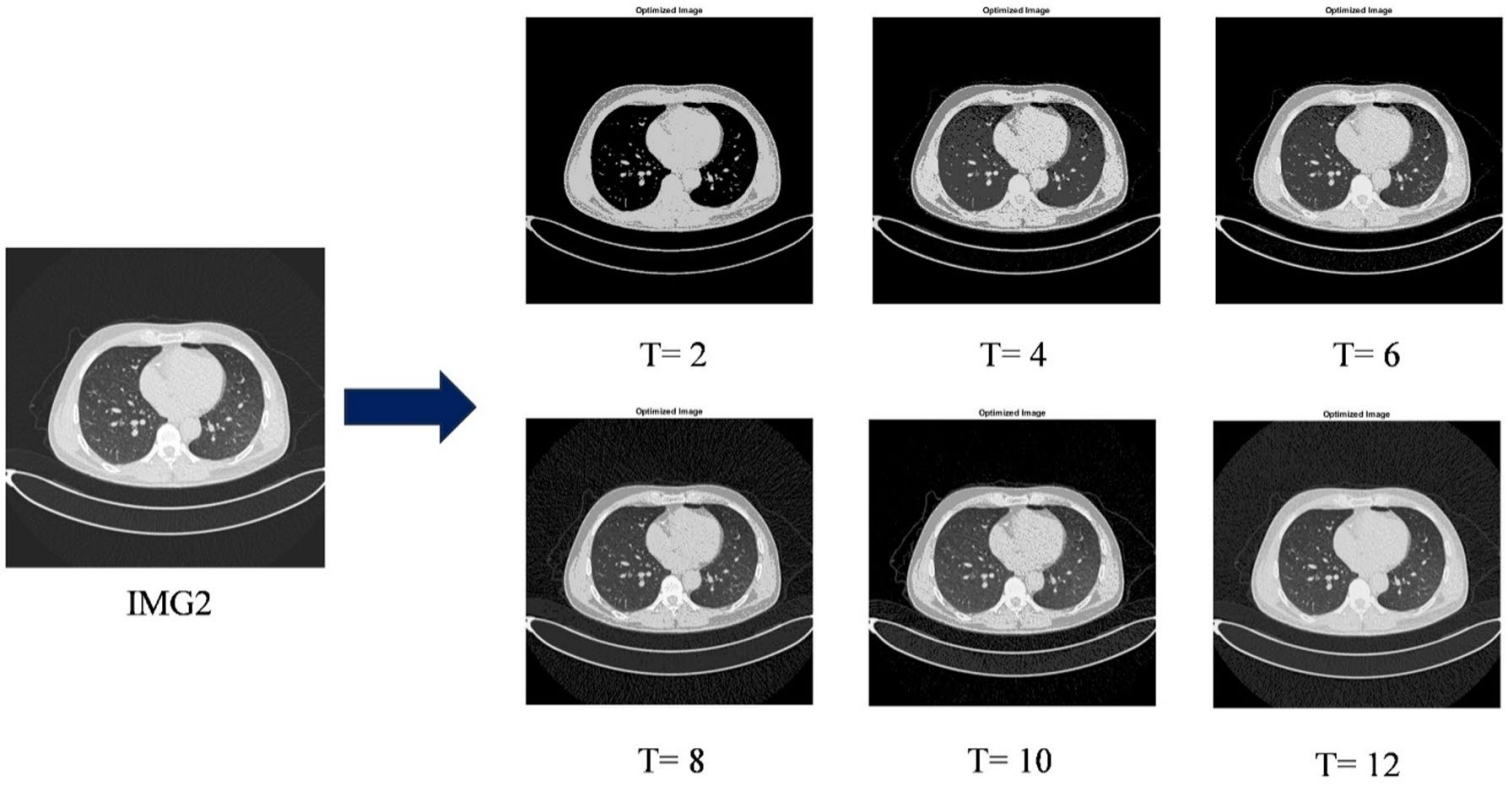
Segmentation results of CT image 2 using the proposed OLFGO algorithm at multiple threshold levels, illustrating the extraction of lung anatomical structures and pathological regions from the CT image.

**Fig. 10:**
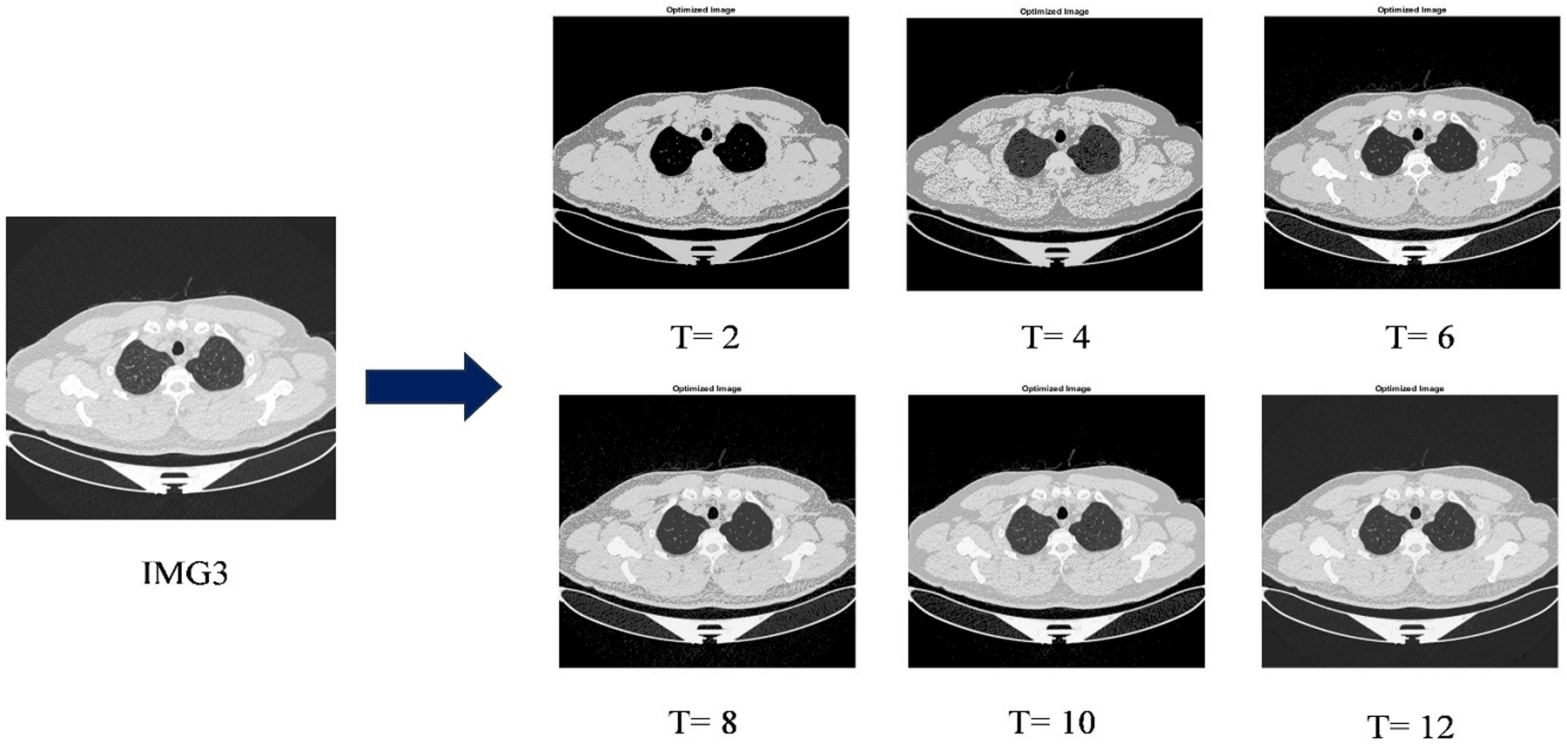
Segmentation results of CT image 3 using the proposed OLFGO algorithm at multiple threshold levels, illustrating the extraction of lung anatomical structures and pathological regions from the CT image.

**Fig. 11:**
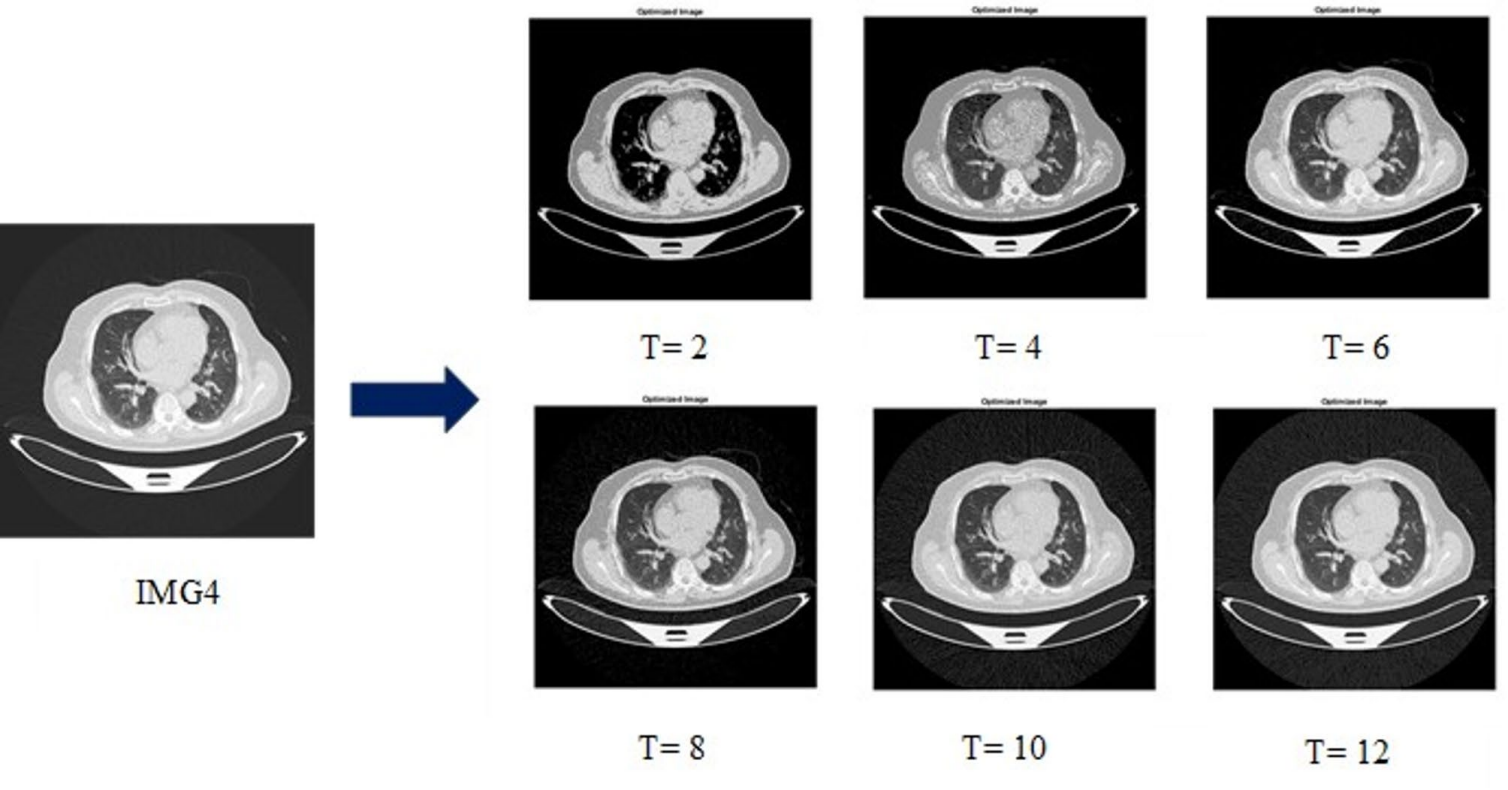
Segmentation results of CT image 4 using the proposed OLFGO algorithm at multiple threshold levels, illustrating the extraction of lung anatomical structures and pathological regions from the CT image.

**Fig. 12:**
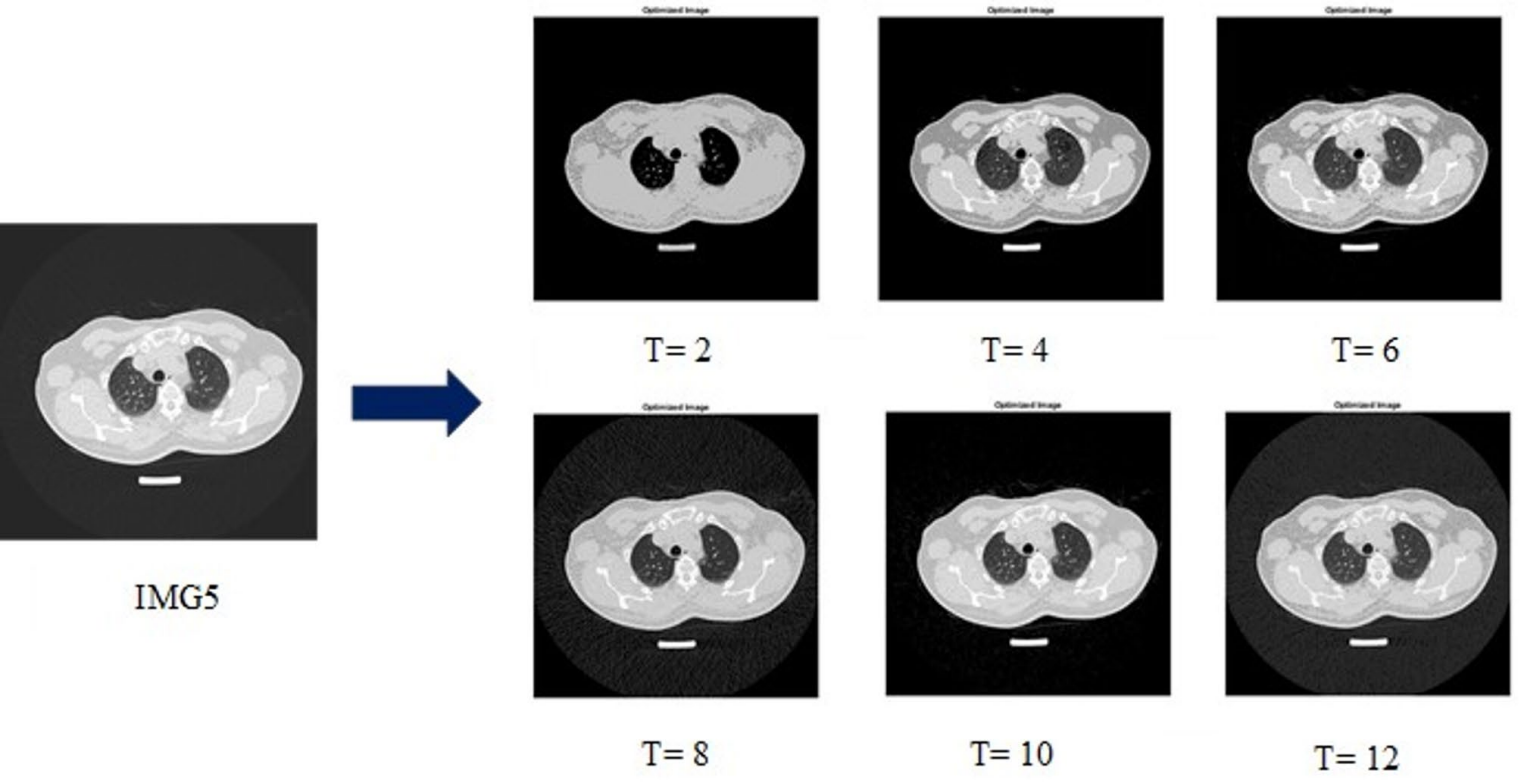
Segmentation results of CT image 5 using the proposed OLFGO algorithm at multiple threshold levels, illustrating the extraction of lung anatomical structures and pathological regions from the CT image.

**Fig. 13:**
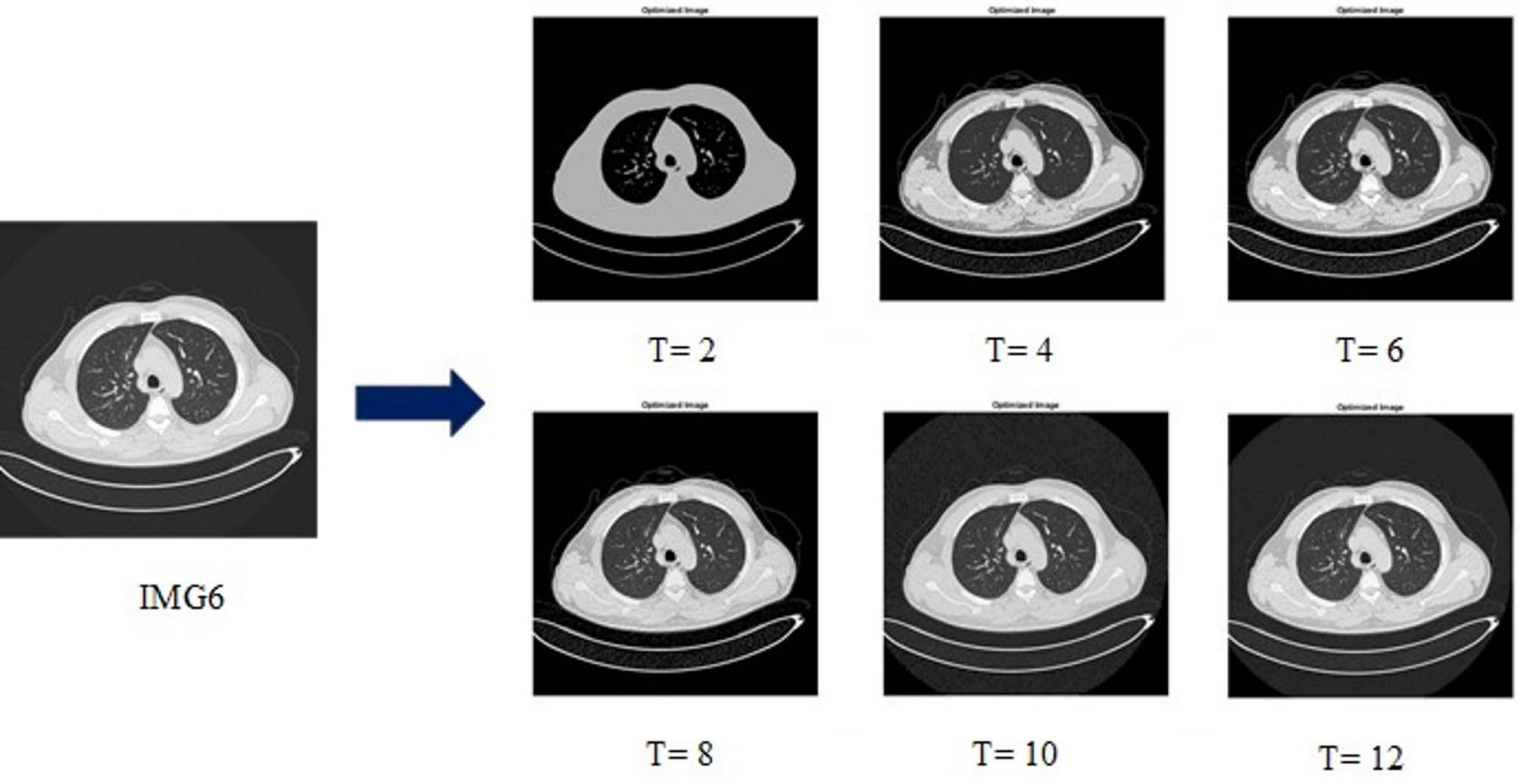
Segmentation results of CT image 6 using the proposed OLFGO algorithm at multiple threshold levels, illustrating the extraction of lung anatomical structures and pathological regions from the CT image.

**Fig. 14:**
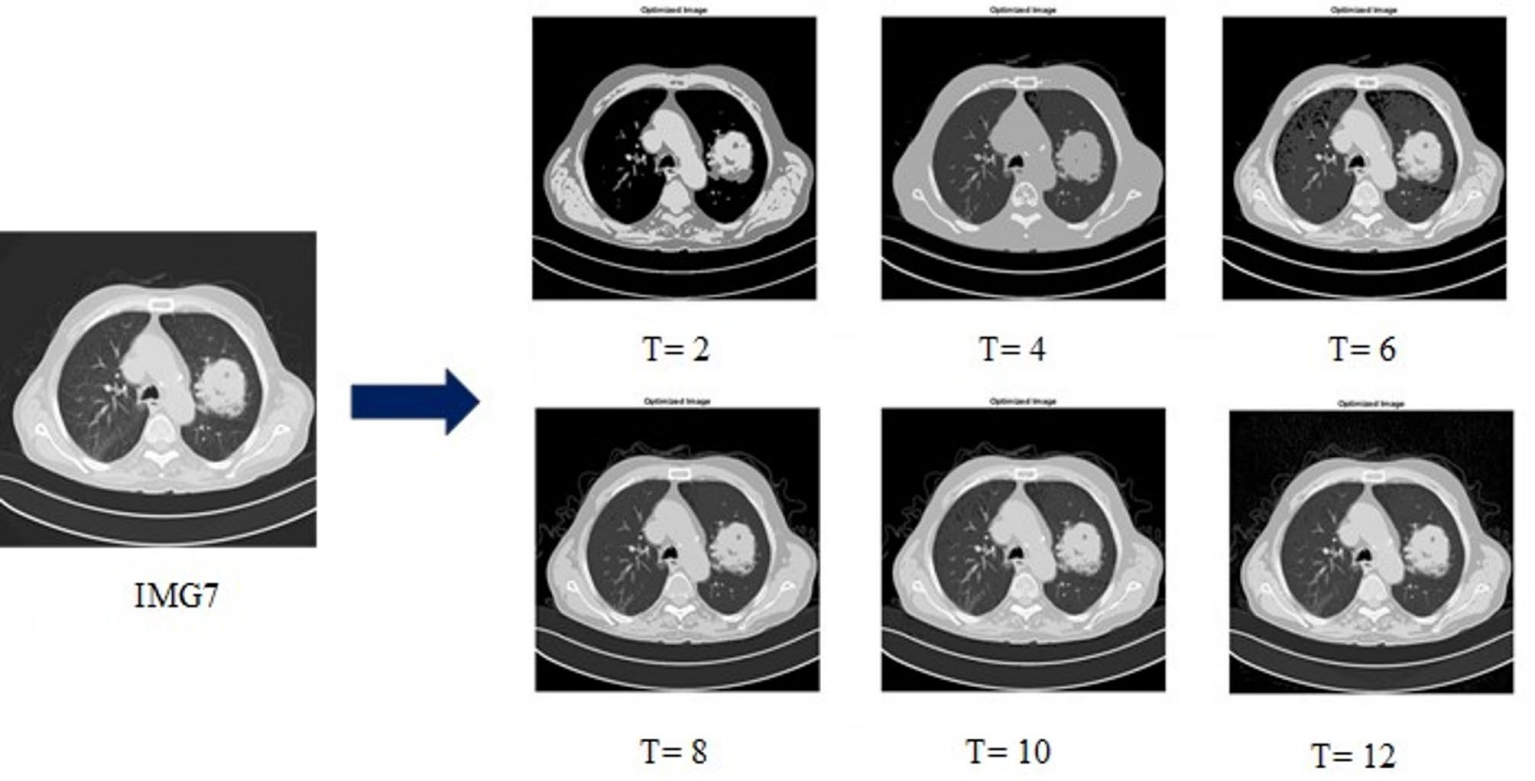
Segmentation results of CT image 7 using the proposed OLFGO algorithm at multiple threshold levels, illustrating the extraction of lung anatomical structures and pathological regions from the CT image.

**Fig. 15:**
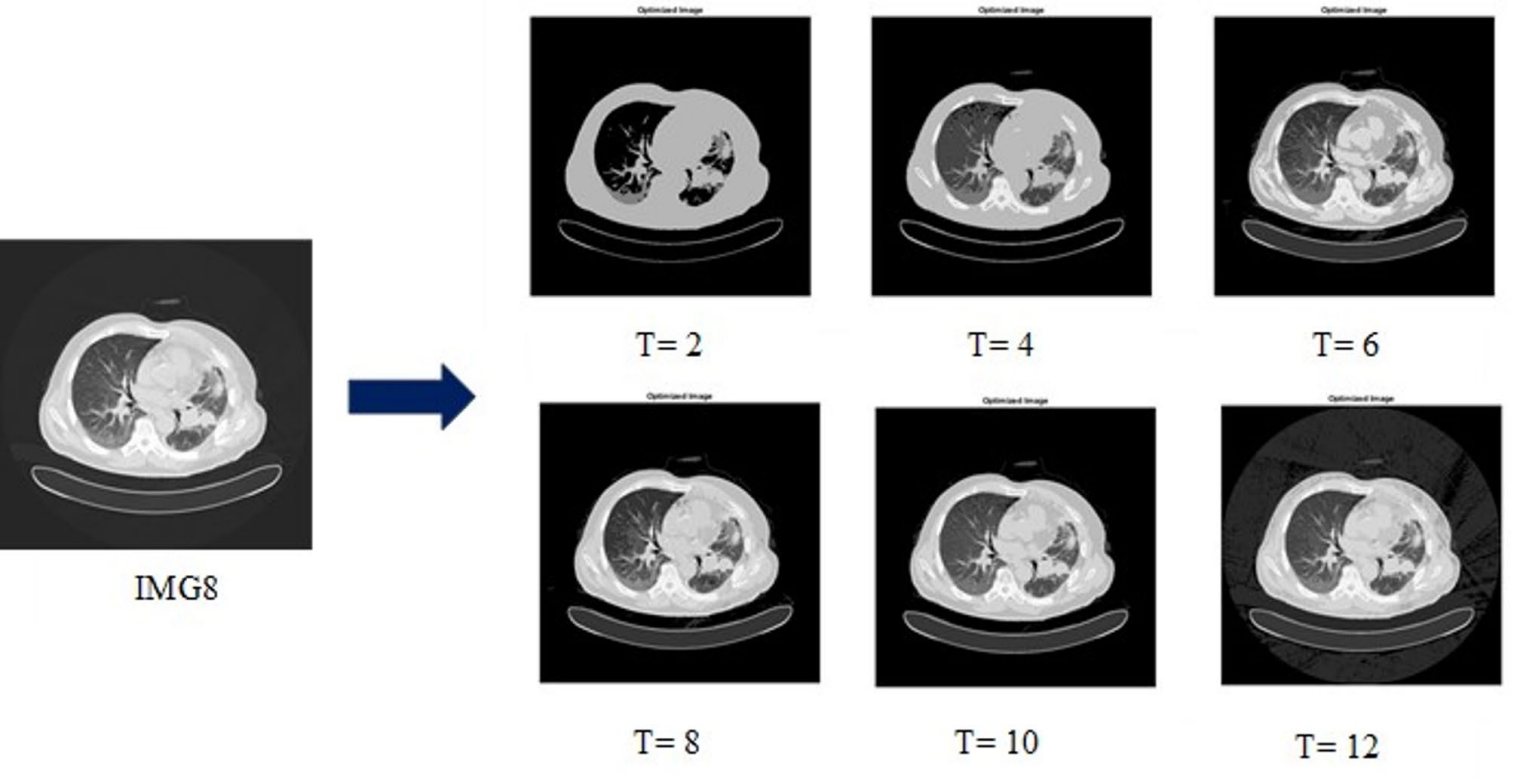
Segmentation results of CT image 8 using the proposed OLFGO algorithm at multiple threshold levels, illustrating the extraction of lung anatomical structures and pathological regions from the CT image.

**Fig. 16:**
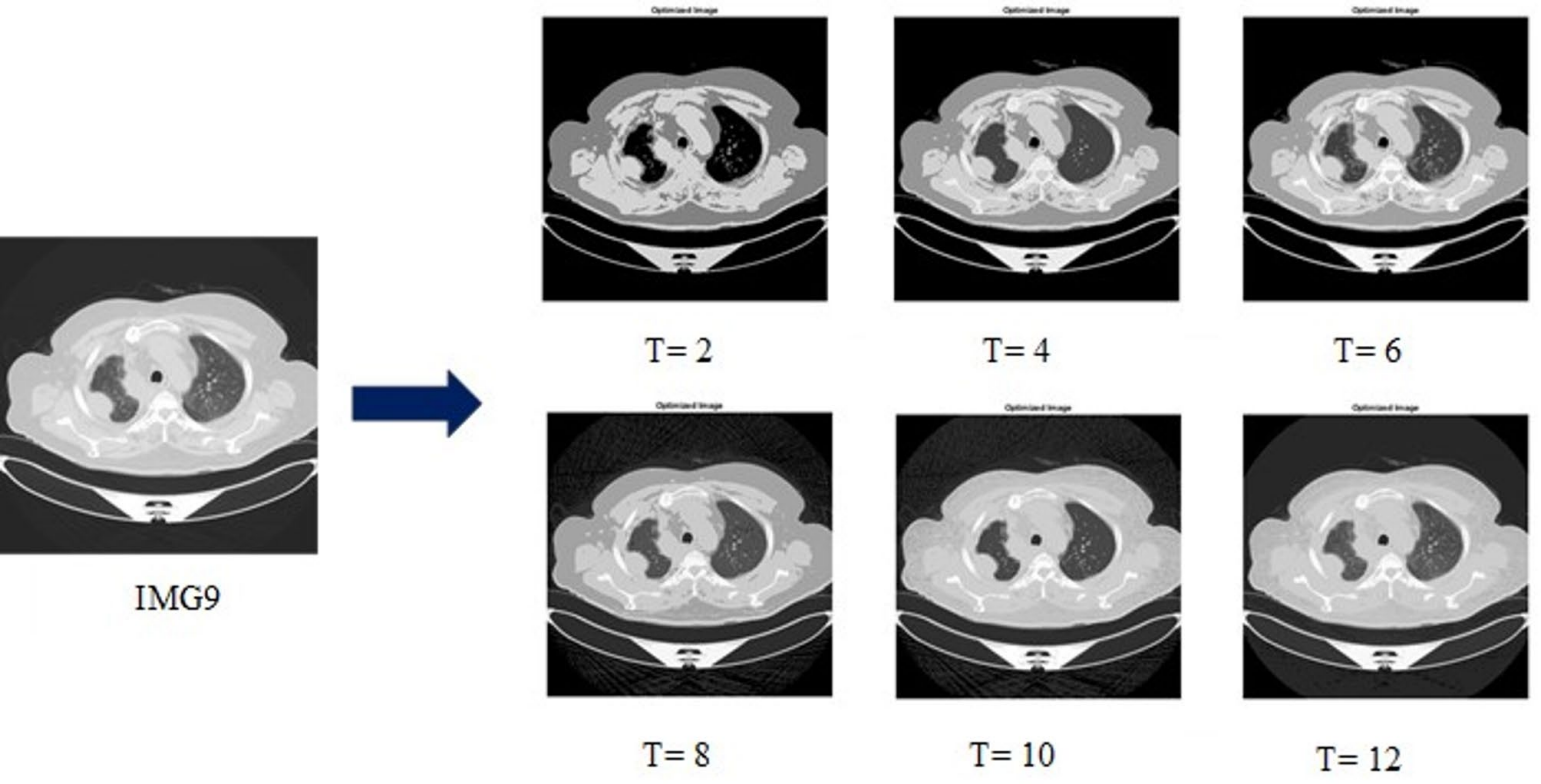
Segmentation results of CT image 9 using the proposed OLFGO algorithm at multiple threshold levels, illustrating the extraction of lung anatomical structures and pathological regions from the CT image.

**Fig. 17:**
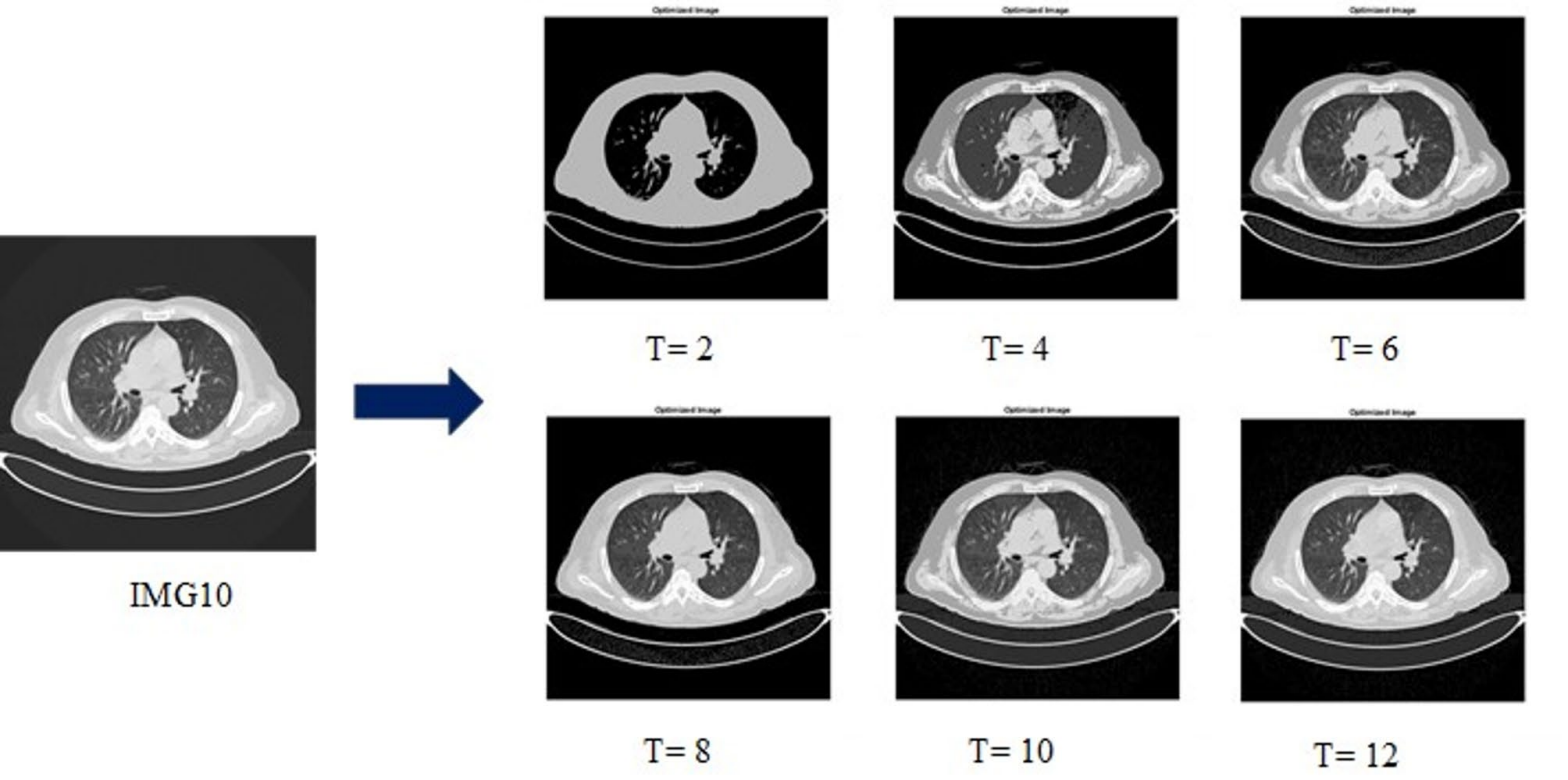
Segmentation results of CT image 10 using the proposed OLFGO algorithm at multiple threshold levels, illustrating the extraction of lung anatomical structures and pathological regions from the CT image.

**Fig. 18:**
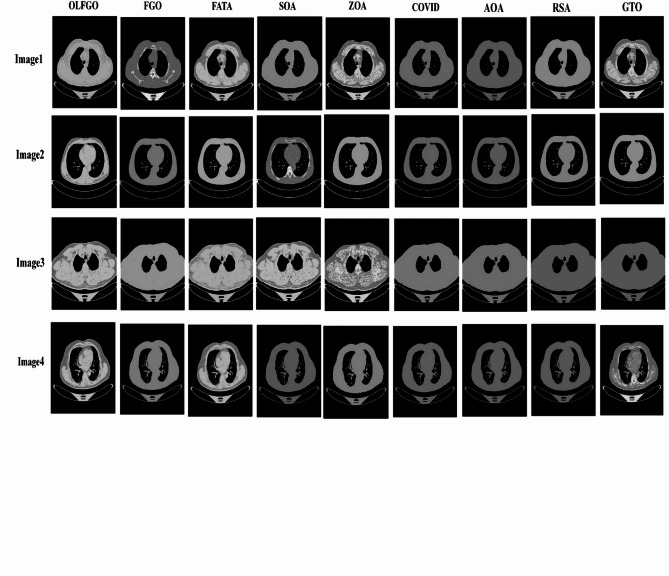
Visual comparison of CT lung image segmentation results obtained using OLFGO and different state-of-the-art optimization algorithms.

**Fig. 19:**
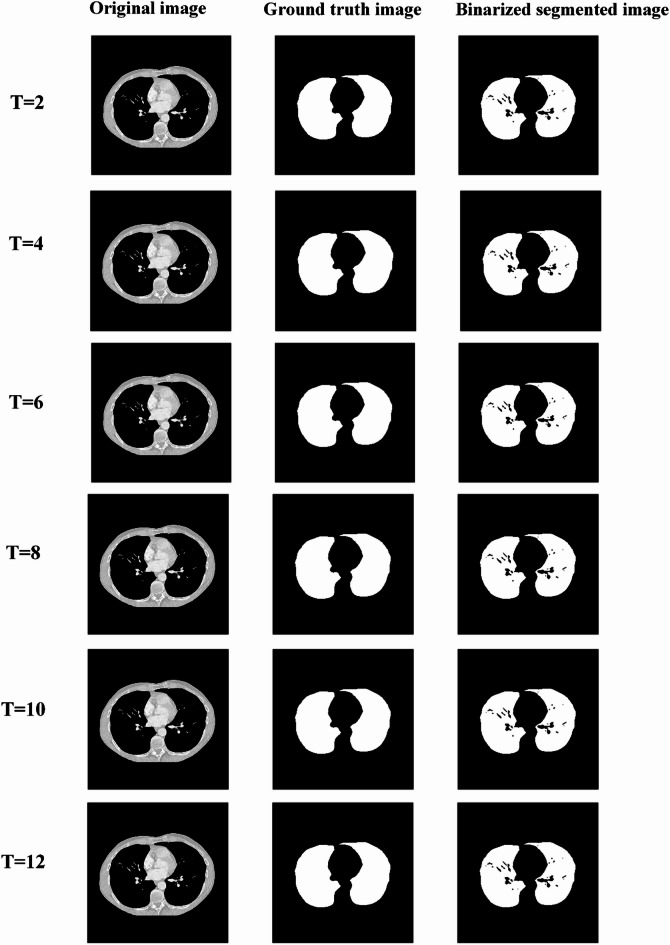
Binarized segmented image vs. ground truth of image 11 using OLFGO at multiple thresholds.

**Fig. 20:**
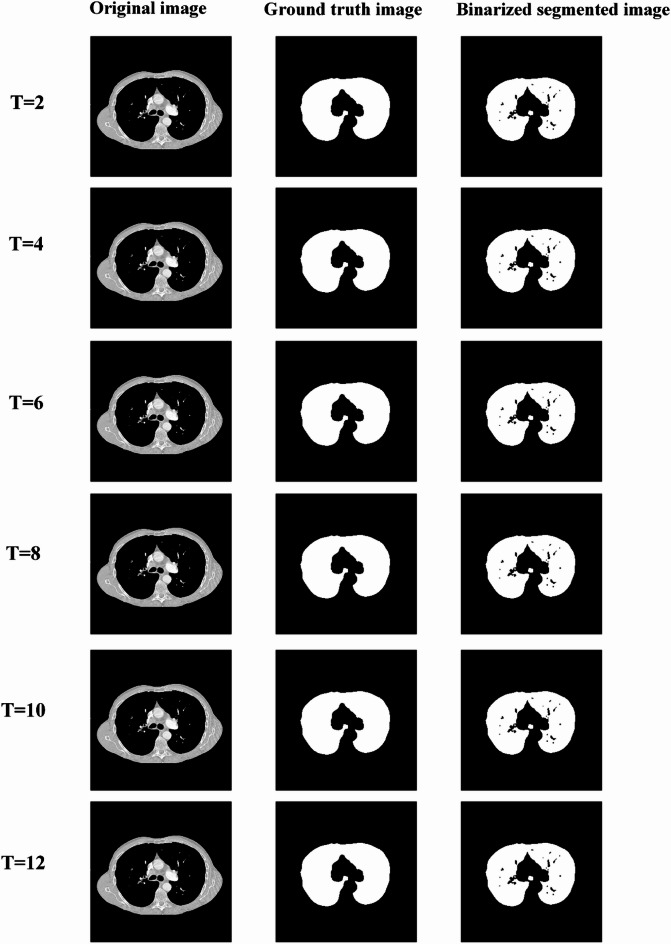
Binarized segmented image vs. ground truth of image 12 using OLFGO at multiple thresholds.

**Fig. 21:**
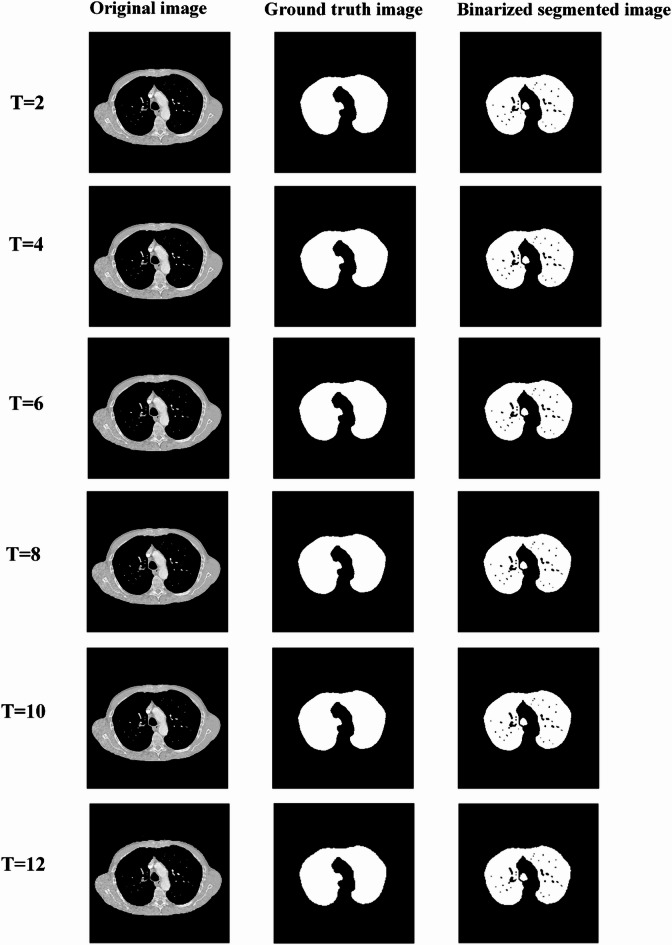
Binarized segmented image vs. ground truth of image 13 using OLFGO at multiple thresholds.

**Fig. 22:**
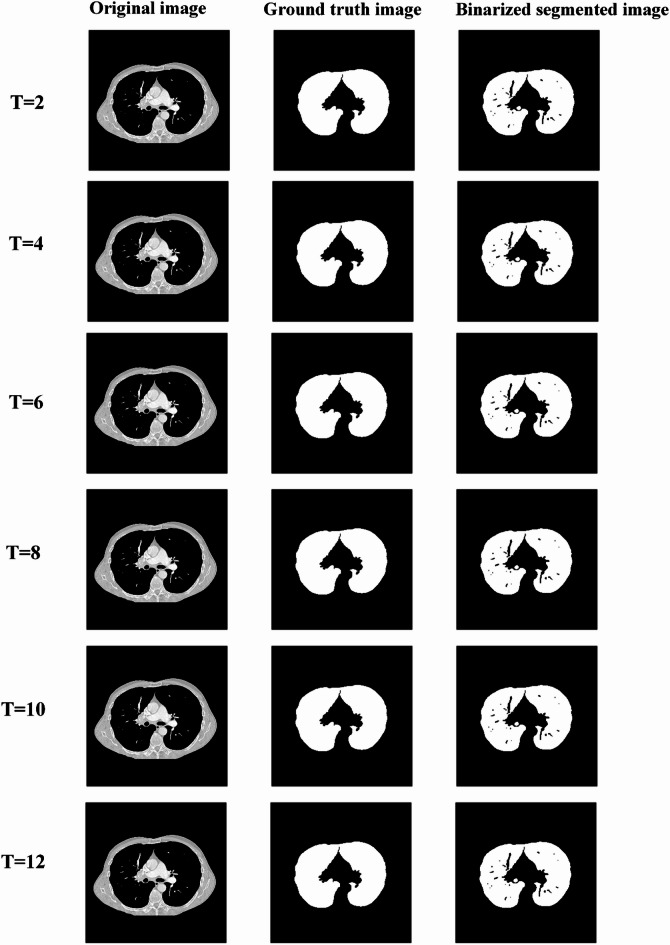
Binarized segmented image vs. ground truth of image 14 using OLFGO at multiple thresholds.

**Fig. 23:**
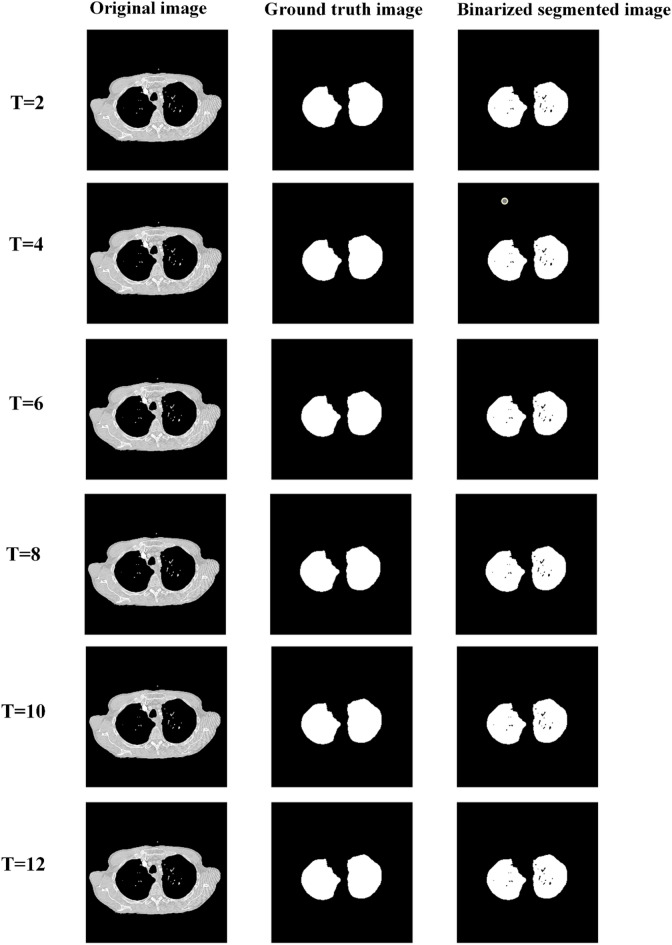
Binarized segmented image vs. ground truth of image 15 using OLFGO at multiple thresholds.

**Fig. 24:**
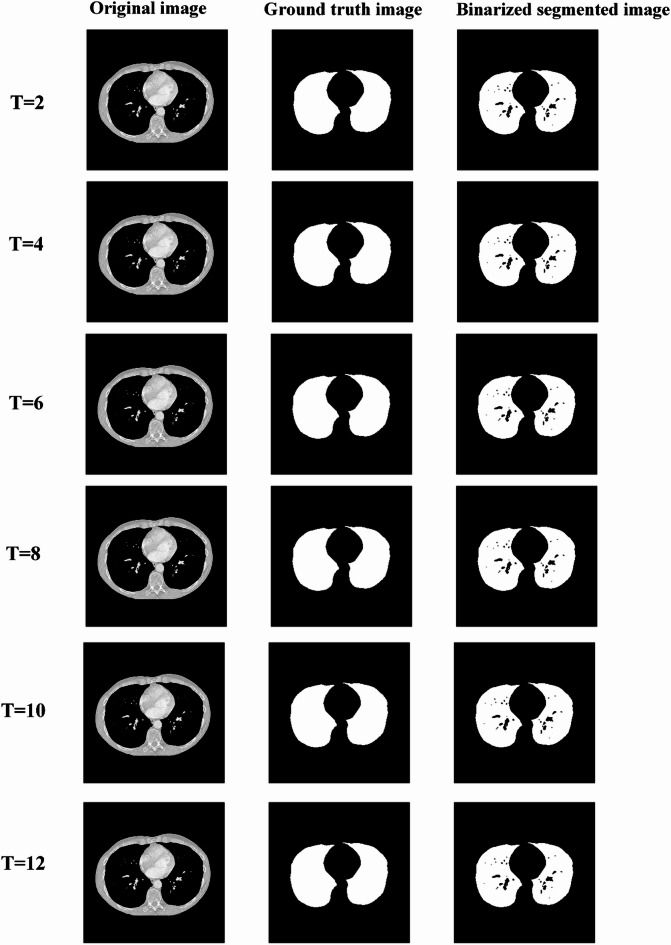
Binarized segmented image vs. ground truth of image 16 using OLFGO at multiple thresholds.

**Fig. 25:**
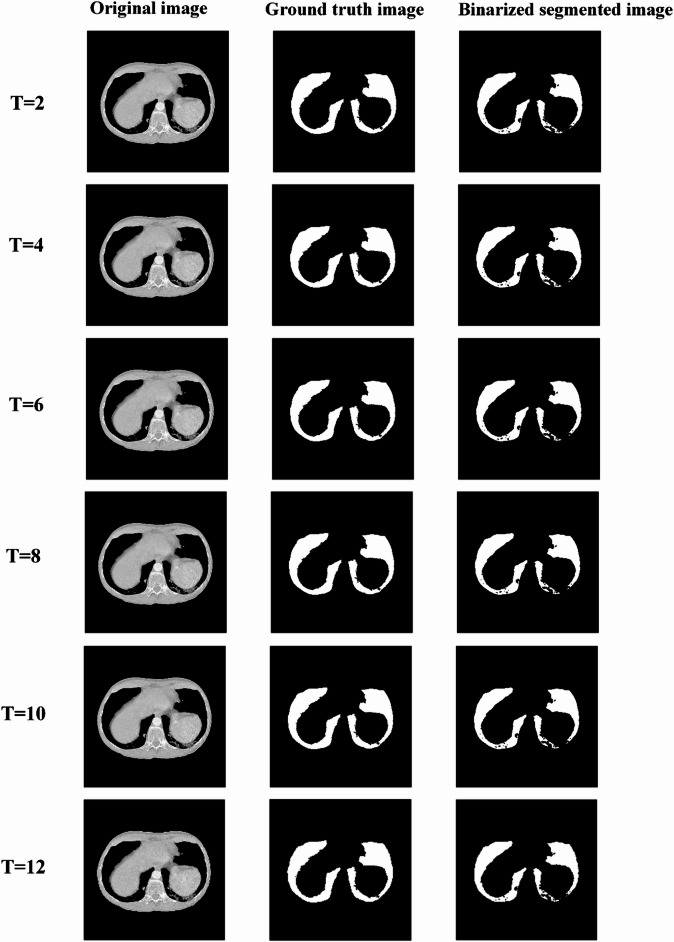
Binarized segmented image vs. ground truth of image 17 using OLFGO at multiple thresholds.

**Fig. 26:**
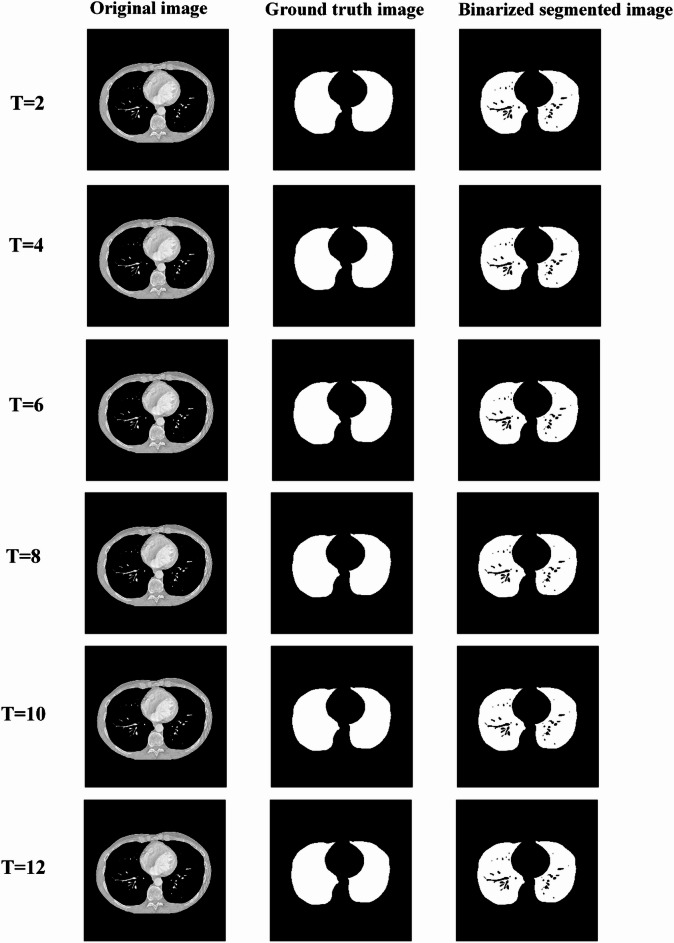
Binarized segmented image vs. ground truth of image 18 using OLFGO at multiple thresholds.

**Fig. 27:**
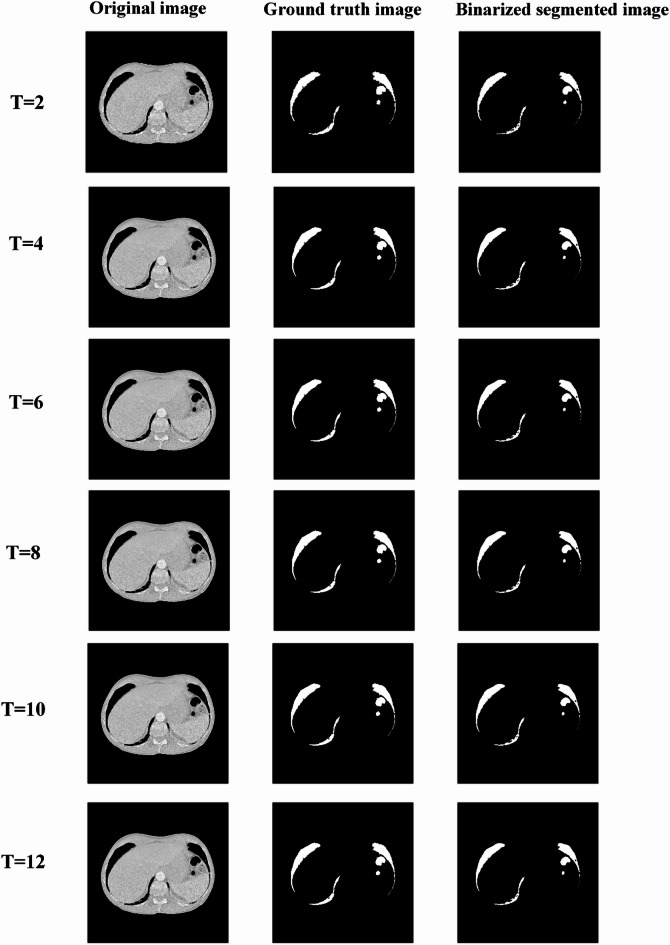
Binarized segmented image vs. ground truth of image 19 using OLFGO at multiple thresholds.

**Fig. 28:**
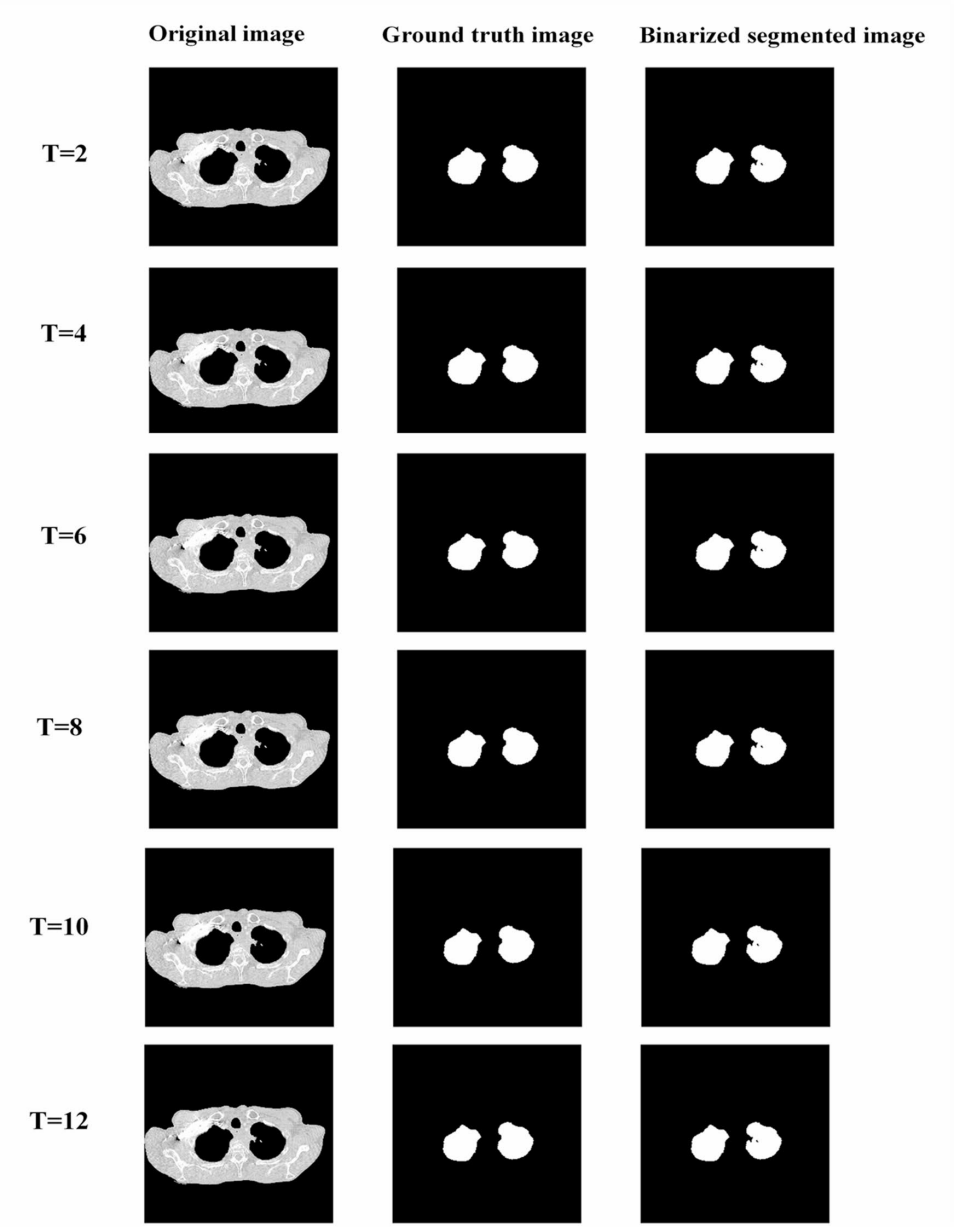
Binarized segmented image vs. ground truth of image 19 using OLFGO at multiple thresholds.

The experimental results on lung CT images nicely demonstrate the excellence and robust-ness of the proposed OLFGO algorithm. On all the quality metrics PSNR, SSIM, FSIM, UQI and DICE. OLFGO is better than benchmark algorithms for all the threshold values. Its high performance is also verified by the Friedman rank test, which ranked OLFGO as the top-performing algorithm overall. Figures 8, 9, 10, 11, 12, 13, 14, 15, 16 and 17 shows the results of segmentation on CT images 1–10 utilizing the proposed OLFGO method using various thresholds in multiple levels. This figure clearly depicts the ability of the proposed method to effectively identify the lung anatomy as well as the pathological areas using different thresholds. Figure 18 shows the qualitative comparison among the segmentations produced by applying the various optimization techniques: OLFGO, FGO, FATA, SOA, ZOA, COVID, AOA, RSA, and GTO, on representative CT images of the lungs. Based on the visual results obtained, it is evident that the suggested OLFGO technique performs the lung tissue segmentation task with significantly better performance than other optimization algorithms. The suggested optimization approach provides better accuracy in preserving the anatomic boundaries and structures for Images1 and Image4, where a few other techniques provide excessive smoothness or failure in segmenting important areas. In Image2, the proposed algorithm provides clear separation between the lung tissues and the surrounding background without any visible segmentation errors. On Image3, OLFGO produces better preservation of the textures and better delineation of the infected regions. While some of the other optimization algorithms, for instance, FGO, AOA, and RSA produce relatively smooth regions, they are not able to preserve the fine structural information. Conversely, certain optimization techniques like SOA, FATA, and ZOA result in the addition of noise to the images and the production of irregular segmentation shapes. Figures 19, 20, 21, 22, 23, 24, 25, 26, 27 and 28 present the comparison between the binary segmentation result using the proposed OLFGO approach and the respective ground truth masks for the CT images 11 to 20, considering different thresholds. These figures show that the proposed algorithm is successful in achieving a similar output as compared to the ground truth image.

.

#### Comparison between deep learning algorithms and OLFGO

We compare the proposed OLFGO method to deep learning-based segmentation models like CNN and Attention U-Net to give a full picture of how well it works. Experimental results demonstrate that OLFGO attains competitive and, in numerous instances, superior performance regarding segmentation accuracy, especially when assessed with the Dice coefficient. Large annotated datasets are very important for deep learning models like CNN and Attention U-Net to learn representative features. In situations with limited training data, as examined in this study, these models may experience overfitting or inadequate generalization, resulting in suboptimal segmentation outcomes. On the other hand, OLFGO is a metaheuristic optimization-based method that doesn’t need a training phase, so it is less affected by the size and annotation limits of the dataset. The addition of the Orthogonal Learning Strategy (OLS) to OLFGO also improves the algorithm’s ability to explore and exploit, allowing it to find the best threshold values for multilevel segmentation. This makes it easier to see the boundaries of regions and makes them more like the ground truth masks. Attention U-Net works well because it can focus on the right areas using attention mechanisms, but it still needs enough training data and computing power to work well. OLFGO, on the other hand, is a more stable and computationally efficient option, especially for small datasets. It consistently gets high Dice scores (often over 0.9) and has strong segmentation performance. The comparative analysis shows that OLFGO is a promising and effective way to segment medical images. It is more accurate, stable, and does not need large training datasets like deep learning-based methods do.

**Fig. 29:**
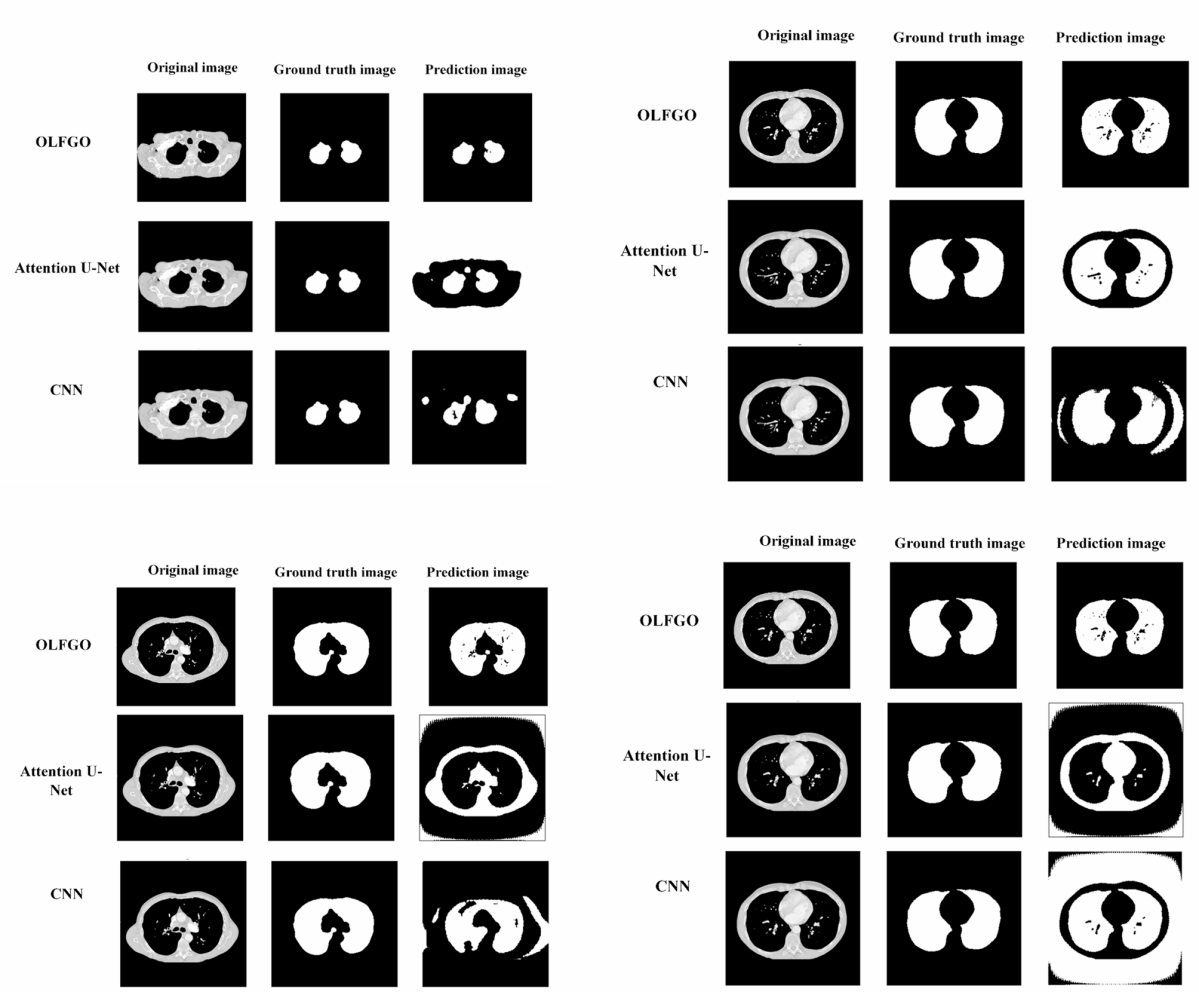
Segmentation results for different images using deep learning algorithms.

The segmentation results shown in Fig. 29 show that the proposed OLFGO method works better than both the Attention U-Net and CNN-based deep learning models. The OLFGO method gives segmentation outputs that are more in line with the ground truth masks. This shows that it is more accurate and better at defining the areas of interest.

#### CEC 2022 benchmark performance

To evaluate the general optimization capability of the introduced OLFGO algorithm, we utilized the CEC 2022 benchmark functions. These functions have been widely used to evaluate the performance of metaheuristic optimizers since they are complex, multimodal, non-separable, and scalable in nature^57^. The test suite includes several unimodal, multimodal, hybrid, and composition functions that are designed to evaluate the exploration and exploitation capabilities of optimizers. On this test, all algorithms were executed with D = 10 for 30 independent runs.

Mean and standard deviation (STD) of outcome on each function were recorded to reflect convergence stability and robustness. Table 9 provides comparative outcome of OLFGO with eight state-of-the-art algorithms: GTO, RSA, AOA, COVID, ZOA, SOA, FATA, and FGO. Optimal performance is bold faced, second-best performance is underlined.

OLFGO was assessed using 30 independent runs for each of the CEC 2022 benchmark functions (F1–F12). The findings indicate:

1. Mean Fitness: In nine of the twelve functions, OLFGO has the lowest mean error.
2. Standard Deviation (Std): The algorithm’s stable convergence behavior is demonstrated by lower Std values.

**Table 9:** Mean ± Std for CEC 2022 functions (D = 10).

Algorithms	Criteria	F1	F2	F3	F4	F5	F6	F7	F8	F9	F10	F11	F12
OLFGO	Mean	**3.00E + 02**	**4.00E + 02**	**6.00E + 02**	8.09E + 02	**9.00E + 02**	1.80E + 03	**2.00E + 03**	2.20E + 03	**2.53E + 03**	2.50E + 03	**2.62E + 03**	**2.86E + 03**
	STD	**0.00E + 00**	**1.84E-03**	**0.00E + 00**	1.92E + 00	**0.00E + 00**	4.08E-01	**8.23E-05**	4.13E-01	**0.00E + 00**	**3.00E-02**	**7.61E + 01**	**1.19E + 00**
FGO	Mean	**3.00E + 02**	4.00E + 02	**6.00E + 02**	**8.04E + 02**	**9.00E + 02**	**1.80E + 03**	2.00E + 03	**2.20E + 03**	**2.53E + 03**	**2.50E + 03**	2.74E + 03	2.86E + 03
	STD	**0.00E + 00**	7.28E-01	**0.00E + 00**	**1.58E + 00**	**0.00E + 00**	**9.38E-02**	1.64E-04	**4.08E-01**	**0.00E + 00**	3.94E-02	1.52E + 02	1.48E + 00
FATA	Mean	1.69E + 04	2.65E + 03	6.72E + 02	8.68E + 02	2.00E + 03	1.03E + 08	2.17E + 03	2.56E + 03	2.93E + 03	3.22E + 03	4.17E + 03	3.18E + 03
	STD	1.22E + 04	8.64E + 02	9.90E + 00	9.71E + 00	2.79E + 02	7.21E + 07	3.31E + 01	1.65E + 02	9.10E + 01	4.15E + 02	3.50E + 02	1.03E + 02
SOA	Mean	2.14E + 03	4.79E + 02	6.12E + 02	8.30E + 02	1.05E + 03	5.05E + 04	2.04E + 03	2.23E + 03	2.58E + 03	2.51E + 03	2.81E + 03	2.86E + 03
	STD	1.88E + 03	1.12E + 02	7.58E + 00	7.79E + 00	1.19E + 02	3.77E + 04	1.13E + 01	4.62E + 00	3.44E + 01	3.10E + 01	2.19E + 02	1.30E + 00
ZOA	Mean	6.21E + 02	4.45E + 02	6.20E + 02	8.13E + 02	1.02E + 03	3.12E + 03	2.04E + 03	2.23E + 03	2.57E + 03	2.55E + 03	2.87E + 03	2.90E + 03
	STD	3.05E + 02	2.77E + 01	8.69E + 00	4.13E + 00	6.18E + 01	1.56E + 03	1.51E + 01	3.01E + 01	5.64E + 01	6.18E + 01	2.03E + 02	2.33E + 01
COVID	Mean	2.63E + 04	1.24E + 03	6.66E + 02	8.88E + 02	2.92E + 03	1.64E + 08	2.13E + 03	2.26E + 03	2.80E + 03	2.55E + 03	3.37E + 03	2.97E + 03
	STD	7.08E + 03	4.06E + 02	1.05E + 01	1.13E + 01	4.73E + 02	1.15E + 08	2.40E + 01	8.04E + 00	6.24E + 01	3.09E + 01	2.46E + 02	4.70E + 01
AOA	Mean	1.04E + 04	1.25E + 03	6.37E + 02	8.30E + 02	1.34E + 03	2.91E + 05	2.10E + 03	2.29E + 03	2.72E + 03	2.64E + 03	3.41E + 03	3.03E + 03
	STD	4.64E + 03	4.87E + 02	9.09E + 00	1.02E + 01	1.39E + 02	1.24E + 06	3.94E + 01	8.94E + 01	3.80E + 01	1.65E + 02	3.62E + 02	6.50E + 01
RSA	Mean	8.71E + 03	1.02E + 03	6.48E + 02	8.52E + 02	1.51E + 03	8.84E + 07	2.13E + 03	2.25E + 03	2.73E + 03	2.66E + 03	3.35E + 03	2.97E + 03
	STD	3.11E + 03	5.35E + 02	7.64E + 00	9.01E + 00	1.37E + 02	1.01E + 08	3.91E + 01	1.67E + 01	4.60E + 01	1.34E + 02	4.43E + 02	7.22E + 01
GTO	Mean	**3.00E + 02**	4.10E + 02	6.05E + 02	8.23E + 02	9.50E + 02	2.01E + 03	2.03E + 03	2.22E + 03	2.53E + 03	2.52E + 03	2.66E + 03	2.87E + 03
	STD	2.07E-07	1.68E + 01	4.19E + 00	5.93E + 00	3.17E + 01	6.18E + 02	7.20E + 00	4.03E + 00	9.57E-01	4.30E + 01	1.45E + 02	3.39E + 00

**Fig. 30:**
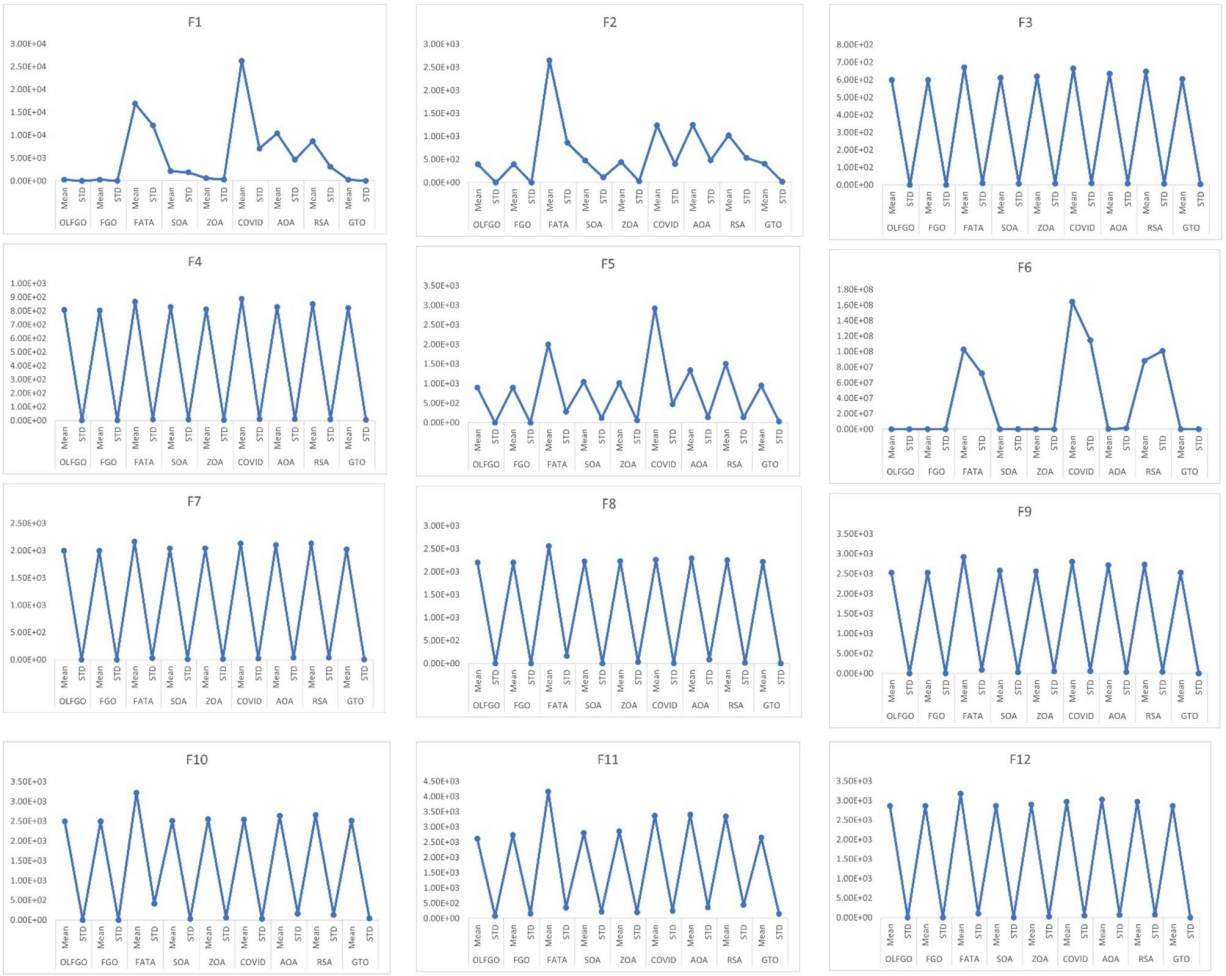
The Line diagrams illustrate OLFGO and its comparison with other algorithms across a CEC’2022 benchmarks with a dimension of 10.

OLFGO really stands out, achieving the best mean fitness values on almost all the functions from F1 to F12. It has zero or very low standard deviations, which indicate high stability and robustness. Especially for F1 to F6, the figures are exactly as well-known optima (i.e., F1 = 300, F2 = 400, etc.), zero standard deviation being a clear indication of good convergence. FGO is closely similar to OLF-GO, especially for F1–F6, but lags a bit in F11, where the mean (2740) is higher than OLFGO (2620). Standard deviations in FGO are slightly higher, indicating less consistency. FATA and COVID are extremely weak on F1, F2, F6 with overwhelmingly large mean values (e.g., FATA on F6 = 1.03E + 08, COVID = 1.64E + 08). The algorithms have extremely high standard deviations, e.g., COVID on F6 has a std of 1.15E + 08, suggesting unstable convergence. RSA, AOA, and FATA are not as competitive and do not do well with hybrid or composition functions like F6, F11, and F12. GTO and ZOA do rather well at a number of functions but are consistently worsted by OLFGO and FGO. For instance, GTO’s performance on F4 (823) and F10 (2520) is better than OLFGO’s (809 and 2500 respectively). SOA does rather well close to OLFGO on F3–F5 but is inaccurate on complicated functions (for instance, F6 and F11) (Fig. 30).

OLFGO performs noticeably better than rival algorithms in terms of accuracy (shown by the lowest mean fitness values) and stability (shown by consistently low standard deviations). The effectiveness of adding the OLS module is confirmed by the baseline FGO’s strong performance, which comes in close second to OLFGO. Algorithms like RSA, FATA, and COVID, on the other hand, exhibit poor reliability, especially on harder benchmark functions. When used in high-dimensional or composite problem settings, SOA, ZOA, and GTO perform moderately but are still less competitive.

#### Computational cost and execution time analysis

The computational complexity of the proposed OLFGO algorithm depends primarily on the population size *N*, the maximum number of iterations *T*, and the problem dimension *D*. In each iteration, the algorithm performs fitness evaluation for all individuals in the population, in addition to the operations introduced by the orthogonal learning mechanism. Therefore, the overall computational complexity can be approximated as:

37 $$\:O(N\times\:T\times\:D)$$

where *N* represents the number of candidate solutions, *T* denotes the maximum number of iterations, and *D* is the number of decision variables (thresholds). The inclusion of orthogonal learning introduces a slight additional overhead; however, it enhances exploration and exploitation capabilities, leading to improved convergence behavior and segmentation accuracy.

#### Execution time comparison

**Fig. 31:**
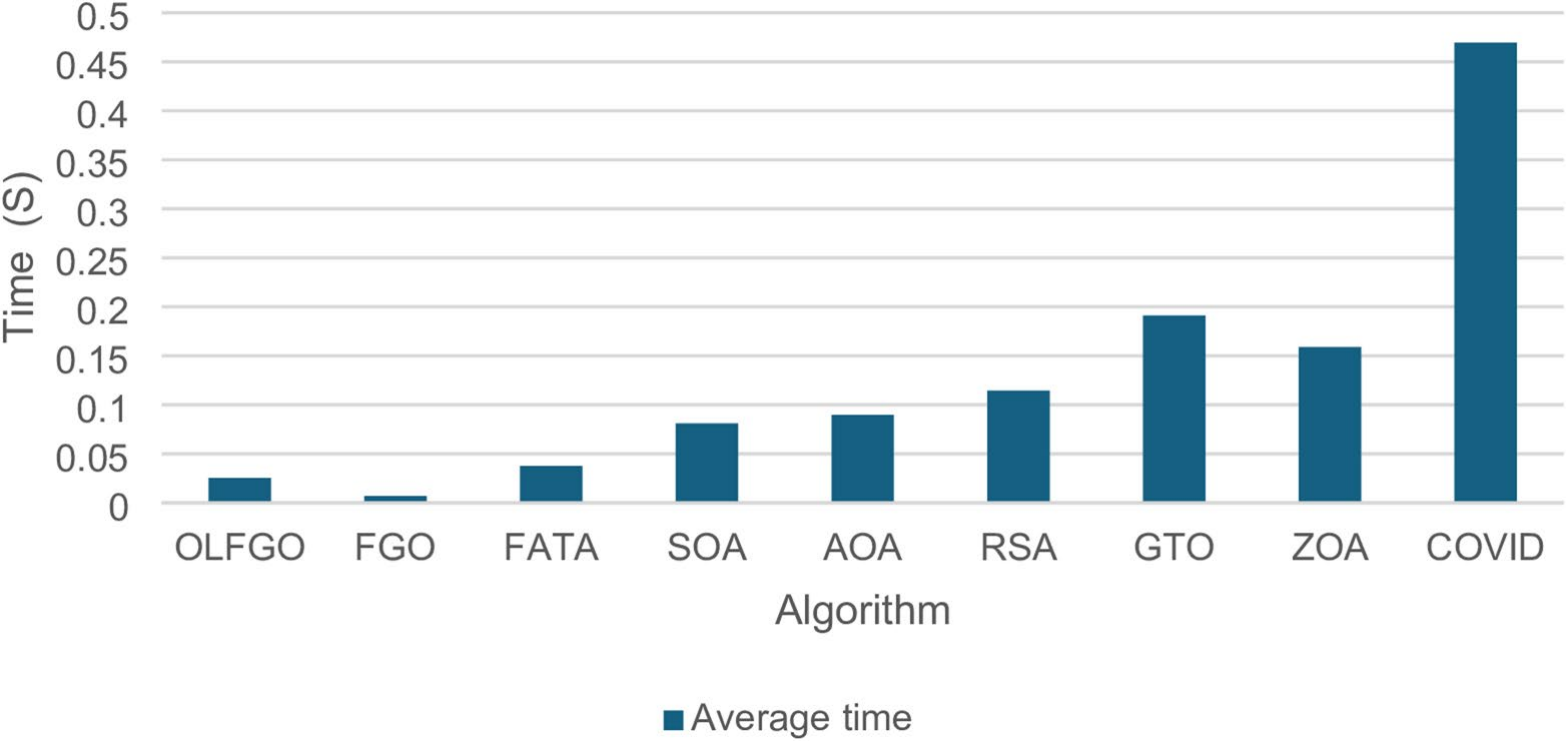
Average run time.

To evaluate the computational efficiency of the proposed method, a fair comparison was conducted between OLFGO and several state-of-the-art optimization algorithms under identical experimental conditions, including the same population size, number of iterations, and hardware configuration. The average execution time (in seconds) over 30 independent runs is shown in Table 10; Fig. 31 as follows:

**Table 10:** Average run time.

Algorithm									
Time (S)	OLFGO	FGO	FATA	SOA	AOA	RSA	GTO	ZOA	COVID
	0.0254	0.0068	0.0376	0.0809	0.0898	0.1144	0.1911	0.1588	0.4695

The results clearly indicate that FGO is the fastest method due to its simpler update mechanism, while OLFGO introduces a moderate computational overhead because of the orthogonal learning strategy. Despite this, OLFGO remains significantly more efficient than most compared algorithms such as SOA, AOA, RSA, GTO, ZOA, and COVID. This demonstrates that the proposed method achieves a favorable trade-off between computational cost and segmentation performance.

#### Discussion

We compared the proposed algorithm, which is called OLFGO by using it to segment 20 Lung cancer CT images. We used six threshold values, which are T-2, T-4, T-6, T-8, T-10 and T-12. The images we used are from a source that is listed as^56^. We chose these images because they have histograms, which helps us see how stable the proposed OLFGO algorithm is. Figure 1 shows some of the images and their histograms. To see how well the OLFGO algorithm works we used some known metrics. These metrics include Peak Signal-to-Noise Ratio which is also called PSNR Structural Similarity Index Measure, which is also called SSIM Feature Similarity Index Measure, which is also called FSIM and Universal Quality Index, which is also called UQI. We used these metrics to test the OLFGO algorithm at threshold levels on the images. We also calculated the Dice coefficient, which is also called Dice to see how similar the segmented results are to the ground truth masks. The Friedman rank sum test was used to compare the performance of the OLFGO algorithm and other optimization algorithms. We compared the proposed OLFGO algorithm to learning-based segmentation models like CNN and Attention U-Net. This comparison shows that the OLFGO algorithm works well and is strong in ways. We also used the CEC 2022 benchmark functions to evaluate the results. The algorithms we compared to the OLFGO algorithm are GTO^49^, RSA^48^, AOA^47^, COVID^46^, ZOA^45^, SOA^44^, FATA^43^, and FGO^9^. The control parameters of these algorithms are the same as suggested in the references except that the max population size’s 50 and the objective function is Otsu’s method. We executed all the algorithms 30 times on one CT image at each threshold value. This was done to have an evaluation of the OLFGO algorithm. Before we started we had to pre-process the dataset. The first step was to resize every image in the lung cancer dataset to a size of 256 × 256 pixels. This resizing is essential because it makes all the inputs the same, which is necessary for processing and analysis by the neural network models. We chose the target size of 256 × 256 because it is a balance, between keeping enough image detail for diagnostic purposes and keeping computational costs low. This balance is important so that the training process of the model can be optimized.

##### Comparison between baseline FGO (without OLS) and OLFGO

The study compares two important versions to determine the role of the orthogonal learning strategy:

Baseline FGO (without OLS): This is the original Fungal Growth Optimization algorithm that uses only lateral branching (Eq. 15) and spore germination (Eq. 16) operators, without any enhancement from orthogonal learning. OLFGO with OLS (full model): This is the proposed hybrid algorithm that adds the orthogonal learning phase (Eqs. 26–27) into the fungal growth framework. In this model, OLS is applied one step after each fungal growth iteration. Quantitative comparison: Table 4 shows that OLFGO consistently outperforms baseline FGO across all threshold levels and all 20 X-ray images. On average, OLFGO improves PSNR by about 15.2% compared to FGO. For example, for IMG11 at T = 12, OLFGO is 19.5102 while FGO is 18.7441. For IMG17 at T = 4, OLFGO is 20.9621 while FGO is 18.0330. The Friedman average rank also reflects this, with OLFGO at 7.93 compared to FGO at 5.28, confirming the full model’s better statistical performance. Key findings from the comparison:

- The addition of OLS helps prevent premature convergence, a common issue in the baseline FGO. This is clear from the more stable results of OLFGO as the threshold levels increase (T = 2 to T = 12). In contrast, FGO shows a decline in performance at higher thresholds.
- The orthogonal learning mechanism allows OLFGO to avoid local optima by thoroughly exploring the search space using factor analysis and orthogonal array construction, a feature missing in baseline FGO.

##### Comparison with other metaheuristics

Table 4 gives the average mean PSNR and the average ranks of each threshold level over all CT images using the Friedman test. As seen from the table, the proposed algorithm performed better than all existing algorithms. It has not only been able to perform better than the traditional FGO but also all other algorithms. SOA is regarded as the second best-performing algorithm as, apart from a few threshold levels, it has been proved to be better than any other algorithm. AOA can be considered to be the worst-performing algorithm among all the algorithms being used. To present the results graphically, Fig. 2 presents the PSNR value of Table 4 while Fig. 3 presents the average rank of the same data. By analyzing these two figures, we can conclude that OLFGO performs optimally compared to other algorithms. SOA is the next best algorithm after OLFGO and AOA is the worst performing algorithm. In addition to the PSNR and average rank, the SSIM, FSIM, UQI and DICE are also computed for each threshold level over all CT images. Tables 5, 6, 7 and 8 give these values and their graphical representations are shown in Figs. 4, 5, 6 and 7. The above tables indicate that the OLFGO algorithm has the best performance compared to other algorithms, and this is illustrated in Figs. 4, 5, 6 and 7. It can be concluded from the above observation that OLFGO is the most efficient algorithm for segmenting the CT images to ensure quick interpretation and accurate identification of lung cancer, since the incorporation of OLS in the FGO search process helps prevent early convergence (by improving the PSNR by 15%). The limitations of the OLFGO algorithm indicate that the algorithm performs poorly when processing very noisy images (e.g., IMG8, using 12 thresholds).

##### Comparison with deep learning-based models

Regarding the comparison between the suggested OLFGO model and deep learning-based models such as CNN and Attention U-Net, Fig. 29 demonstrates an evident disparity in performance. While the segmentation results produced by the OLFGO algorithm demonstrate improved image quality and resemblance to ground-truth masks compared to those generated by the CNN and Attention U-Net algorithms, OLFGO provides higher accuracy and reliability in segmentation. The improved performance of OLFGO compared to deep learning techniques can be justified due to: (i) lack of need for vast amounts of labeled training datasets, (ii) adaptive search approach that does not depend on predetermined feature extractors, and (iii) orthogonal learning procedure that exhaustively searches through the threshold space without falling victim to overfitting problems related to training distributions.

## Integration of the proposed method into the radiology workflow

The proposed OL-FGO based multilevel thresholding framework is intended to serve as a computer-aided diagnosis (CAD) tool for lung cancer identification and segmentation in CT images. In the radiology workflow, the technique operates after CT image acquisition and fundamental preprocessing procedures such noise removal and intensity normalization.

In a typical diagnostic process, CT images are captured and saved in DICOM format in the hospital Picture Archiving and Communication System (PACS). Then, the suggested OL-FGO segmentation module receives the preprocessed images automatically. At this point, the system optimizes intensity segmentation using the FGO search approach improved with orthogonal learning, performing multilevel thresholding to identify suspicious lung regions. The system’s output includes the segmented lung tumor image showing the boundaries of the lesions. Finally, the proposed framework is intended to provide radiologists with visual evidence thereby improving diagnostic accuracy.

## Limitations

In this paper, we proposed OLFGO as a tool for efficiently segmenting lung cancer images. as discussed in the previous sections, the proposed approach showed to be effective for this task. However, there are some shortcomings that need to be addressed. The utilized dataset represents a preliminary experimental study to validate the proposed method. However, the proposed algorithm needs to be validation on larger public datasets.

The limitations also include reliance on grayscale thresholding. In medical imaging, color information actually carries meaning and can significantly improve analysis and diagnoses. For these reasons, the proposed algorithm should be applied to color medical images as a refinement of the proposed work in order to further enhance lung cancer diagnoses. Additionally, future work should address variability in CT images due to differences in scanners, acquisition protocols, noise levels, and slice thickness.

## Conclusion and future work

This paper proposes a more efficient metaheuristic algorithm, referred to as Orthogonal Learning-based Fungal Growth Optimizer (OLFGO), with the purpose of improving the performance of the original FGO algorithm by proposing an orthogonal learning technique. Fungal growth tendencies in nature, specifically hyphal tip growth enlargement, branching, and spore germination, are used as the basis of the exploration and exploitation processes of the algorithm. The addition of the orthogonal learning mechanism enables OLFGO to generate and search diverse offspring solutions in an orderly manner by constructing orthogonal arrays, so as to improve the convergence performance and solution diversity during optimization. To confirm the effectiveness of the proposed OLFGO, two experimental experiments were conducted. Firstly, the algorithm was tested on 10-dimensional CEC2022 test functions to check the global optimization capability. The derived mean and standard deviation indicate that OLFGO is competitive and more stable compared to certain state-of-the-art and newly proposed metaheuristics. This confirms the stability of the algorithm and its ability to achieve good balance between exploration and exploitation in multimodal complex landscapes. Second, OLFGO was implemented for an actual problem in medical image segmentation for the diagnosis of lung cancer by using multilevel thresholding. 20 chest CT images were used in the database, and segmentation performance was evaluated at various threshold values (2, 4, 6, 8, 10, and 12) using typically used performance measures: PSNR, SSIM, FSIM, UQI and DICE to quantify the spatial overlap between the predicted segmentation and the ground truth. For a comprehensive evaluation, the proposed method is compared against state-of-the-art deep learning-based segmentation models such as Attention U-Net, and convolutional neural network (CNN)-based segmentation approaches. These deep learning models are widely recognized benchmarks in medical image analysis due to their strong performance in learning hierarchical features from data. Experimental results reveal that OLFGO outperforms reference algorithms on all metrics, producing high-quality segmentation results with good structure and fine image details critical to accurate diagnosis. Furthermore, Friedman rank sum test was applied to statistically rank the performance of OLFGO and reference compared algorithms where OLFGO achieved the highest rank, which confirms its better segmentation performance. Despite its exceptional performance, its current version of OLFGO remains vulnerable to a couple of limitations. Specifically, the application of orthogonal learning, as successful as it has been, adds modestly computational time due to additional offspring evaluation and array construction, increases convergence rate, Achieves better balance between exploration and exploitation and yields more accurate segmentations. The Orthogonal Learning Strategy (OLS) plays an important role in increasing solution diversity and preventing early convergence. Moreover, even though the algorithm avoids premature con-vergence in most cases, it still has room for improvement for certain high-dimensional or noisy data sets. Furthermore, in future research work, we plan to address these problems by integrating adaptive methods such as dynamic learning rates, opposition-based learning, and Lévy flight perturbation to improve efficiency along with solution accuracy. In addition, we plan to extend OLFGO to support binary and multi-objective variants so that it could be used in more optimization problems, such as medical feature selection, multi-class segmentation, and real-time clinical decision-making systems. Finally, we plan to enhance the performance for deep learning models using OLFGO.

## Data Availability

Data Used for this work is available at [https://www.kaggle.com/datasets/hamdallak/the-iqothnccd-lung-cancer-dataset] (https:/www.kaggle.com/datasets/hamdallak/the-iqothnccd-lung-cancer-dataset) , (accessed on 15 October 2025).
